# Sc(OTf)_3_-Mediated [4 + 2] Annulations of *N*-Carbonyl Aryldiazenes with Cyclopentadiene to Construct Cinnoline Derivatives: Azo-Povarov Reaction

**DOI:** 10.1021/acs.joc.2c01224

**Published:** 2022-08-16

**Authors:** Xabier Jiménez-Aberásturi, Francisco Palacios, Jesús M. de los Santos

**Affiliations:** Department of Organic Chemistry I, Faculty of Pharmacy and Lascaray Research Center, University of the Basque Country (UPV/EHU), Paseo de la Universidad 7, Vitoria 01006, Spain

## Abstract

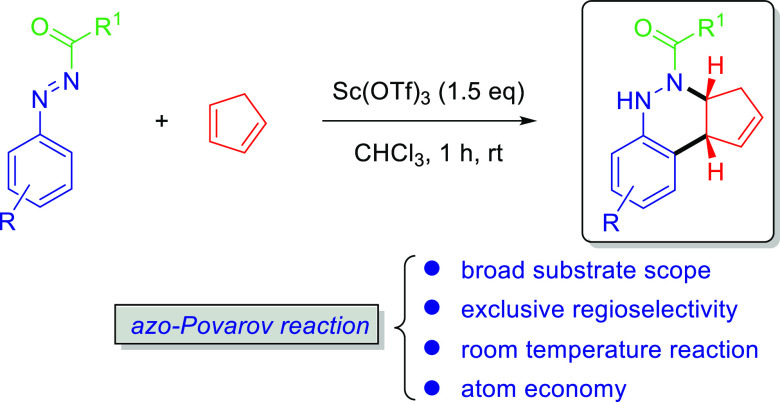

We disclose the first accomplishment of the azo-Povarov reaction involving Sc(OTf)_3_-catalyzed [4 + 2] annulations of *N*-carbonyl aryldiazenes with cyclopentadiene in chloroform, in which *N*-carbonyl aryldiazenes act as 4π-electron donors. Hence, this protocol offers a rapid access to an array of cinnoline derivatives in moderate to good yields for substrates over a wide scope. The synthetic potential of the protocol was achieved by the gram-scale reaction and further derivatization of the obtained polycyclic product.

## Introduction

**Figure 1:**
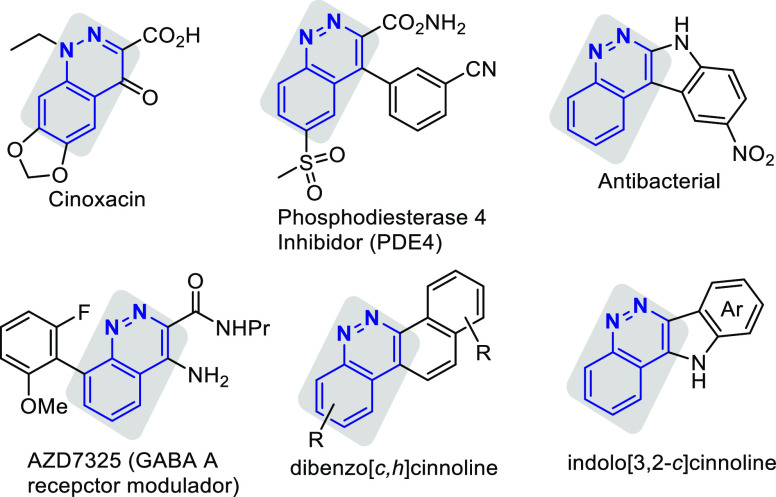
Selected pharmaceutically and bioactive cinnoline derivatives.

The cinnoline ring is an important structural subunit found in a wide range of compounds of significant pharmacological and chemical importance.^1^ Cinnoline derivatives display a broad spectrum of pharmacological activities, such as anticancer, antibacterial, antimicrobial, antifungal, anti-inflammatory, antimalarial, antiparasitic, and analgesic activity (Figure 1). For instance, Cinoxacin^2^ is a synthetic antibiotic from the quinolone group used to treat urinary tract infections. On the other hand, some compounds with a cinnoline structure are found in preclinical tests, such as compound AZD7325,^3^ a modulator of GABA_A_ receptors that exhibits a powerful anxiolytic effect (Figure 1). In particular, benzo[*c*]cinnolines are considered as important structures in medical chemistry due to the promising anticancer activities they possess. Several cinnoline derivatives such as dibenzo[*c*,*h*]cinnolines^4^ or indolo[3,2-*c*]cinnolines^5^ have been identified as potent anticancer agents and kinase inhibitors (Figure 1). In fact, the dibenzo[*c*,*h*]cinnolines are also topoisomerase I inhibitors and possess significant cytotoxic activity.^4^ The fact that none of these benzo[*c*]cinnolines is found in nature makes these skeletons highly interesting in organic synthesis.

**Scheme 1:**
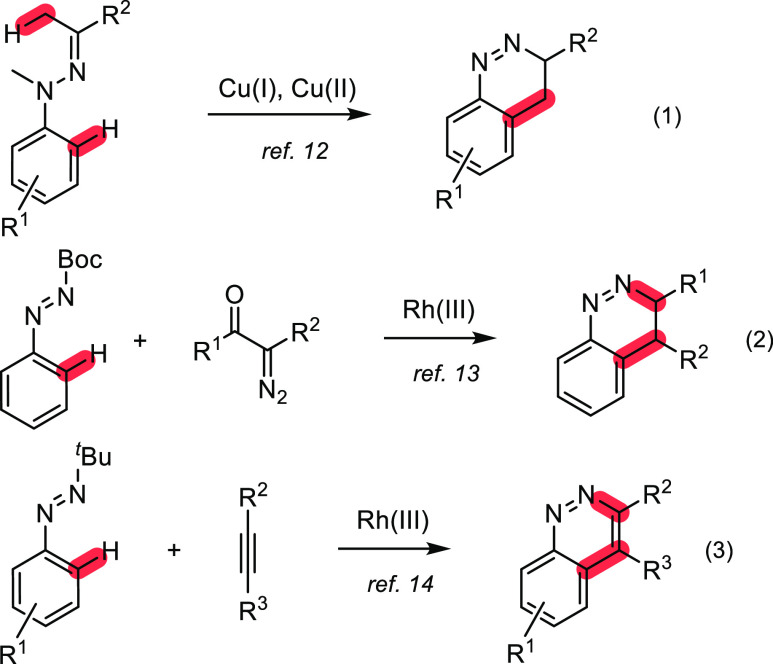
Transition Metal-Catalyzed Synthesis of Cinnolines

However, traditional cinnoline access strategies such as intermolecular cycloaddition involving prefunctionalization of nitriles,^6^ aryl hydrazines,^7^ and aryl hydrazones,^8^ or cyclization of phenyldiazonium ions with highly active triazenes *ortho* to a terminal phenylacetylene,^9^ often involve a very limited synthetic scope and multi-stage reaction sequences and cannot represent a general synthetic method. The activation of the C–H bond catalyzed by transition metals is not only an important strategy in the synthesis and modification of heterocyclic systems,^10^ but also one of the most valuable methods for the preparation of the cinnoline backbone^11^ due to the high degree of regioselectivity, atom economy, and reaction stages. For instance, in 2012, Ge et al.^12^ developed a copper-promoted intramolecular dehydrogenative cyclization of *N*-methyl-*N*-phenylhydrazones to afford cinnolines through C(sp^3^)H-oxidation, cyclization, and aromatization sequence (Scheme 1, eq 1). In 2016, Yao and Lin’s group^13^ reported a rhodium-catalyzed redox-neutral annulation reaction between diazo and azo compounds for the preparation of cinnolines under mild conditions (Scheme 1, eq 2). Cinnolines have also been synthesized by Rh(III)-catalyzed C–H bond activation and cyclization of *N*-*tert*-butyl-aryldiazenes with alkynes (Scheme 1, eq 3).^14^

**Scheme 2:**
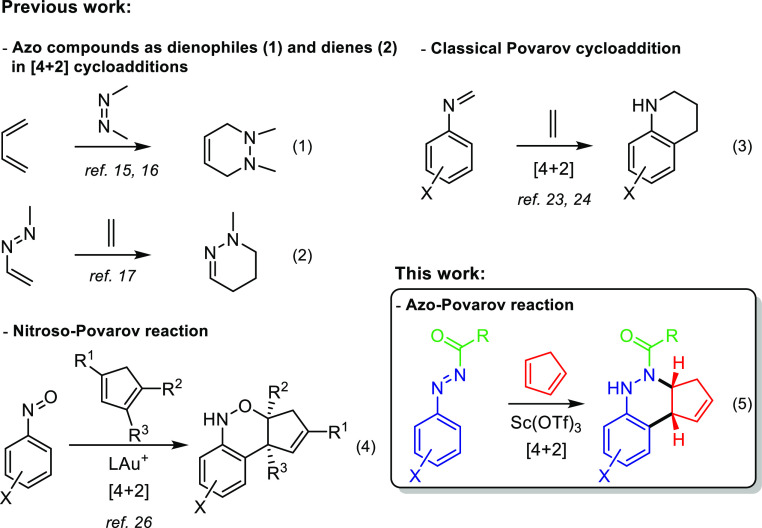
Annulations of Imines, Nitrosoarenes, and *N*-Carbonyl Aryldiazenes with Dienes

Furthermore, azo compounds, valuable synthetic building blocks, have been widely used in cycloaddition reactions with a variety of partners for the preparation of many nitrogen-containing heterocyclic compounds. For example, they have been widely used not only as dienophiles in azo hetero-Diels–Alder reactions^15,16^ [Scheme 2, eq 1)] but also as dienes in [4 + 2] cycloaddition reactions [Scheme 2, eq 2].^17^ Conjugated azoalkenes have proven to be valuable starting materials for the synthesis of a plethora of multi-nitrogen-containing heterocycles and in target-oriented synthesis of naturally occurring and biologically active compounds.^18^ We have reported the usefulness of phosphorous-substituted azoalkenes for the preparation of α-amino phosphonates,^19^ functionalized mercapto diketones,^20^ and heterocyclic compounds.^21,22^

The classical Povarov reaction^23^ between an aldimine, generated by the condensation of an aromatic amine and an aldehyde, and the olefinic or acetylenic component entails a useful tool for the construction of carbon–carbon and carbon-heteroatom bonds and the generation of six-membered rings with high molecular complexity. In this way, the Povarov reaction between imines and dienes is well known [Scheme 2, eq 3].^24^ Our group has established the necessary methodology for the preparation and development of various TopI inhibitors with application as antiproliferative compounds using the Povarov reaction.^25^ Very recently, an enantioselective gold-catalyzed [4 + 2]-annulation of nitrosoarenes and cyclopentadiene derivatives (nitroso-Povarov reaction) has been reported [Scheme 2, eq 4].^26^

To continue our interest in new azoalkene reactions, as depicted in [Scheme 2, eq 5], herein, we report the first [4 + 2] annulation reaction (azo-Povarov reaction) between *N*-carbonyl aryldiazenes and cyclopentadiene, in which the *N*-carbonyl aryldiazene acts as the 4π-electron (diene) system like *N*-arylimines in Povarov reactions.

## Results and Discussion

As outlined in Table 1, we started our investigation with the optimization of the reaction conditions with aryldiazene carboxylate **2a** as the model substrate and cyclopentadiene. Aryldiazene carboxylates are prepared by oxidation of aromatic hydrazine derivatives **1** using *N*-bromosuccinimide (NBS)/Py.^27^ The effect of the catalyst on the azo-Povarov reaction was first evaluated with chloroform as the solvent. With ZnCl_2_ as the catalyst, the reaction proceeded smoothly at room temperature to give the product **3a** in a 49% yield (entry 1). However, only a 9% yield of **3a** could be achieved when Ag(OTf) was used as the catalyst in this process (entry 2). Magnesium catalysts, such as MgBr_2_·Et_2_O and Mg(ClO_4_)_2_, are not suitable for the current reaction since cinnoline **3a** could not be detected, and only the starting aryldiazene carboxylate **2a** was recovered instead (entries 3 and 4). A higher reaction temperature (55 °C) or longer reaction time (48 h) did not improve these results (entries 5 and 6). The use of RuCl_3_ as the catalyst gave similar results (entry 7). To our surprise, since MgBr_2_ afforded cinnoline **3a** in 71% yield, BF_3_·Et_2_O, recently used by our group as the catalyst in the classical Povarov reaction,^25^ only gave a 39% yield of **3a** (entries 8 and 9). The use of Yb(OTf)_3_ (entry 10) was also unsuitable for this transformation. After a series of optimization experiments, we identified Sc(OTf)_3_ as the optimal catalyst. On performing the reaction at room temperature, product **3a** was isolated in 91% yield after 1 h reaction time (entry 11). None of the other catalyst candidates that we explored (Cu(OTf)_2_ or InCl_3_) performed any better (entries 12 and 13). When examining the catalyst loading, the use of 1.5 equiv of catalyst seems necessary in the current reaction since we observed no reaction when different equivalents between 0.2 and 1.0 of Cu(OTf)_2_, InCl_3_, ZnCl_2_, or Sc(OTf)_3_ as Lewis acids were used.

**Table 1:**
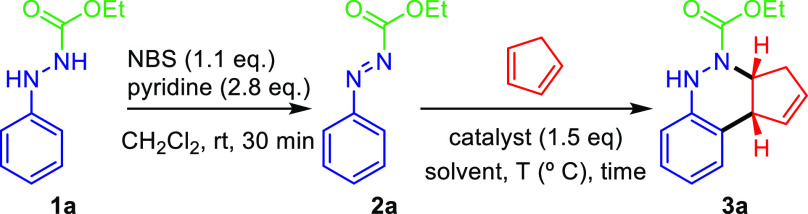
Reaction Condition Optimization

entry^a^	catalyst	time (h)	*T* (° C)	solvent	yield (%)^b^
1	ZnCl_2_	5	rt	CHCl_3_	49
2	Ag(OTf)	4	rt	CHCl_3_	9
3	MgBr_2_·Et_2_O	19	rt	CHCl_3_	0
4	Mg(ClO_4_)_2_	19	rt	CHCl_3_	0
5	MgBr_2_·Et_2_O	48	55	CHCl_3_	0
6	Mg(ClO_4_)_2_	48	55	CHCl_3_	0
7	RuCl_3_	24	rt	CHCl_3_	0
8	MgBr_2_	24	rt	CHCl_3_	71
9	BF_3_·Et_2_O	1	rt	CHCl_3_	39
10	Yb(OTf)_3_	6	rt	CHCl_3_	0
11	Sc(OTf)_3_	1	rt	CHCl_3_	91
12	Cu(OTf)_2_	3	rt	CHCl_3_	21
13	InCl_3_	2	rt	CHCl_3_	55
14	Sc(OTf)_3_	17	rt	THF	6
15	Sc(OTf)_3_	0.5	rt	DCM	87
16	Sc(OTf)_3_	1	rt	MeCN	57
17	Sc(OTf)_3_	0.5	rt	MeOH	0
18	Sc(OTf)_3_	24	rt	H_2_O	0
19	Sc(OTf)_3_	15	50	H_2_O	0
20	Sc(OTf)_3_	c	rt	CHCl_3_	60

a Unless otherwise noted, reactions were conducted on a 0.5 mmol scale, catalyst (1.5 equiv), and solvent (3 mL).

b Isolated yields.

c The reaction was performed using **2a** (0.5 mmol), catalyst (1.5 equiv), and solvent (1 mL) for 15 min.

The effect of the solvent on the azo-Povarov reaction was also studied. When performed with THF as the solvent, the reaction afforded traces of **3a** (entry 14). In addition, using dichloromethane (DCM) as the solvent and lowering the reaction time to 0.5 h, led to similar results as before (compare entries 11 and 15). Starting aryldiazene carboxylate **2a** was not fully consumed with MeCN (entry 16), whereas polar solvents such as MeOH (entry 17) and H_2_O (entries 18 and 19) do not work in the azo-Povarov reaction. Finally, lowering the reaction time to 15 min using chloroform as the solvent gave **3a** with a moderate yield of 60% (entry 20).

**Scheme 3:**
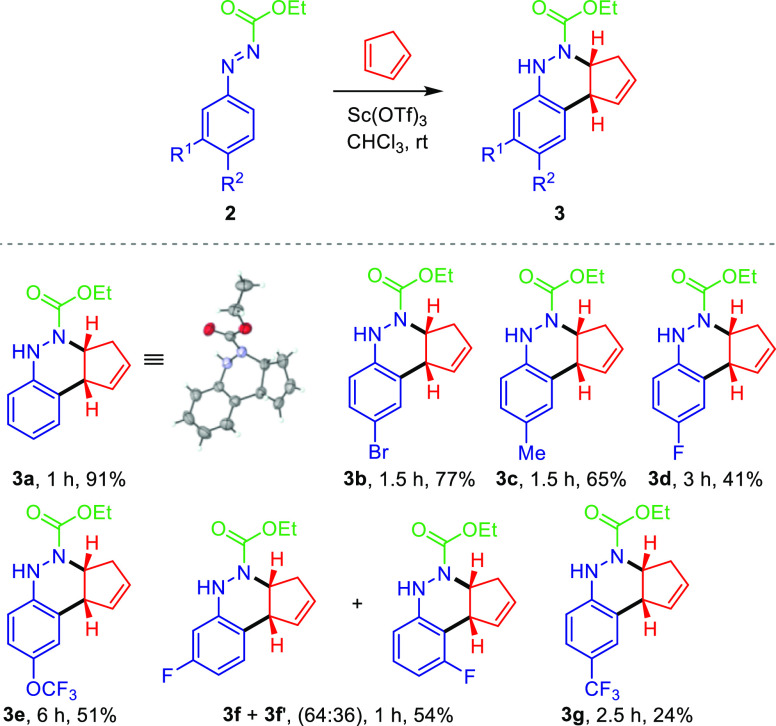
Substrate Scope of the Aromatic Ring in Aryldiazene Carboxylates in the Azo-Povarov Reaction with Cyclopentadiene See the Supporting Information for experimental details.

With the optimal reaction conditions in hand, next we explored the substrate scope of the aromatic ring of aryldiazenes in the azo-Povarov reaction, and the results are summarized in Scheme 3. A large selection of functional groups at the aromatic ring in aryldiazene carboxylates **2** was well-tolerated. Both electron-donating (Me) and electron-withdrawing groups (Br, F, OCF_3_, and CF_3_) at the *para*-phenyl position afford desired products **3c**, **3b**, **3d**, **3e,** and **3g** in 24–77% yields. Among them, 4-bromo derivative **3b** and 4-methyl derivative **3c** were achieved with the best yields (77% and 65%, respectively). Nevertheless, *meta*-phenyl diazene **2f** (R^1^ = F, R^2^ = H) afforded a 64:36 mixture of azo-Povarov adducts **3f** and **3f**′ in 54% yield. The structure of **3a** has been unambiguously determined by X-ray diffraction. The CIF data are presented in the Supporting Information, and the ORTEP drawing of **3a** is shown in Scheme 3.

**Scheme 4:**
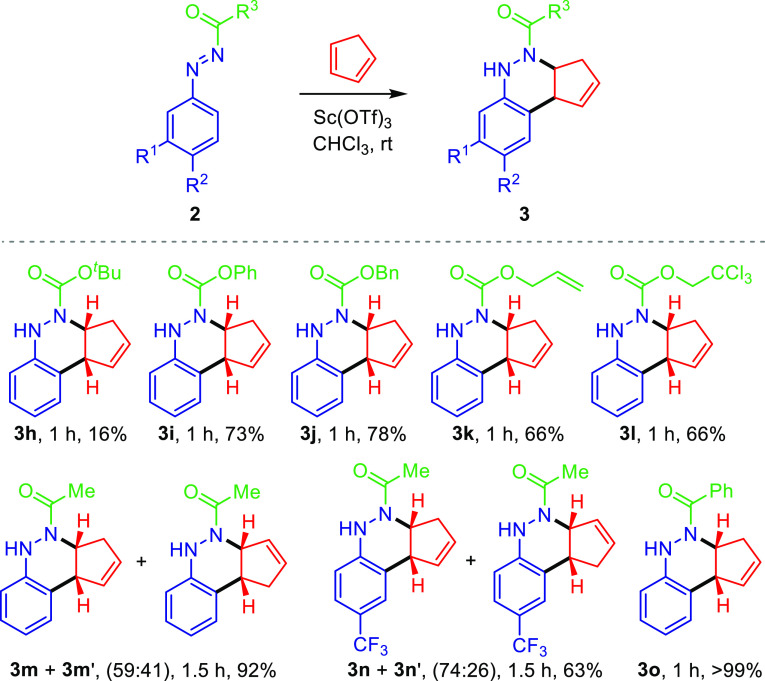
Substrate Scope Varying Functional Group R^3^ at the Nitrogen Atom of *N*-Carbonyl Aryldiazenes See the Supporting Information for experimental details.

Stimulated by the above obtained results on the azo-Povarov reaction between aryldiazene carboxylates **2** and cyclopentadiene, we further investigated the substrate scope varying the functional group (R^3^) at the nitrogen atom of the aryldiazenes **2** (Scheme 4). To our delight, it was found that the reaction proceeded smoothly using the same reaction conditions, and starting aryl **2i** or alkyl aryldiazene carboxylates **2h** and **2j**–**2l** afforded cinnoline derivatives **3i** or **3h** and **3j**–**3l**, respectively. For instance, the Sc(OTf)_3_-catalyzed [4 + 2] annulation reaction of phenyl aryldiazene carboxylate **2i** (R^3^ = OPh) with cyclopentadiene afforded adduct **3i** in 73% yield. *N*-Boc aryldiazene **2h** (R^3^ = O^*t*^Bu) did not perform well since only 16% of cinnoline **3h** was obtained. Nevertheless, annulations of other alkyl aryldiazene carboxylates (**2j**–**2l**) bearing R^3^ = OBn (*N*-Cbz), Oallyl (*N*-Alloc), and 2,2,2-trichloroethoxy (*N*-Troc) moieties delivered cinnoline derivatives **3j**–**2l** in 66–78% yields. Conversely, when *N*-acetyl aryldiazene **2m** (R^3^ = Me) derived from *N*′-phenylacetohydrazide was used in the azo-Povarov reaction, a 59:41 mixture of regioisomers **3m** and **3m**′ was observed in 92% yield. Similar results were attained with *p*-CF_3_ phenyl diazene **2n**, and the corresponding adduct was obtained as a 74:26 regioisomeric mixture in 63% yield. Interestingly, only one regioisomer **3o** in almost quantitative yield has been observed when *N*-benzoyl aryldiazene **2o** (R^3^ = Ph) derived from *N*′-phenylbenzohydrazide was used in the current reaction. On comparing aryl and alkyl aryldiazene carboxylates **2h**–**l** with *N*-acyl or *N*-benzoyl aryldiazenes **2m**–**o**, better chemical yields were obtained for cinnolines derived from the latter (Scheme 4).

**Scheme 5:**
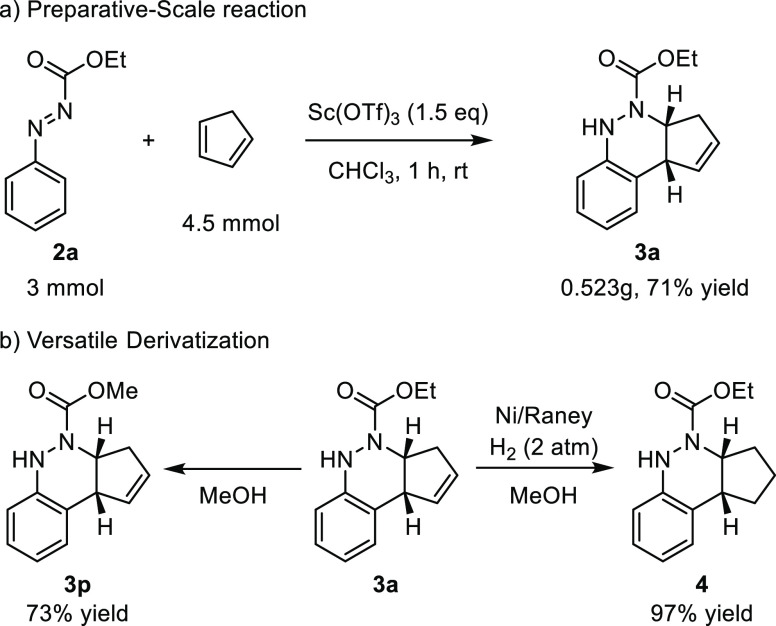
Gram-Scale Synthesis and the Further Transformation of **3a**

The gram-scale synthesis and further derivatization of **3a** have been accomplished, as shown in Scheme 5. The use of 3.0 mmol of aryldiazene carboxylate **2a** could give cinnoline derivative **3a** in 71% yield (0.523 g) under the standard conditions. In addition, transesterification of **3a** produced desired cinnoline **3p** in 73% yield in the presence of MeOH, and the reduction of the carbon–carbon double bond in **3a** was affected with hydrogenolysis using Ni/Raney as the catalyst affording product **4** in 97% yield.

**Scheme 6:**
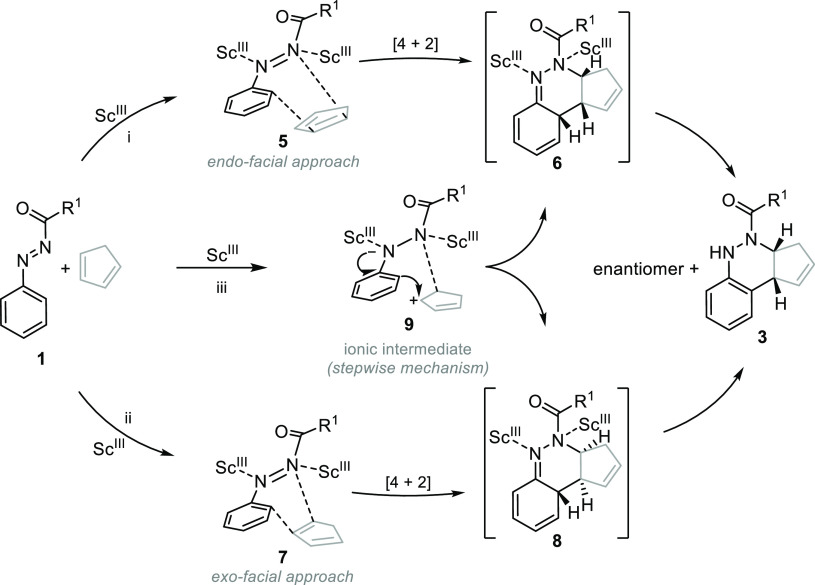
Proposed Mechanism Pathways for the Azo-Povarov Reaction

A plausible mechanism for this Sc-catalyzed azo-Povarov reaction has been outlined in Scheme 6. Controversy exists about the mechanism of the Povarov reaction, suggesting a concerted mechanism^28^ as well as evidences for a stepwise mechanism^29^ have also arisen. Based on the stereochemistry of isolated compounds **3**, a mechanism with both *endo*- or *exo*-π-facial approach would explain the formation of these cinnolines **3**. Thus, the *endo*-facial approach of cyclopentadiene to **1** could lead to the [4 + 2] intermediate **6** and then the corresponding cycloadduct **3** (Scheme 6, approach from **5**, pathway i). However, the *exo*-facial approach of cyclopentadiene to **1** (Scheme 5, approach from **7**, pathway ii) would afford the same cinnoline **3** through intermediate cycloadduct **8**. Conversely, as observed previously for the Povarov reaction between aryl imines and 1,3-dienes,^29^ a stepwise reaction mechanism through ionic intermediate **9** (Scheme 6, pathway iii) cannot be discarded.

## Conclusions

In summary, we have developed a novel strategy to efficiently access the cinnoline scaffold from the [4 + 2] annulation reaction of *N*-carbonyl aryldiazenes and cyclopentadiene catalyzed by scandium triflate [Sc(OTf)_3_]. This success represents the first example of the azo-Povarov reaction. Most of these [4 + 2]-annulations proceeded smoothly in chloroform for 1 h, giving exclusive regioselectivity and satisfactory yields. This protocol was applicable to a wide range of substrates, including various aryldiazenes with electron-donating (Me) and electron-withdrawing groups (Br, F, OCF_3_, and CF_3_) at the *para*-phenyl position and different aryldiazenes with reactive functional groups at the nitrogen atom. The synthetic potential of this azo-Povarov protocol was demonstrated with the preparative–scale reaction and versatile synthetic transformations of the product. This strategy for the preparation of the cinnoline backbone entails a valued method due to the atom economy, reaction stages, and high degree of regioselectivity. Further synthetic applications of this methodology are underway in our group and will be reported in due course.

## Experimental Section

### General Experimental Information

Solvents for extraction and chromatography were of technical grade. All solvents used in reactions were freshly distilled and dried over molecular sieves of 3 Å before use. All other solvents and reagents were obtained from commercial sources and recrystallized or distilled as necessary or used without further purification. All reactions were performed under an atmosphere of dry nitrogen. Melting points are uncorrected. IR spectra were measured on the Nicolet iS10 Thermo Fisher Scientific spectrometer as neat solids. Absorbance frequencies are given at the maximum intensity in cm^–1^. High-resolution mass spectra (HRMS) were obtained by the positive-ion electrospray ionization (ESI) method with a time-of-flight Q-TOF system. Data are reported in the form of *m*/*z* (intensity relative to base = 100). ^1^H (300, 400 MHz), ^13^C (75, 100 MHz), and ^19^F (282, 376 MHz) spectra were recorded on Varian Unity Plus (300 MHz) or Bruker Avance 400 (400 MHz) spectrometers, respectively, in CDCl_3_, as specified below. Chemical shifts (δ_H_) are reported in parts per million (ppm) with the internal chloroform signal at 7.24 ppm as the standard for ^1^H NMR. Chemical shifts (δ_C_ and δ_F_) are reported in parts per million (ppm) with the internal chloroform signal at 77.0 ppm for ^13^C NMR and the external trichlorofluoromethane (Cl_3_CF) signal at 0.0 ppm as the standard for ^19^F NMR. All coupling constant (*J*) values are given in Hz. ^19^F and ^13^C NMR spectra were recorded in a broad band decoupling mode from hydrogen nuclei. Distortionless enhanced polarization transfer supported peak assignments for ^13^C NMR. The data is being reported as (s = singlet, d = doublet, t = triplet, q = quartet, m = multiplet, dd = double doublet, bs = broad singlet). Chromatographic purification was performed as flash chromatography using commercial grades of silica gel finer than 230 mesh with pressure or neutral aluminum oxide. Analytical thin layer chromatography (TLC) was performed on precoated Merck silica gel 60 F_254_ TLC aluminum plates, and spot visualized with UV light or permanganate stain. Functionalized hydrazines **1h** and **1m** are commercially available. However, functionalized hydrazines **1a**,^30^**1b**,^30,31^**1c**,^30^**1d**,^30^**1f**,^30,31^**1g**,^30^**1i**,^30,32^**1j**,^30,31^**1k**,^30,33^**1l**,^30,31^**1n**,^34,35^ and **1o**(34) and *N*-carbonyl aryldizenes **2a**,^30^**2b**,^30,31^**2c**,^30^**2d**,^30^**2f**,^30,31^**2g**,^30^**2h**,^30,31^**2i**,^30,32^**2j**,^30,31^**2k**,^30,33^**2l**,^30,31^**2m**,^30,36^ and **2o**(30,31) were prepared according to literature procedures.

### Experimental Procedure and Characterization Data for Compounds **1–2**

#### General Procedure and Spectral Data for Functionalized Hydrazines **1**

To a 0 °C stirred solution of the corresponding hydrazine hydrochloride (15 mmol) in CH_3_CN (30 mL) and pyridine (2.5 mL, 31.5 mmol), ethyl chloroformate (1.6 mL, 16.5 mmol) was added dropwise. The reaction mixture was stirred for 15 min at 0 °C and then for 3 h at room temperature. Water (30 mL) was added and the resulting mixture was acidified with HCl (6 M) to pH 4–6. The crude product was extracted with CH_2_Cl_2_ (5 × 15 mL). The combined organic layers were washed with saturated aqueous NaHCO_3_ (15 mL) and brine (15 mL), dried over anhydrous MgSO_4_, filtered, and concentrated to dryness in a vacuum. The crude product was purified by recrystallization from an appropriate solvent, as indicated in the literature procedure,^30^ to afford functionalized hydrazines **1**.

#### Ethyl 2-(4-(trifluoromethoxy)phenyl)hydrazine-1-carboxylate (**1e**)

Following the literature procedure,^30^**1e** was obtained as a brown solid (80% yield). mp: 89–91 °C; IR (neat) *v*_max_ 3102, 3048, 2991, 1712, 1682 cm^–1^; ^1^H NMR (400 MHz, CDCl_3_) δ (ppm): 7.04 (d, *J* = 8.8 Hz, 2H), 6.78 (bs, 1H), 6.74 (d, *J* = 8.8 Hz, 2H), 6.07 (bs, 1H), 4.16 (q, *J* = 7.1 Hz, 2H), 1.29–1.19 (m, 3H); ^13^C {^1^H} NMR (75 MHz, CDCl_3_) δ (ppm): 157.3, 146.7, 142.9 (q, ^3^*J*_CF_ = 1.7 Hz), 122.2, 120.5 (q, ^1^*J*_CF_ = 254.3 Hz), 113.5, 62.1, 14.4; ^19^F NMR (282 MHz, CDCl_3_) δ (ppm): −58.8. ESI-HRMS (CI) *m*/*z*: calcd for C_10_H_12_F_3_N_2_O_3_ ([M + H]^+•^), 265.0800; found, 265.0804.

#### General Procedure and Spectral Data for *N*-Carbonyl Aryldiazenes **2**

To a stirred solution of the corresponding functionalized hydrazines **1** (3 mmol) in CH_2_Cl_2_ (21 mL), pyridine (0.68 mL, 8.4 mmol) was added. Then, NBS (599 mg, 3.30 mmol) was added portionwise. The reaction mixture was stirred for 30 min and then washed with aqueous HCl (5%, 30 mL), aqueous sodium thiosulfate (1.5%, 15 mL), saturated aqueous NaHCO_3_ (30 mL), and brine (30 mL). The organic layer was dried over anhydrous MgSO_4_, filtered, and concentrated to dryness in a vacuum to give pure aryl and alkyldiazene carboxylates **2**, as indicated in the literature procedure.^30^

#### Ethyl 2-(4-(trifluoromethoxy)phenyl)diazene-1-carboxylate (**2e**)

Following the literature procedure,^30^**2e** was obtained as a red oil (95% yield) and was used immediately without purification in the next reaction step. IR (neat) *v*_max_ 3081, 2987, 2940, 1761 cm^–1^; ^1^H NMR (300 MHz, CDCl_3_) δ (ppm): 7.94 (d, *J* = 8.9 Hz, 2H), 7.31 (d, *J* = 8.9 Hz, 2H), 4.48 (q, *J* = 7.1 Hz, 2H), 1.42 (t, *J* = 7.1 Hz, 3H); ^13^C {^1^H} NMR (75 MHz, CDCl_3_) δ (ppm): 161.8, 152.9 (q, ^3^*J*_CF_ = 1.7 Hz), 149.4, 125.4, 121.0 (q, ^4^*J*_CF_ = 0.8 Hz), 120.2 (q, ^1^*J*_CF_ = 257.7 Hz), 64.5, 14.0; ^19^F NMR (282 MHz, CDCl_3_) δ(ppm): −58.2.

#### 1-((4-(Trifluoromethyl)phenyl)diazenyl)ethan-1-one (**2n**)

Following the literature procedure,^30^**2n** was obtained as a red oil (<99%) and was used immediately without purification in the next reaction step. ^1^H NMR (400 MHz, CDCl_3_) δ (ppm): 7.94 (d, *J* = 8.7 Hz, 2H), 7.77 (d, *J* = 8.7 Hz, 2H), 2.42 (s, 3H); ^13^C {^1^H} NMR (75 MHz, CDCl_3_) δ (ppm): 188.1, 153.0, 134.4 (q, ^2^*J*_CF_ = 32.8 Hz), 126.6 (q, ^3^*J*_CF_ = 3.7 Hz), 123.6, 123.4 (q, ^1^*J*_CF_ = 273.7 Hz), 21.2; ^19^F NMR (376 MHz, CDCl_3_) δ (ppm): −62.9.

### Experimental Procedure and Characterization Data for Functionalized Cinnolines **3**

To a stirred solution of the corresponding *N*-carbonyl aryldiazene **2** (0.5 mmol, 1 equiv) in CHCl_3_ (3 mL), was added cyclopentadiene (63 μL, 0.75 mmol, 1.5 equiv) and Sc(OTf)_3_ (373 mg, 0.75 mmol, 1.5 equiv) under a nitrogen atmosphere. The reaction mixture was stirred at room temperature for 1–6 h, and then, it was diluted with CH_2_Cl_2_ (40 mL) and washed with NaOH (2 M, 50 mL) and water (2 × 50 mL). The organic layers were dried over anhydrous MgSO_4_, filtered, and concentrated to dryness in a vacuum. The crude product was purified by recrystallization in hexane at −24 °C or by column chromatography to afford the corresponding cinnoline derivatives **3**.

#### Ethyl (3aR*,9bR*)-3,3*a*,5,9*b*-tetrahydro-4*H*-cyclopenta[*c*]cinnoline-4-carboxylate (**3a**)

(111 mg, 91%) as a black solid from aryldiazene carboxylate **2a** (99 mg, 0.5 mmol) was obtained after 1 h reaction, as described in the general procedure. The crude product was purified by flash-column chromatography (SiO_2_, hexanes/AcOEt 98:2) to afford the title compound **3a**. mp: 97–99 °C; IR (neat) *v*_max_ 3058, 2982, 2930, 1694 cm^–1^; ^1^H NMR (400 MHz, CDCl_3_) δ (ppm): 7.07 (dq, *J* = 7.6, 0.6 Hz, 1H), 7.03 (tdd, *J* = 7.6, 1.5, 0.6 Hz, 1H), 6.88 (td, *J* = 7.6, 1.4 Hz, 1H), 6.79 (d, *J* = 7.6 Hz, 1H), 6.33 (bs, 1H), 5.75 (dq, *J* = 6.0, 2.2 Hz, 1H), 5.63 (dq, *J* = 6.0, 2.2 Hz, 1H), 5.35 (q, *J* = 8.7 Hz, 1H), 4.16 (q, *J* = 7.1 Hz, 2H), 4.02 (dq, *J* = 8.7, 2.2 Hz, 1H), 2.83–2.68 (m, 2H), 1.24 (t, *J* = 7.1 Hz, 3H); ^13^C {^1^H} NMR (75 MHz, CDCl_3_) δ (ppm): 155.3, 144.7, 132.5, 129.8, 128.7, 126.6, 126.2, 122.0, 115.2, 62.2, 55.8, 44.7, 36.1, 14.5; ESI-HRMS (CI) *m*/*z*: calcd for C_14_H_17_N_2_O_2_ ([M + H]^+•^), 245.1290; found, 245.1285.

#### Ethyl (3aR*,9bR*)-8-bromo-3,3*a*,5,9*b*-tetrahydro-4*H*-cyclopenta[*c*]cinnoline-4-carboxylate (**3b**)

(125 mg, 77%) as a black solid from aryldiazene carboxylate **2b** (161 mg, 0.5 mmol) was obtained after 1.5 h reaction, as described in the general procedure. The crude product was purified by flash-column chromatography (SiO_2_, hexanes/AcOEt 98:2) to afford the title compound **3b**. mp: 114–116 °C; IR (neat) *v*_max_ 3062, 2964, 2927, 1644 cm^–1^; ^1^H NMR (400 MHz, CDCl_3_) δ (ppm): 7.20 (d, *J* = 2.2 Hz, 1H), 7.13 (dd, *J* = 8.4, 2.2 Hz, 1H), 6.67 (d, *J* = 8.4 Hz, 1H), 6.23 (bs, 1H), 5.76 (dq, *J* = 6.1, 2.4 Hz, 1H), 5.60 (dq, *J* = 6.1, 2.3 Hz, 1H), 5.32 (q, *J* = 6.6 Hz, 1H), 4.15 (q, *J* = 7.1 Hz, 2H, OCH_2_), 3.96 (d, *J* = 8.9 Hz, 1H) 2.76–2.71 (m, 2H), 1.24 (t, *J* = 7.1 Hz, 3H); ^13^C {^1^H} NMR (75 MHz, CDCl_3_) δ (ppm): 155.4, 143.8, 131.8, 131.3, 130.3, 129.0, 128.8, 116.8, 114.0, 62.3, 55.6, 44.5, 35.9, 14.4; ESI-HRMS (CI) *m*/*z*: calcd for C_14_H_16_BrN_2_O_2_ ([M + H]^+•^), 323.0395; found, 323.0399.

#### Ethyl (3aR*,9bR*)-8-methyl-3,3*a*,5,9*b*-tetrahydro-4*H*-cyclopenta[*c*]cinnoline-4-carboxylate (**3c**)

(84 mg, 65%) as a brown solid from aryldiazene carboxylate **2c** (101 mg, 0.5 mmol) was obtained after 1.5 h reaction, as described in the general procedure. The crude product was purified by flash-column chromatography (SiO_2_, hexanes/AcOEt 98:2) to afford the title compound **3c**. mp: 60–62 °C; IR (neat) *v*_max_ 3331, 3054, 2924, 1694 cm^–1^; ^1^H NMR (400 MHz, CDCl_3_) δ (ppm): 6.90 (t, *J* = 1.6 Hz, 1H), 6.84 (dd, *J* = 8.0, 1.6 Hz, 1H), 6.70 (d, *J* = 8.0 Hz, 1H), 6.21 (bs, 1H), 5.74 (dq, *J* = 6.0, 2.3 Hz, 1H), 5.63 (dq, *J* = 6.0, 2.4 Hz, 1H), 5.33 (q, *J* = 5.2 Hz, 1H), 4.15 (q, *J* = 7.1 Hz, 2H), 3.97 (dd, *J* = 9.3, 2.4 Hz, 1H), 2.82–2.64 (m, 2H), 2.24 (s, 3H), 1.24 (t, *J* = 7.1 Hz, 3H); ^13^C {^1^H} NMR (100 MHz, CDCl_3_) δ (ppm): 155.4, 142.2, 132.6, 131.5, 129.7, 129.4, 129.2, 126.8, 115.3, 62.2, 55.7, 44.8, 36.1, 20.7, 14.5; ESI-HRMS (CI) *m*/*z*: calcd for C_15_H_19_N_2_O_2_ ([M + H]^+•^), 259.1447; found, 259.1451.

#### Ethyl (3aR*,9bR*)-8-fluoro-3,3*a*,5,9*b*-tetrahydro-4*H*-cyclopenta[*c*]cinnoline-4-carboxylate (**3d**)

(54 mg, 41%) as a black solid from aryldiazene carboxylate **2d** (109 mg, 0.5 mmol) was obtained after 3 h reaction, as described in the general procedure. The crude product was purified by flash-column chromatography (SiO_2_, hexanes/AcOEt 98:2) to afford the title compound **3d**. mp: 91–94 °C; IR (neat) *v*_max_ 3061, 2925, 1694 cm^–1^; ^1^H NMR (400 MHz, CDCl_3_) δ (ppm): 6.81–6.78 (m, 1H), 6.75–6.72 (m, 2H), 6.15 (bs, 1H), 5.77 (dq, *J* = 6.1, 2.3 Hz, 1H), 5.59 (dq, *J* = 6.1, 2.3 Hz, 1H), 5.31 (q, *J* = 7.2 Hz, 1H), 4.15 (q, *J* = 7.1 Hz, 2H), 3.97 (dt, *J* = 9.2, 2.3 Hz, 1H), 2.76–2.72 (m, 2H), 1.23 (t, *J* = 7.1 Hz, 3H); ^13^C {^1^H} NMR (75 MHz, CDCl_3_) δ (ppm): 158.2 (d, ^1^*J*_CF_ = 240.2 Hz), 156.6, 140.9 (d, ^4^*J*_CF_ = 2.5 Hz), 131.8, 130.4, 128.7, 116.6 (d, ^3^*J*_CF_ = 8.0 Hz), 115.2 (d, ^2^*J*_CF_ = 22.4 Hz), 112.9 (d, ^2^*J*_CF_ = 22.8 Hz), 62.4, 55.4, 45.1, 36.2, 14.5; ^19^F NMR (282 MHz, CDCl_3_) δ (ppm): −121.7. ESI-HRMS (CI) *m*/*z*: calcd for C_14_H_16_FN_2_O_2_ ([M + H]^+•^), 263.1196; found, 263.1198.

#### Ethyl (3aR*,9bR*)-8-(trifluoromethoxy)-3,3*a*,5,9*b*-tetrahydro-4*H*-cyclopenta[*c*]cinnoline-4-carboxylate (**3e**)

(83 mg, 51%) as a black solid from aryldiazene carboxylate **2e** (131 mg, 0.5 mmol) was obtained after 6 h reaction, as described in the general procedure. The crude product was purified by flash-column chromatography (SiO_2_, hexanes/AcOEt 98:2) to afford the title compound **3e**. mp: 69–71 °C; IR (neat) *v*_max_ 3324, 3088, 2985, 2933, 1694 cm^–1^; ^1^H NMR (400 MHz, CDCl_3_) δ (ppm): 6.94 (d, *J* = 2.3 Hz, 1H), 6.91–6.87 (m, 1H), 6.78 (d, *J* = 8.5 Hz, 1H), 6.30 (bs, 1H), 5.77 (dq, *J* = 6.1, 2.3 Hz, 1H), 5.60 (dq, *J* = 6.1, 2.3 Hz, 1H), 5.33 (q, *J* = 7.2 Hz, 1H), 4.16 (q, *J* = 7.1 Hz, 2H), 3.99 (d, *J* = 9.1 Hz, 1H), 2.81–2.68 (m, 2H), 1.24 (t, *J* = 7.1 Hz); ^13^C {^1^H} NMR (75 MHz, CDCl_3_) δ (ppm): 155.4, 143.7 (q, ^3^*J*_CF_ = 2.1 Hz), 143.5, 131.8, 130.5, 128.2, 121.7, 120.5 (q, ^1^*J*_CF_ = 256.1 Hz, CF_3_), 119.3 (C2), 116.1, 62.5, 55.5, 44.8, 36.1, 14.5; ^19^F NMR (282 MHz, CDCl_3_) δ (ppm): −58.6. ESI-HRMS (CI) *m*/*z*: calcd for C_15_H_16_F_3_N_2_O_3_ ([M + H]^+•^), 329.1113; found, 329.1123.

#### Ethyl (3aR*,9bR*)-7-fluoro-3,3a,5,9b-tetrahydro-4*H*-cyclopenta[*c*]cinnoline-4-carboxylate (**3f**) and Ethyl (3aR*,9bR*)-9-fluoro-3,3*a*,5,9*b*-tetrahydro-4*H*-cyclopenta[*c*]cinnoline-4-carboxylate (**3f′**)

(71 mg, 54%) as a brown solid from aryldiazene carboxylate **2f** (117 mg, 0.5 mmol) was obtained after 1 h reaction, as described in the general procedure. The crude product was purified by flash-column chromatography (SiO_2_, hexanes/AcOEt 98:2) to afford the title compound **3f**/**3f**′ as a mixture in a ratio of 64:36. mp: 86–88 °C; IR (neat) *v*_max_ 3067, 2982, 2930, 1682, 1620 cm^–1^; ^1^H NMR (400 MHz, CDCl_3_) δ (ppm): 7.01–6.95 (m, 2H, H9_major_, H7_minor_), 6.61–6.55 (m, 3H, H6_major_, H6_minor_, H8_minor_), 6.51 (d, *J* = 9.5 Hz, 1H, H8_major_), 6.31(bs, 2H, H5_major_, H5_minor_), 5.79–5.74 (m, 2H, H2_major_, H2_minor_), 5.73–5.72 (m, 1H, H1_minor_), 5.61–5.58 (m, 1H, H1_major_), 5.40–5.30 (m, 2H, H3a_major_, H3a_minor_), 4.19–4.13 (m, 4H, OCH_2major_, OCH_2minor_), 4.11 (bs, 1H, H9b)_minor_, 3.97 (d, *J* = 9.5 Hz, 1H, H9b_major_), 2.85–2.79 (m, 2H, H3_minor_), 2.79–2.65 (m, 2H, H3_major_), 1.27–1.23 (m, 6H, CH_3major_, CH_3minor_); ^13^C {^1^H} NMR (100 MHz, CDCl_3_) δ (ppm): 161.1 (d, ^1^*J*_CF_ = 245.43 Hz, C9_minor_), 161.0 (d, ^1^*J*_CF_ = 244.9 Hz, C7_major_), 155.4 (C=O)_minor_, 155.4 (C=O)_major_, 146.1 (d, ^3^*J*_CF_ = 8.2 Hz, C5a_minor_), 146.0 (d, ^3^*J*_CF_ = 9.1 Hz, C5a_major_), 132.4 (C1_major_), 131.0 (d, ^4^*J*_CF_ = 1.9 Hz, C1_minor_), 130.1 (C2_minor_), 130.0 (d, ^3^*J*_CF_ = 9.1 Hz, C9_major_), 129.9 (C2_major_), 127.1 (d, ^3^*J*_CF_ = 9.8 Hz, C7_minor_), 122.3 (C9a_major_), 114.3 (d, ^2^*J*_CF_ = 20.2 Hz, C9a_minor_), 110.6 (d, ^4^*J*_CF_ = 3.0 Hz, C6_minor_), 108.8 (d, ^2^*J*_CF_ = 21.6 Hz, C8_major_), 108.2 (d, ^2^*J*_CF_ = 21.8 Hz, C8_minor_), 102.4 (d, ^2^*J*_CF_ = 24.6 Hz, C6_major_), 62.4 (OCH_2major+minor_), 55.8 (C3a_major_), 55.5 (C3a_minor_), 44.2 (C9b_major_), 39.3 (C9b_minor_), 36.0 (CH_2major_), 35.3 (CH_2minor_), 14.5 (CH_3major+minor_); ^19^F NMR (376 MHz, CDCl_3_) δ (ppm): −115.7_major_, −117.9_minor_; ESI-HRMS (CI) *m*/*z*: calcd for C_14_H_16_FN_2_O_2_ ([M + H]^+•^), 263.1196; found, 263.1171.

#### Ethyl (3aR*,9bR*)-8-(trifluoromethyl)-3,3*a*,5,9*b*-tetrahydro-4*H*-cyclopenta[*c*]cinnoline-4-carboxylate (**3g**)

(38 mg, 24%) as a brown solid from aryldiazene carboxylate **2g** (123 mg, 0.5 mmol) was obtained after 2.5 h reaction, as described in the general procedure. The crude product was purified by flash-column chromatography (Al_2_O_3_, hexanes/AcOEt 95:5) to afford the title compound **3g**. mp: 61–63 °C; IR (neat) *v*_max_ 3064, 2987, 2931, 1682, 1619 cm^–1^; ^1^H NMR (400 MHz, CDCl_3_) δ (ppm): 7.31–7.30 (m, 1H), 7.29–7.26 (m, 1H), 6.83 (d, *J* = 8.2 Hz, 1H), 6.45 (bs, 1H), 5.77 (dq, *J* = 6.0, 1.9 Hz, 1H), 5.63 (dq, *J* = 6.0, 1.9 Hz, 1H), 5.37 (q, *J* = 6.8 Hz, 1H), 4.17 (q, *J* = 7.1 Hz, 2H), 4.03 (dd, *J* = 9.2, 1.9 Hz, 1H), 2.82–2.68 (m, 2H), 1.25 (t, *J* = 7.1 Hz, 3H); ^13^C {^1^H} NMR (100 MHz, CDCl_3_) δ (ppm): 155.3, 147.7, 131.9, 130.5, 126.7, 126.0 (q, ^3^*J*_CF_ = 3.5 Hz), 124.3 (q, ^1^*J*_CF_ = 272.7 Hz), 123.8 (q, ^2^*J*_CF_ = 33.3 Hz), 123.6 (q, ^3^*J*_CF_ = 3.8 Hz), 114.9, 62.6, 55.9, 44.5, 35.9, 14.6; ^19^F NMR (376 MHz, CDCl_3_) δ (ppm): −61.7. ESI-HRMS (CI) *m*/*z*: calcd for C_15_H_16_F_3_N_2_O_2_ ([M + H]^+•^), 313.1164; found, 313.1171.

#### *tert*-Butyl (3aR*,9bR*)-3,3*a*,5,9*b*-tetrahydro-4*H*-cyclopenta[*c*]cinnoline-4-carboxylate (**3h**)

(30 mg, 16%) as a brown oil from aryldiazene carboxylate **2h** (103 mg, 0.5 mmol) was obtained after 1 h reaction, as described in the general procedure. The crude product was purified by flash-column chromatography (Al_2_O_3_, hexanes/AcOEt 97:3) to afford the title compound **3h**. IR (neat) *v*_max_ 3056, 2998, 2925, 2866, 1964 cm^–1^; ^1^H NMR (400 MHz, CDCl_3_) δ (ppm): 7.07 (d, *J* = 7.5 Hz, 1H), 7.04–7.00 (m, 1H), 6.86 (td, *J* = 7.5, 1.2 Hz, 1H), 6.77 (d, *J* = 7.9 Hz, 1H), 6.27 (bs, 1H), 5.74 (dq, *J* = 6.0, 2.2 Hz, 1H), 5.63–5.60 (m, 1H), 5.28 (q, *J* = 5.8 Hz, 1H), 4.00 (dd, *J* = 9.2, 1.8 Hz, 1H), 2.81–2.67 (m, 2H), 1.41 (s, 9H); ^13^C {^1^H} NMR (100 MHz, CDCl_3_) δ (ppm): 154.9, 145.1, 132.6, 129.9, 128.8, 126.8, 126.2, 121.9, 115.1, 81.3, 55.9, 44.8, 36.3, 28.3; ESI-HRMS (CI) *m*/*z*: calcd for C_12_H_13_N_2_O_2_ ([M – ^*t*^Bu + 2H]^+•^), 217.0977; found, 217.0970.

#### Phenyl (3aR*,9bR*)-3,3*a*,5,9*b*-tetrahydro-4*H*-cyclopenta[*c*]cinnoline-4-carboxylate (**3i**)

(106 mg, 73%) as a white powder from aryldiazene carboxylate **2i** (119 mg, 0.5 mmol) was obtained after 1 h reaction, as described in the general procedure. The crude product was purified by recrystallization in hexane at −24 °C to afford the title compound **3i**. mp: 113–116 °C; IR (neat) *v*_max_ 3327, 3061, 2936, 1690 cm^–1^; ^1^H NMR (400 MHz, CDCl_3_) δ (ppm): 7.33 (t, *J* = 7.6 Hz, 2H), 7.19 (tt, *J* = 7.6, 1.1 Hz 1H), 7.13 (d, *J* = 7.5 Hz, 1H), 7.12 (dt, *J* = 7.6, 1.1 Hz, 2H), 6.94 (t, *J* = 7.5 Hz, 1H), 6.88 (d, *J* = 7.7, 1.0 Hz, 1H), 6.48 (bs, 1H), 5.80 (dq, *J* = 6.0, 2.2 Hz, 1H), 5.68 (dq, *J* = 6.0, 2.3 Hz, 1H), 5.50 (qd, *J* = 9.3 Hz, 1.4 Hz, 1H), 4.14 (d, *J* = 9.3 Hz, 1H), 2.93–2.79 (m, 2H); ^13^C {^1^H} NMR (100 MHz, CDCl_3_) δ (ppm): 153.0, 151.0, 144.4, 132.5, 129.8, 129.3, 128.8, 126.5, 126.3, 125.6, 122.5, 121.5, 115.5, 56.4, 45.2, 36.5 (CH_2_); ESI-HRMS (CI) *m*/*z*: calcd for C_18_H_17_N_2_O_2_ ([M + H]^+•^), 293.1290; found, 293.1282.

#### Benzyl (3aR*,9bR*)-3,3*a*,5,9*b*-tetrahydro-4*H*-cyclopenta[*c*]cinnoline-4-carboxylate (**3j**)

(120 mg, 78%) as a white powder from aryldiazene carboxylate **2j** (133 mg, 0.5 mmol) was obtained after 1 h reaction, as described in the general procedure. The crude product was purified by recrystallization in hexane at −24 °C to afford the title compound **3j**. mp: 108–110 °C; IR (neat) *v*_max_ 3296, 3059, 2944, 1694 cm^–1^; ^1^H NMR (400 MHz, CDCl_3_) δ (ppm): 7.36–7.28 (m, 5H), 7.08 (d, *J* = 7.5 Hz, 1H), 7.04 (td, *J* = 7.5, 1.4 Hz, 1H), 6.89 (td, *J* = 7.5, 1.2 Hz, 1H), 6.78 (d, *J* = 7.5 Hz, 1H), 6.35 (bs, 1H), 5.74 (dq, *J* = 6.1, 2.3 Hz, 1H), 5.62 (dq, *J* = 6.1, 2.3 Hz, 1H), 5.37 (bs, 1H), 5.13 (s, 2H), 4.02 (d, *J* = 9.3 Hz, 1H), 2.80 (ddq, *J* = 17.0, 6.4, 2.3 Hz, 1H), 2.75–2.68 (m, 1H); ^13^C {^1^H} NMR (100 MHz, CDCl_3_) δ (ppm): 155.2, 144.7, 136.0, 132.5, 129.7, 128.7, 128.5, 128.1, 127.9, 126.6, 126.3, 122.2, 115.3, 67.9, 56.0, 44.9, 36.2; ESI-HRMS (CI) *m*/*z*: calcd for C_19_H_19_N_2_O_2_ ([M + H]^+•^), 307.1447; found, 307.1439.

#### Allyl (3aR*,9bR*)-3,3*a*,5,9*b*-tetrahydro-4*H*-cyclopenta[*c*]cinnoline-4-carboxylate (**3k**)

(84 mg, 66%) as a brown oil from aryldiazene carboxylate **2k** (102 mg, 0.5 mmol) was obtained after 1 h reaction, as described in the general procedure. The crude product was purified by flash-column chromatography (Al_2_O_3_, hexanes/AcOEt 95:5) to afford the title compound **3k**. IR (neat) *v*_max_ 3059, 3018, 2988, 1964 cm^–1^; ^1^H NMR (400 MHz, CDCl_3_) δ (ppm): 7.08 (d, *J* = 7.5 Hz, 1H), 7.06–7.02 (m, 1H), 6.89 (td, *J* = 7.5, 1.2 Hz, 1H), 6.79 (d, *J* = 7.7 Hz, 1H), 6.34 (bs, 1H), 5.90 (ddt, *J* = 16.8, 10.6, 5.5 Hz, 1H), 5.75 (dq, *J* = 6.0, 2.1 Hz, 1H), 5.64 (dq, *J* = 6.0, 2.1 Hz, 1H), 5.38 (q, *J* = 9.1 Hz, 1H), 5.26 (d, *J* = 16.8 Hz, 1H), 5.24 (d, *J* = 10.6 Hz, 1H), 4.61 (dt, *J* = 5.5, 1.4 Hz, 2H), 4.04 (dd, *J* = 9.1, 2.1 Hz, 1H), 2.81 (ddq, *J* = 17.2, 6.8, 2.3 Hz, 1H), 2.75–2.69 (m, 1H); ^13^C {^1^H} NMR (100 MHz, CDCl_3_) δ (ppm): 154.9, 144.6, 132.5, 132.3, 129.7, 128.7, 126.6, 126.3, 122.1, 117.8, 115.3, 66.7, 55.9, 44.8, 36.1; ESI-HRMS (CI) *m*/*z*: calcd for C_15_H_17_N_2_O_2_ ([M + H]^+•^), 257.1290; found, 257.1264.

#### 2,2,2-Trichloroethyl (3aR*,9bR*)-3,3*a*,5,9*b*-tetrahydro-4*H*-cyclopenta[*c*]cinnoline-4-carboxylate (**3l**)

(115 mg, 66%) as a white powder from aryldiazene carboxylate **2l** (141 mg, 0.5 mmol) was obtained after 1 h reaction, as described in the general procedure. The crude product was purified by recrystallization in hexane at −24 °C to afford the title compound **3l**. mp: 91–93 °C; IR (neat) *v*_max_ 3315, 3063, 3004, 2956, 2916, 1694 cm^–1^; ^1^H NMR (400 MHz, CDCl_3_) δ (ppm): 7.09 (d, *J* = 7.6 Hz, 1H), 7.05 (t, *J* = 7.6 Hz, 1H), 6.91 (td, *J* = 7.6, 1.0 Hz, 1H), 6.84–6.80 (m, 1H), 6.39 (bs, 1H) 5.77 (dq, *J* = 5.9, 2.2 Hz, 1H), 5.64–5.62 (m, 1H), 5.41 (q, *J* = 8.6 Hz, 1H), 4.81 (d, *J* = 10.8 Hz, 1H), 4.72 (d, *J* = 10.8 Hz, 1H), 4.08 (d, *J* = 8.6 Hz, 1H), 2.88–2.75 (m, 2H, CH_2_); ^13^C {^1^H} NMR (100 MHz, CDCl_3_) δ (ppm): 153.1, 144.3, 132.4, 129.7, 129.0, 128.7, 126.5, 122.5, 115.5, 95.2, 75.4, 56.5, 45.2, 36.4; ESI-HRMS (CI) *m*/*z*: calcd for C_14_H_14_Cl_3_N_2_O_2_ ([M + H]^+•^), 347.0121; found, 347.0111.

#### (3aR*,9bR*)-1-(3,3*a*,5,9*b*-Tetrahydro-4*H*-cyclopenta[*c*]cinnolin-4-yl)ethan-1-one (**3m**) and (3aR*,9bR*)-1-(1,3*a*,5,9*b*-Tetrahydro-4*H*-cyclopenta[*c*]cinnolin-4-yl)ethan-1-one (**3m′**)

(99 mg, 92%) as a brown solid from *N*-acetyl aryldiazene **2m** (74 mg, 0.5 mmol) was obtained after 1.5 h reaction, as described in the general procedure. The crude product was purified by recrystallization in hexane at −24 °C to afford the title compound **3m**/**3m**′ as a mixture of regioisomers in a ratio of 59:41. mp: 57–60 °C; IR (neat) *v*_max_ 3067, 2923, 2850, 1634 cm^–1^; ^1^H NMR (400 MHz, CDCl_3_) δ (ppm): 7.70 (bs, 1H, H5_major_), 7.12–7.10 (m, 2H, H7_minor_, H9_minor_), 7.08–7.01 (m, 3H, H7_major_, H9_major_, H8_minor_), 6.95 (d, *J* = 7.9 Hz, 1H, H6_minor_), 6.86 (td, *J* = 7.5, 1.2 Hz, 1H, H8_major_), 6.80 (dd, *J* = 7.9, 1.2 Hz, 1H, H6_major_), 5.77–5.71 (m, 4H, H2_major_, H2_minor_, H3_minor_, H3a_minor_), 5.68 (dq, *J* = 6.0, 2.4 Hz, 1H, H1_major_), 5.51 (bs, 1H, H5_minor_), 5.04 (qd, *J* = 9.2, 1.4 Hz, 1H, H3a_major_), 4.09 (dd, *J* = 9.2, 2.1 Hz, 1H, H9b_major_), 4.00 (d, *J* = 9.2 Hz, 1H, H9b_minor_), 2.84 (ddq, *J* = 16.8, 6.5, 2.4 Hz, 1H, H3_major_), 2.75–2.65 (m, 2H, H3_major_, H1_minor_), 2.46–2.39 (m, 1H, H1_minor_), 2.20 (s, 3H, CH_3minor_), 2.17 (s, 3H, CH_3major_); ^13^C {^1^H} NMR (100 MHz, CDCl_3_) δ (ppm): 172.9 (C=O)_minor_, 163.4 (C=O)_major_, 143.8 (C5a_major_), 141.6 (C5a_minor_), 133.1 (C3)_minor_, 132.5 (C1_major_), 130.4 (C9a_minor_), 129.8 (C2_minor_), 129.3 (C2_major_), 128.8 (C9_minor_), 128.4 (C7_major_), 126.7 (C9_major_), 126.6 (C7_minor_), 125.4 (C9a_major_), 124.9 (C8_minor_), 121.9 (C8_major_), 121.1 (C6_minor_), 115.1 (C6_major_), 57.3 (C3a_major_), 52.2 (C3a_minor_), 45.4 (C9b_major_), 44.2 (C9b_minor_), 36.5 (C3_major_), 34.7 (C1_minor_), 21.5 (CH_3minor_), 20.2 (CH_3major_); ESI-HRMS (CI) *m*/*z*: calcd for C_13_H_15_N_2_O ([M + H]^+•^), 215.1184; found, 215.1184.

#### (3aR*,9bR*)-1-(8-(Trifluoromethyl)-3,3a,5,9b-tetrahydro-4*H*-cyclopenta[*c*]cinnolin-4-yl)ethan-1-one (**3n**) and (3aR*,9bR*)-1-(8-(Ttrifluoromethyl)-1,3a,5,9b-tetrahydro-4*H*-cyclopenta[*c*]cinnolin-4-yl)ethan-1-one (**3n′**)

(125 mg, 63%) as a white powder from *N*-acetyl aryldiazene **2n** (152 mg, 0.7 mmol), cyclopentadiene (73 μL, 0.87 mmol), and Sc(OTf)_3_ (378 mg, 0.77 mmol) was obtained after 1.5 h reaction, as described in the general procedure. The crude product was purified by recrystallization in hexane at −24 °C to afford the title compound **3n**/**3n**′ as a mixture of regioisomers in a ratio of 74:26. mp: 128–134 °C; IR (neat) *v*_max_ 3060, 3058, 2920, 2850, 1634 cm^–1^; ^1^H NMR (400 MHz, CDCl_3_) δ (ppm): 7.87 (bs, 1H, H5_major_), 7.36–7.34 (m, 2H, H7_minor_, H9_minor_), 7.31–7.28 (m, 2H, H7_major_, H9_major_), 7.01 (d, *J* = 8.6 Hz, 1H, H6_minor_), 6.86 (d, *J* = 8.1 Hz, 1H, H6_major_), 5.82–5.75 (m, 3H, H2_major_, H2_minor_, H3_minor_), 5.71 (dq, d, *J* = 6.0, 2.3 Hz 1H, H1_major_), 5.65 (bs, 2H, H3a_minor_, H5_minor_), 5.09 (tdd, *J* = 8.9, 6.8, 1.7 Hz, 1H, H3a_major_), 4.11 (dd, *J* = 8.9, 2.3 Hz, 1H, H9b_major_), 4.02 (d, *J* = 8.9 Hz, 1H, H9b_minor_), 2.83 (ddq, *J* = 16.9, 6.8, 2.3 Hz, 1H, C3_major_), 2.72 (ddt, *J* = 16.9, 8.9, 2.3 Hz, 1H, C3)_major_, 2.44 (dd, *J* = 7.8, 2.1 Hz, 1H, C1_mino_)_r_, 2.40 (dd, *J* = 7.8, 1.8 Hz, 1H C1_minor_), 2.20 (s, 6H, CH_3major_, CH_3minor_); ^13^C {^1^H} NMR (100 MHz, CDCl_3_) δ (ppm): 172.8 (C=O)_minor_, 163.6 (C=O)_major_, 146.4 (q, ^5^*J*_CF_ = 1.1 Hz, C5a_major_), 144.4 (C5a_minor_), 132.4 (C3_minor_), 131.8 (C1_major_), 130.3 (C2_minor_), 130.0 (C2_major_), 129.7 (C9a_minor_), 126.2 (q, ^3^*J*_CF_ = 3.9 Hz, C9_minor_), 126.2 (q, ^2^*J*_CF_ = 32.3 Hz, C8_minor_), 125.6 (q, ^3^*J*_CF_ = 3.8 Hz, C9_major_), 125.3 (C9a_major_), 124.3 (q, ^1^*J*_CF_ = 271.7 Hz, CF_3major_), 124.1 (q, ^1^*J*_CF_ = 272.7 Hz, CF_3minor_), 123.8 (q, ^3^*J*_CF_ = 3.8 Hz, C7_major_), 123.6 (q, ^3^*J*_CF_ = 3.7 Hz, C7_minor_), 123.6 (q, ^2^*J*_CF_ = 32.3 Hz, C8_major_), 120.0 (C6_minor_), 114.8 (C6_major_), 57.2 (C3a_major_), 52.0 (C3a_minor_), 45.0 (C9b_major_), 43.7 (C9b_minor_), 36.1 (C3_major_), 33.7 (C1_minor_), 21.5 (CH_3minor_), 20.0 (CH_3major_); ^19^F NMR (376 MHz, CDCl3) δ (ppm): −61.7_major_, −62.0_minor_; ESI-HRMS (CI) *m*/*z*: calcd for C_14_H_14_F_3_N_2_O ([M + H]^+•^), 283.1058; found, 283.1033.

#### Phenyl((3aR*,9bR*)-3,3*a*,5,9*b*-tetrahydro-4*H*-cyclopenta[*c*]cinnolin-4-yl)methanone (**3o**)

(138 mg, >99%) as a brown solid from *N*-benzoyl aryldiazene **2o** (108 mg, 0.5 mmol) was obtained after 1 h reaction, as described in the general procedure. The crude product was purified by recrystallization in hexane at −24 °C to afford the title compound **3o**. mp: 53–56 °C; IR (neat) *v*_max_ 3286, 3057, 2922, 1628 cm^–1^; ^1^H NMR (400 MHz, CDCl_3_) δ (ppm): 7.71 (bs, 1H), 7.48–7.41 (m, 5H), 7.11–7.06 (m, 2H), 6.92–6.89 (m, 2H), 5.72–5.66 (m, 2H), 5.05–4.99 (m, 1H), 3.97 (d, *J* = 8.3 Hz, 1H), 3.02 (d, *J* = 11.9 Hz, 1H), 2.67–2.61 (m, 1H); ^13^C {^1^H} NMR (100 MHz, CDCl_3_) δ (ppm): 165.2, 143.8, 134.4, 132.5, 130.2, 129.3, 128.6, 128.5, 127.1, 126.7, 125.6, 122.0, 115.3, 58.3, 45.1, 36.6; ESI-HRMS (CI) *m*/*z*: calcd for C_18_H_17_N_2_O ([M + H]^+•^), 277.1341; found, 277.1336.

#### Gram Scale Procedure of Cinnoline **3a**

To a stirred solution of aryldiazene carboxylate **2a** (535 mg, 3 mmol) in CHCl_3_ (9 mL), was added cyclopentadiene (0.38 mL, 4.5 mmol, 1.5 equiv) and Sc(OTf)_3_ (2.237 g, 4.5 mmol, 1.5 equiv) under a nitrogen atmosphere. The reaction mixture was stirred at room temperature for 1–6 h, and then, it was diluted with CH_2_Cl_2_ (40 mL) and washed with NaOH (2 M, 50 mL) and water (2 × 50 mL). The organic layers were dried over anhydrous MgSO_4_, filtered, and concentrated to dryness in vacuum. The crude product was purified by column chromatography to afford the desired cinnoline **3a** (523 mg, 71% yield).

### Synthetic Transformations

#### Synthesis of Compound **3p**

A stirred solution of **3a** (0.2 mmol, 49 mg) in MeOH (3 mL) was refluxed using a heating mantle for 48 h under a nitrogen atmosphere. Then, the crude reaction was diluted with H_2_O (25 mL), and the product was extracted with CH_2_Cl_2_ (2 × 25 mL). The organic layers were dried over anhydrous MgSO_4_, filtered, and concentrated to dryness in a vacuum. The crude product was purified by flash-column chromatography (SiO_2_, hexanes/AcOEt 97:3) to afford the title compound **3p**.

##### Methyl (3aR*,9bR*)-3,3*a*,5,9*b*-tetrahydro-4*H*-cyclopenta[*c*]cinnoline-4-carboxylate (**3p**)

Brown solid, 34 mg, 73%; mp: 86–89 °C; IR (neat) *v*_max_ 3058, 2956, 2924, 1694 cm^–1^; ^1^H NMR (400 MHz, CDCl_3_) δ (ppm): 7.07 (dt, *J* = 7.5, 0.7 Hz, 1H), 7.06–7.01 (m, 1H), 6.89 (td, *J* = 7.5, 1.2 Hz, 1H), 6.79 (d, *J* = 7.9 Hz, 1H), 6.16 (bs, 1H), 5.74 (dq, *J* = 6.0, 2.3 Hz, 1H), 5.62 (dq, *J* = 6.0, 2.3 Hz, 1H), 5.33 (s, 1H), 4.01 (d, *J* = 9.2 Hz, 1H), 3.72 (s, 3H), 2.82–2.67 (m, 2H); ^13^C {^1^H} NMR (100 MHz, CDCl_3_) δ (ppm): 155.7, 144.6, 132.5, 129.8, 128.8, 126.7, 126.4, 122.3, 115.4, 55.9, 53.3, 44.8, 36.2; ESI-HRMS (CI) *m*/*z*: calcd for C_13_H_15_N_2_O_2_ ([M + H]^+•^), 231.1134; found, 231.1126.

#### Synthesis of Compound **4**

To a stirred solution of **3a** (0.2 mmol, 49 mg) in MeOH (10 mL), Ni-Raney (∼50 mg, washed with MeOH) was added, and the reaction mixture was stirred under a hydrogen atmosphere (2 bar) for 22 h. Then, the crude product was filtered through a pad of Celite to obtain the pure title compound **4**.

##### Ethyl (3aR*,9bR*)-1,2,3,3*a*,5,9*b*-hexahydro-4*H*-cyclopenta[*c*]cinnoline-4-carboxylate (**4**)

Brown oil, 48 mg, 97%; IR (neat) *v*_max_ 3327, 3061, 2956, 2867, 1694 cm^–1^; ^1^H NMR (400 MHz, CDCl_3_) δ (ppm): 7.08 (d, *J* = 7.5 Hz, 1H), 7.03 (t, *J* = 7.3 Hz, 1H), 6.88 (t, *J* = 7.4, Hz, 1H), 6.78 (d, *J* = 7.8 Hz, 1H), 6.27 (bs, 1H), 4.87 (q, *J* = 8.1 Hz, 1H), 4.14 (q, *J* = 7.1 Hz, 2H), 3.26 (q, *J* = 8.1 Hz, 1H), 2.10–1.97 (m, 2H), 1.95–1.88 (m, 1H), 1.74–1.65 (m, 1H), 1.64–1.57 (m, 1H), 1.53–1.44 (m, 1H), 1.24 (t, *J* = 7.1 Hz, 3H); ^13^C {^1^H} NMR (75 MHz, CDCl_3_) δ (ppm): 155.0, 144.5, 128.9, 128.7, 125.9, 121.8, 114.7, 62.0, 57.1, 38.2, 34.8, 29.2, 23.7, 14.5; ESI-HRMS (CI) *m*/*z*: calcd for C_14_H_19_N_2_O_2_ ([M + H]^+•^), 247.1447; found, 247.1437.

## Supporting Information Available

General experimental procedures; characterization data; NMR and ESI-HRMS spectra of **1e**, **2e**, **2n**, **3**, and **4**; and crystallographic structure of **3a** (PDF)

## Notes

CCDC 2164540 contains the supplementary crystallographic data for this paper. These data can be obtained free of charge via www.ccdc.cam.ac.uk/data_request/cif, or by emailing data_request@ccdc.cam.ac.uk, or by contacting The Cambridge Crystallographic Data Centre, 12 Union Road, Cambridge CB2 1EZ, UK; fax: +44 1223 336033.
