# Supplementary material for: Sc(OTf)3-Mediated [4 + 2] Annulations of N-Carbonyl Aryldiazenes with Cyclopentadiene to Construct Cinnoline Derivatives: Azo-Povarov Reaction
Source: J Org Chem. 2022 Aug 16;87(17):11583–92. doi: 10.1021/acs.joc.2c01224 (PMC9447289; doi:10.1021/acs.joc.2c01224)
Supplement: Supplementary file 1 — jo2c01224_si_001.pdf [file jo2c01224_si_001.pdf]

Supporting Information

$Sc(OTf)_3$-Mediated [4 + 2] Annulations of *N*-Carbonyl Aryldiazenes with Cyclopentadiene to Construct Cinnoline Derivatives: azo-Povarov Reaction

Xabier Jiménez-Aberásturi, Francisco Palacios, Jesús M. de los Santos*

Department of Organic Chemistry I, Faculty of Pharmacy and Lascaray Research Center, University of the Basque Country (UPV/EHU), Paseo de la Universidad 7, 01006 Vitoria, Spain.

**Corresponding author E-mail: jesus.delossantos@ehu.eus*

Table of contents

1. ^{1}H NMR, ^{13}C NMR and ^{19}F NMR spectra of compounds……………………………………..…… S2
2. ORTEP view and X-ray crystallographic statistics for compound **3a**………………..…...…...S30
3. References……………………………………………………………………………...S32

1. ¹H NMR and ¹³C NMR spectra of compounds

¹H NMR (400 MHz, $CDCl_3$) of functionalized hydrazine **1e**

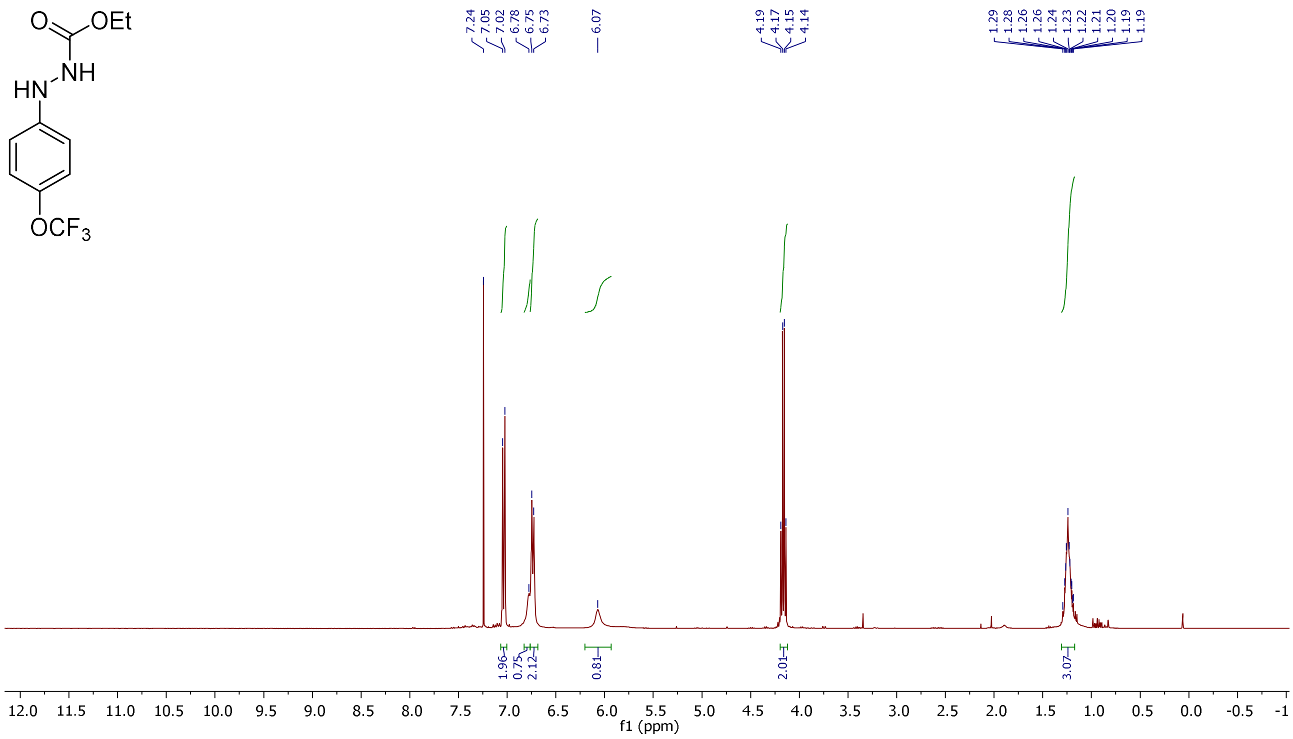

¹³C {¹H} NMR (100 MHz, $CDCl_3$) of functionalized hydrazine **1e**

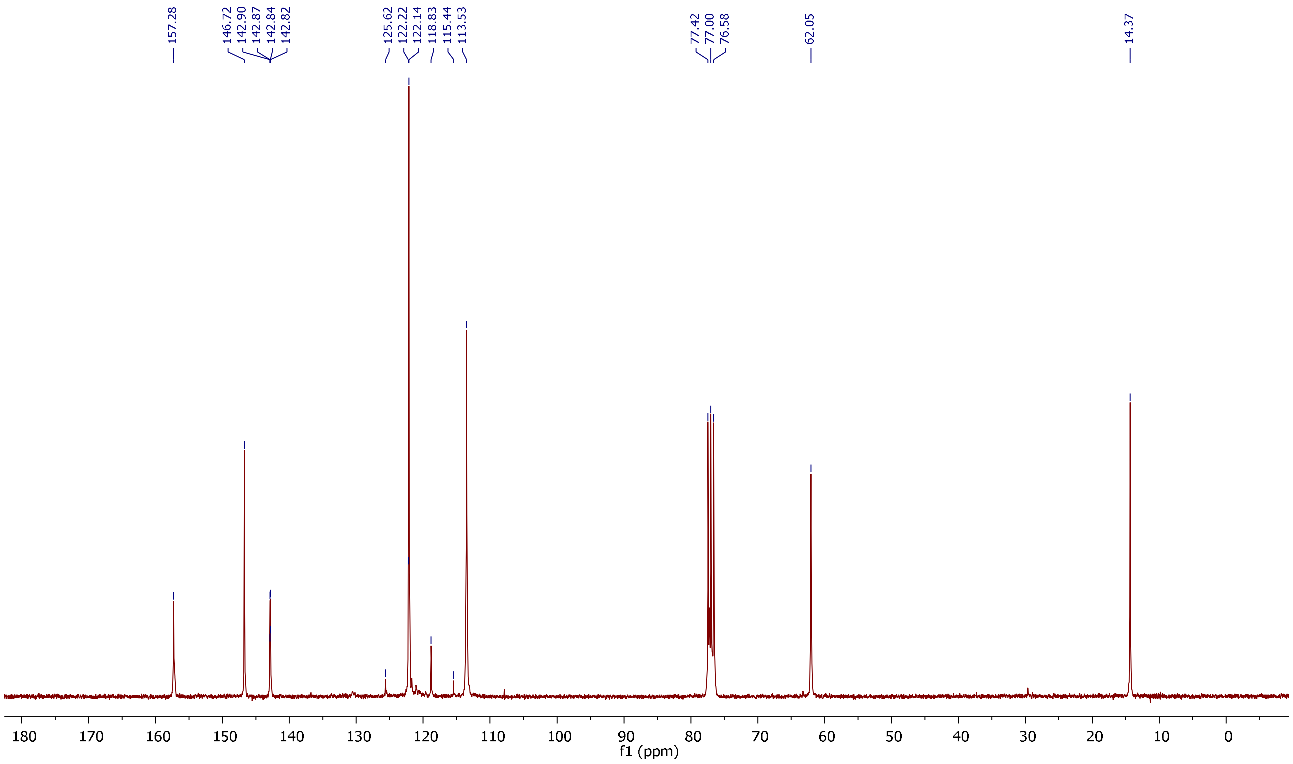

^{19}F (282 MHz, $CDCl_3$) of functionalized hydrazine **1e**

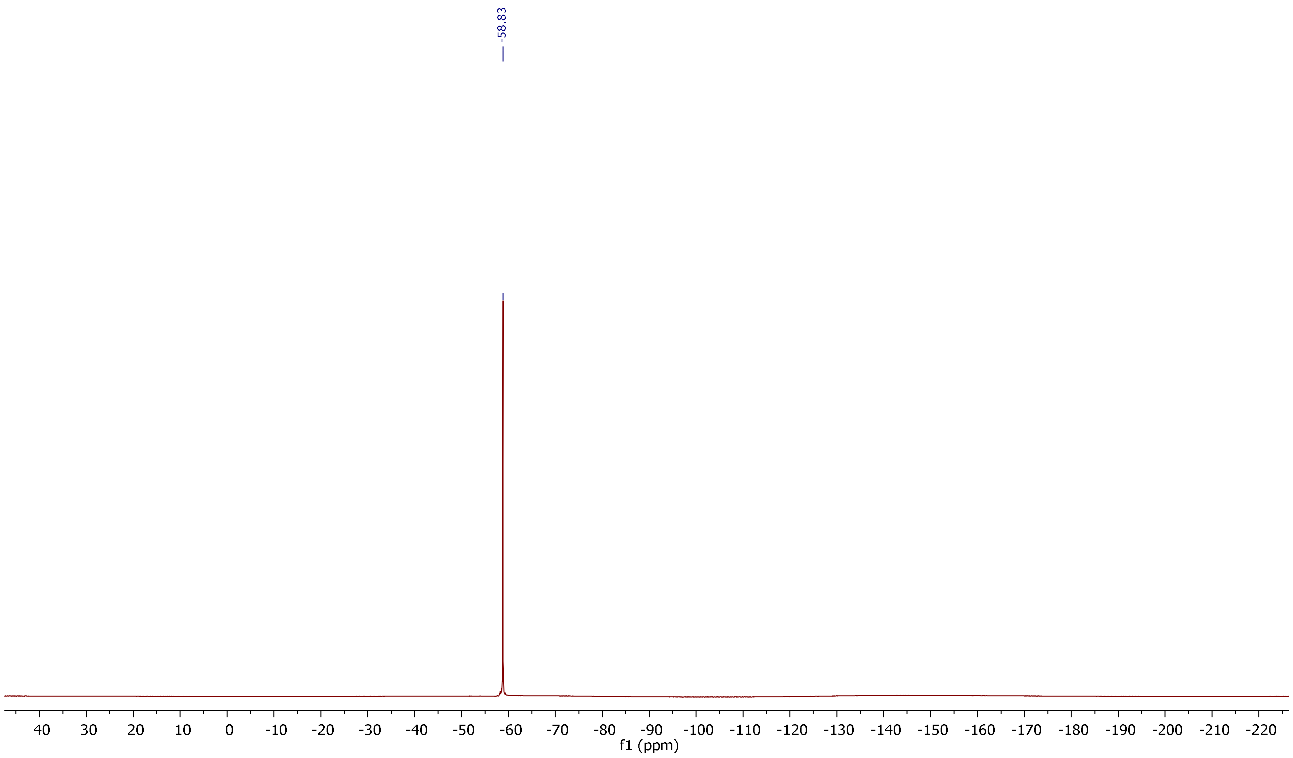

^{1}H NMR (400 MHz, $CDCl_3$) of aryldiazene carboxylate **2e**

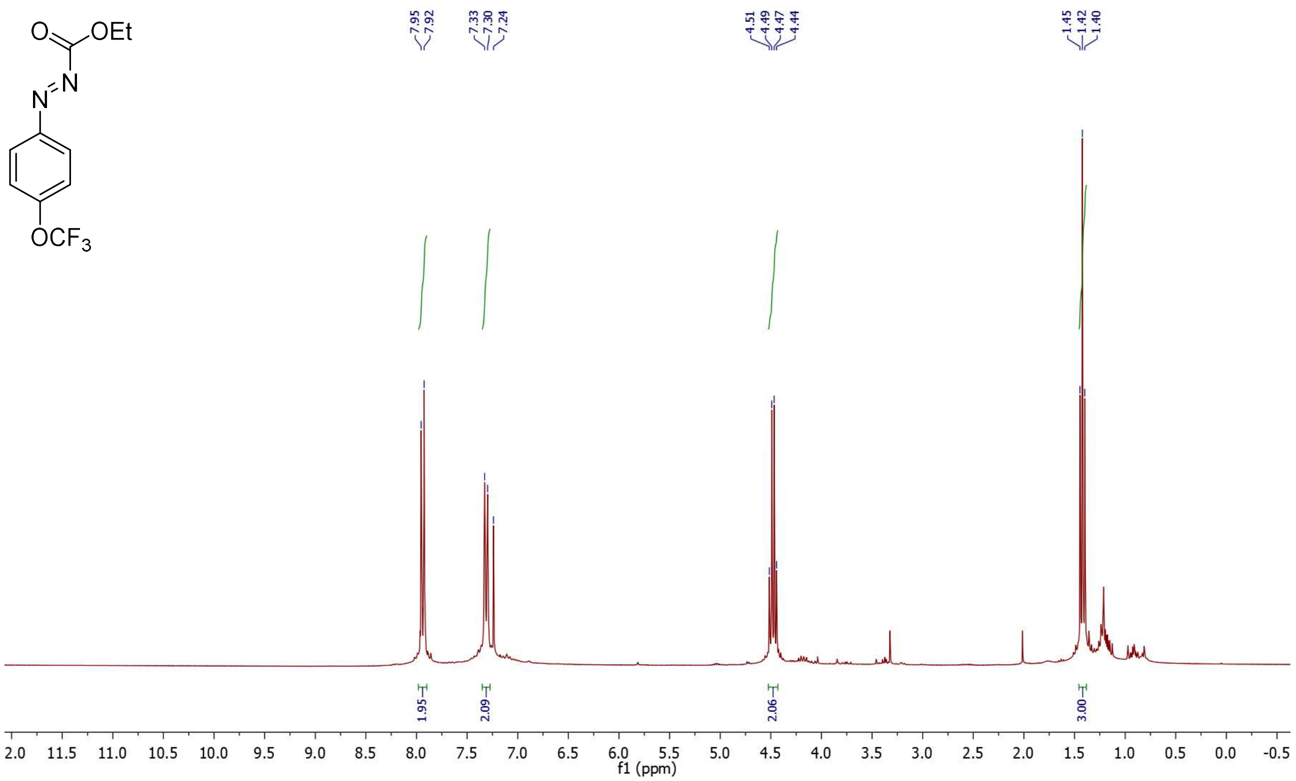

^{13}C {^{1}H} NMR (100 MHz, $CDCl_3$) of aryldiazene carboxylate **2e**

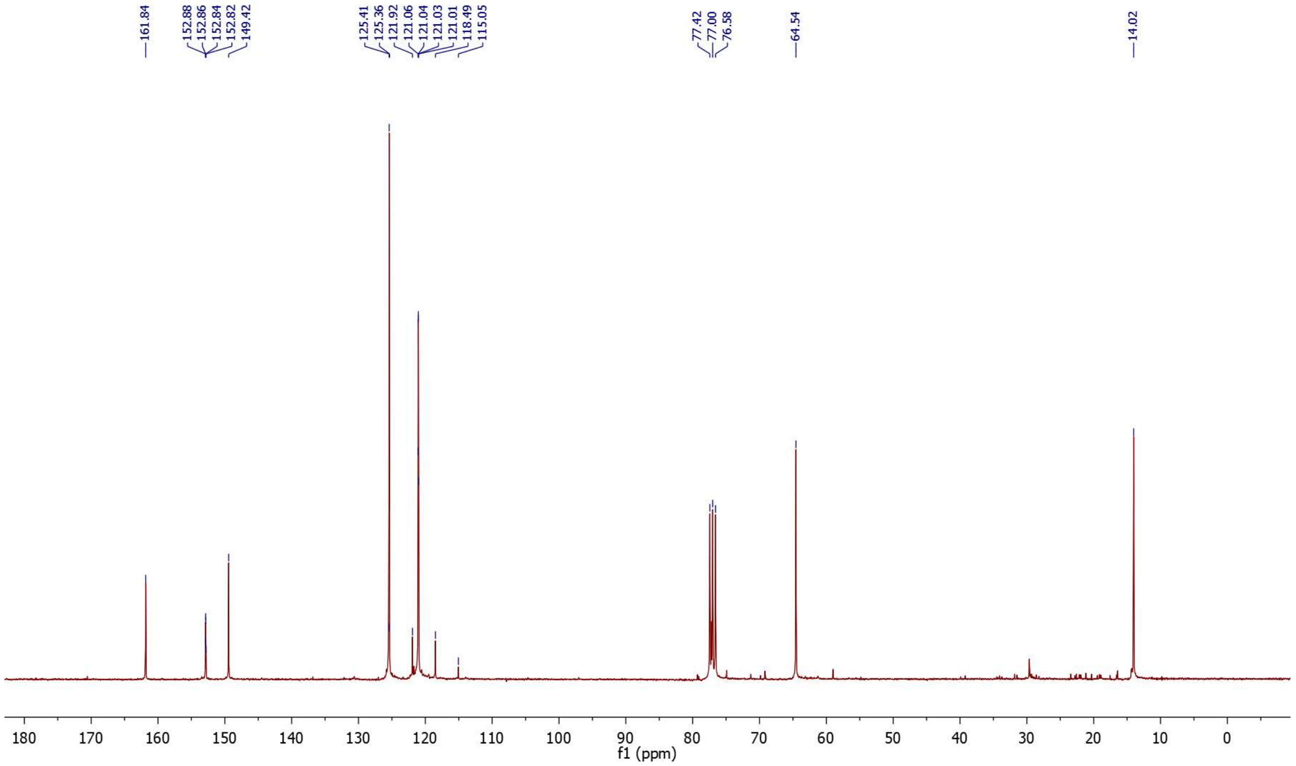

^{19}F (282 MHz, $CDCl_3$) of aryldiazene carboxylate **2e**

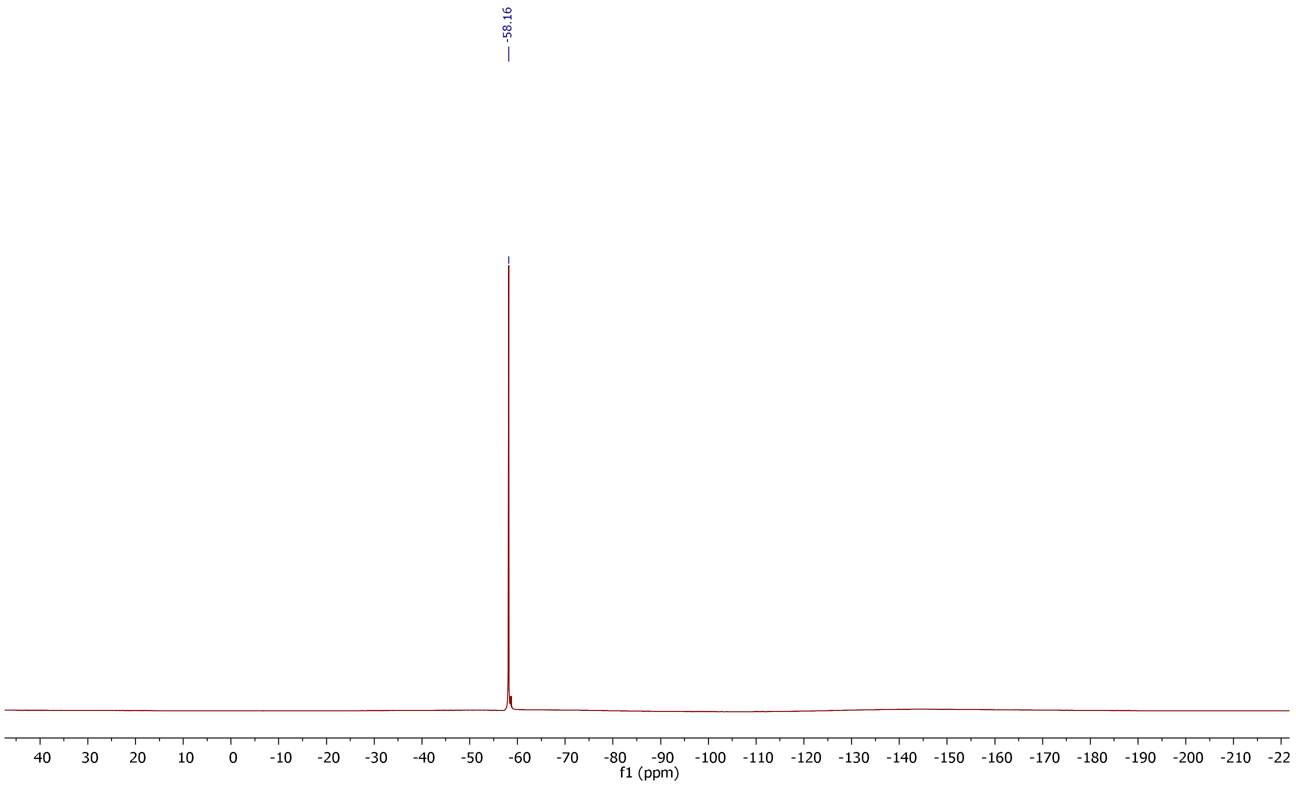

^{1}H NMR (400 MHz, $CDCl_3$) of *N*-acetyl aryldiazene **2n**

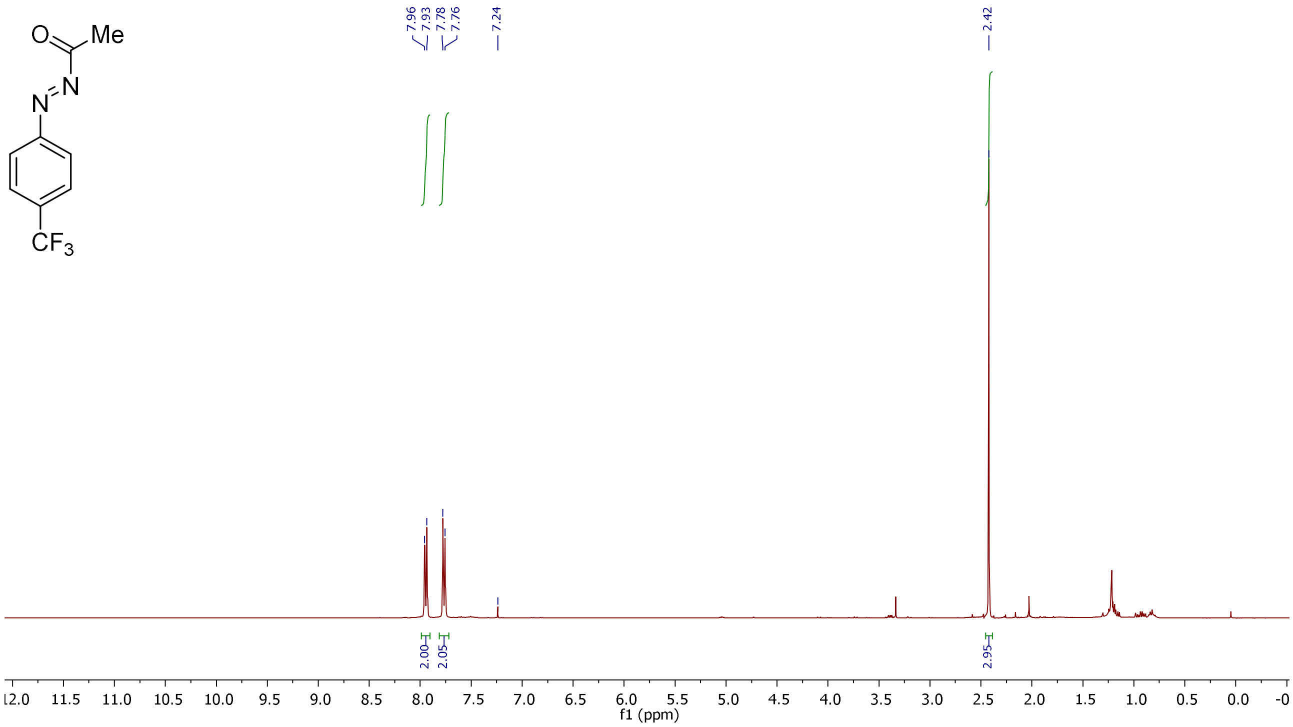

^{13}C {^{1}H} NMR (100 MHz, $CDCl_3$) of *N*-acetyl aryldiazene **2n**

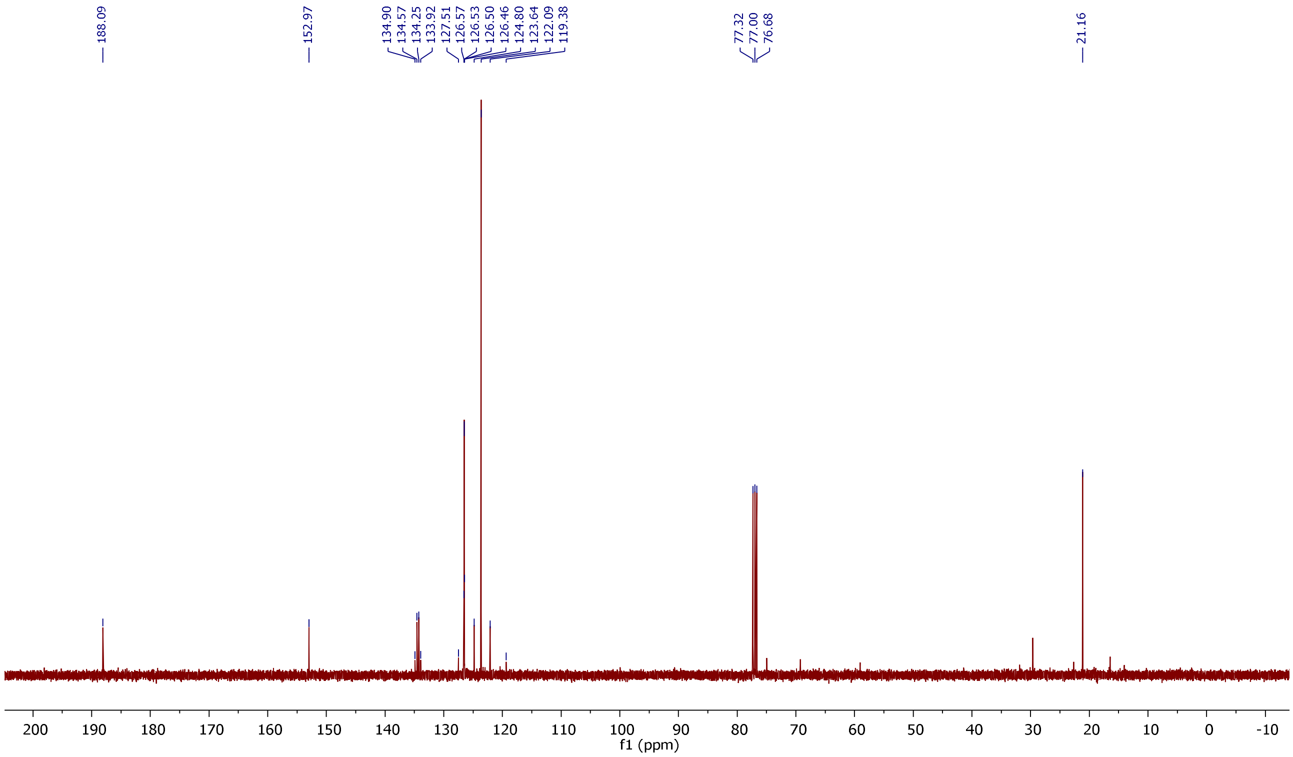

^{19}F (376 MHz, $CDCl_3$) of *N*-acetyl aryldiazene **2n**

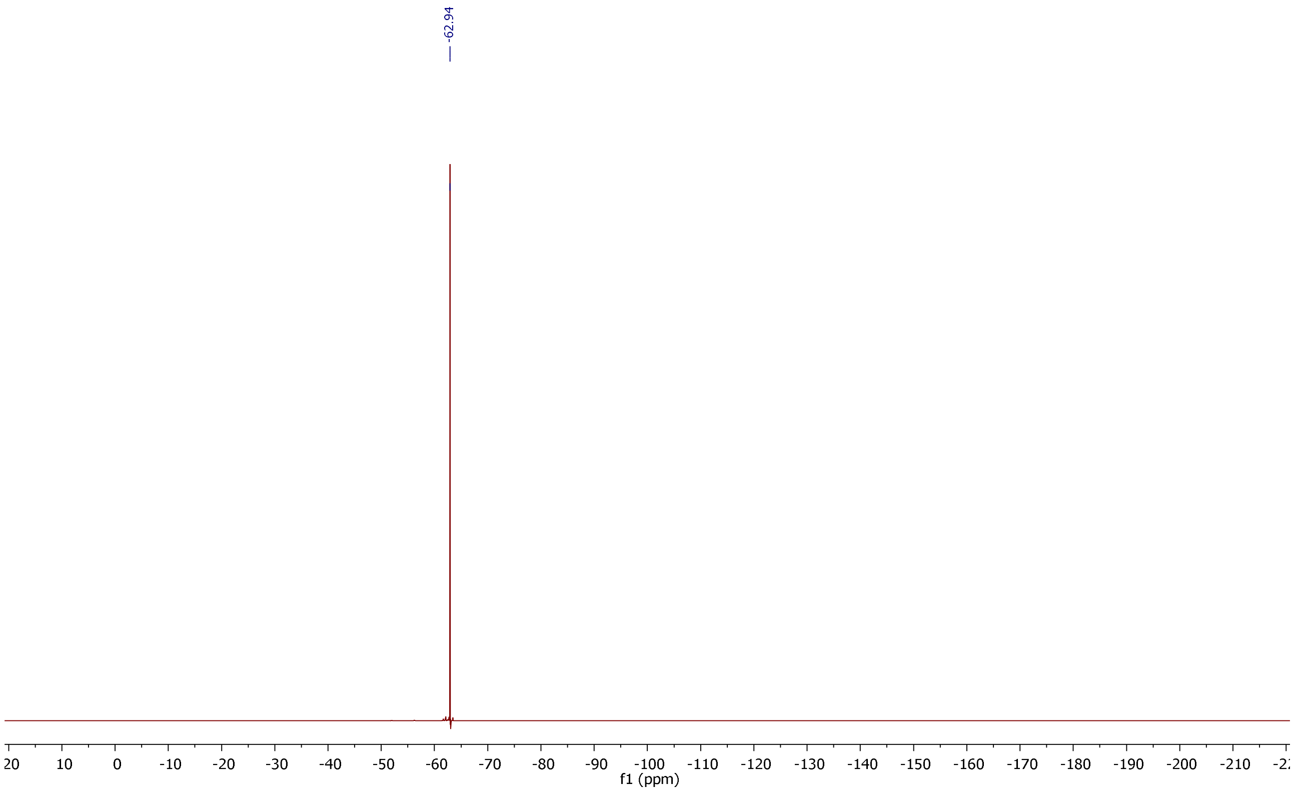

^{1}H NMR (400 MHz, $CDCl_3$) of cinnoline derivative **3a**

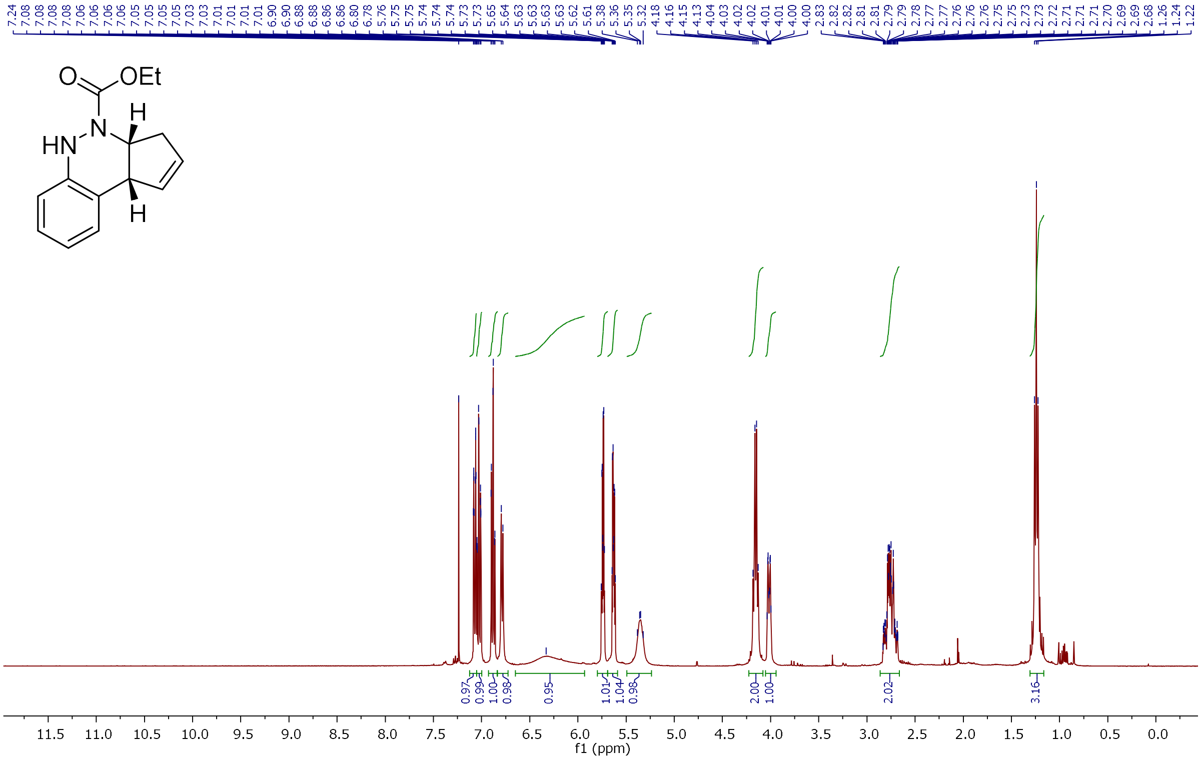

^{13}C {^{1}H} NMR (100 MHz, $CDCl_3$) of cinnoline derivative **3a**

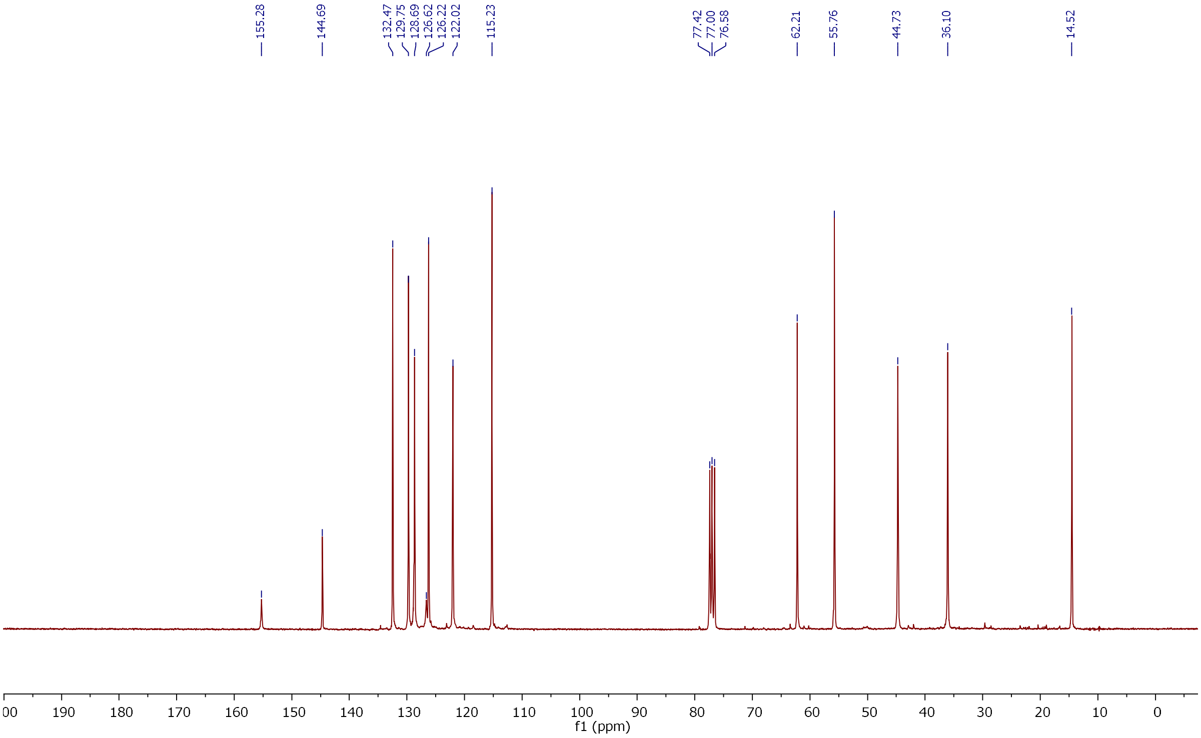

^{1}H NMR (400 MHz, $CDCl_3$) of cinnoline derivative **3b**

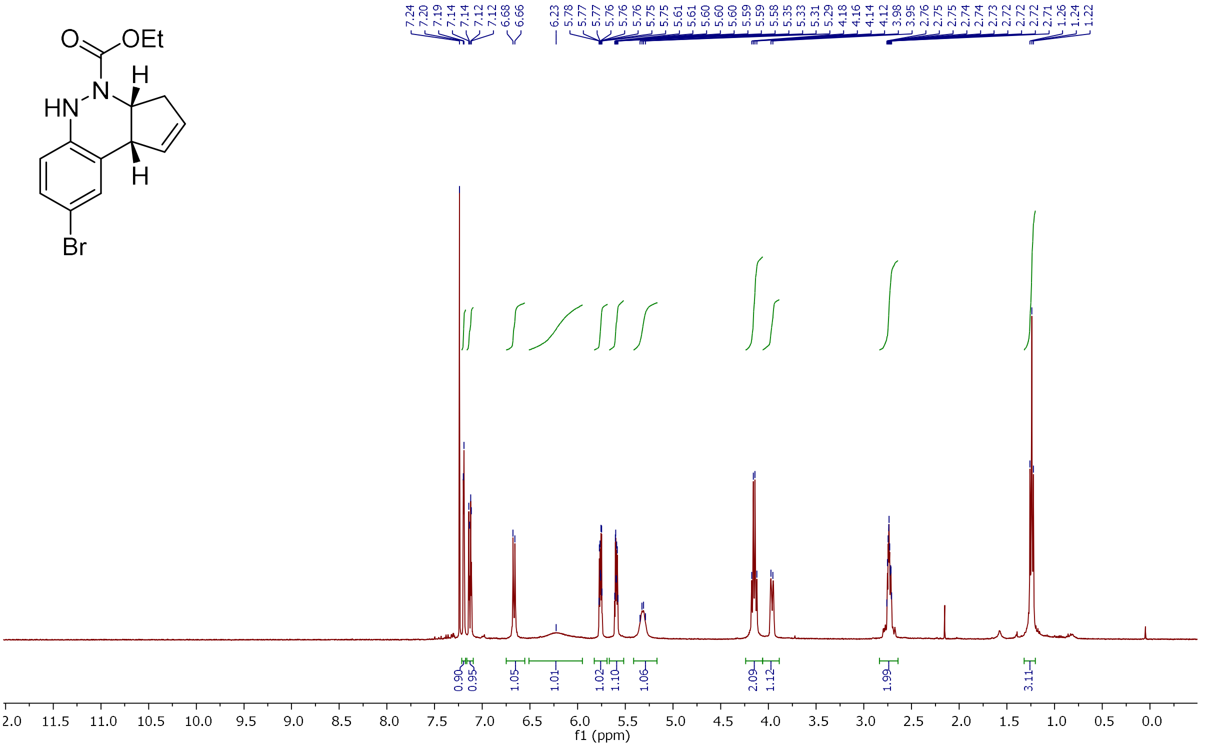

^{13}C {^{1}H} NMR (100 MHz, $CDCl_3$) of cinnoline derivative **3b**

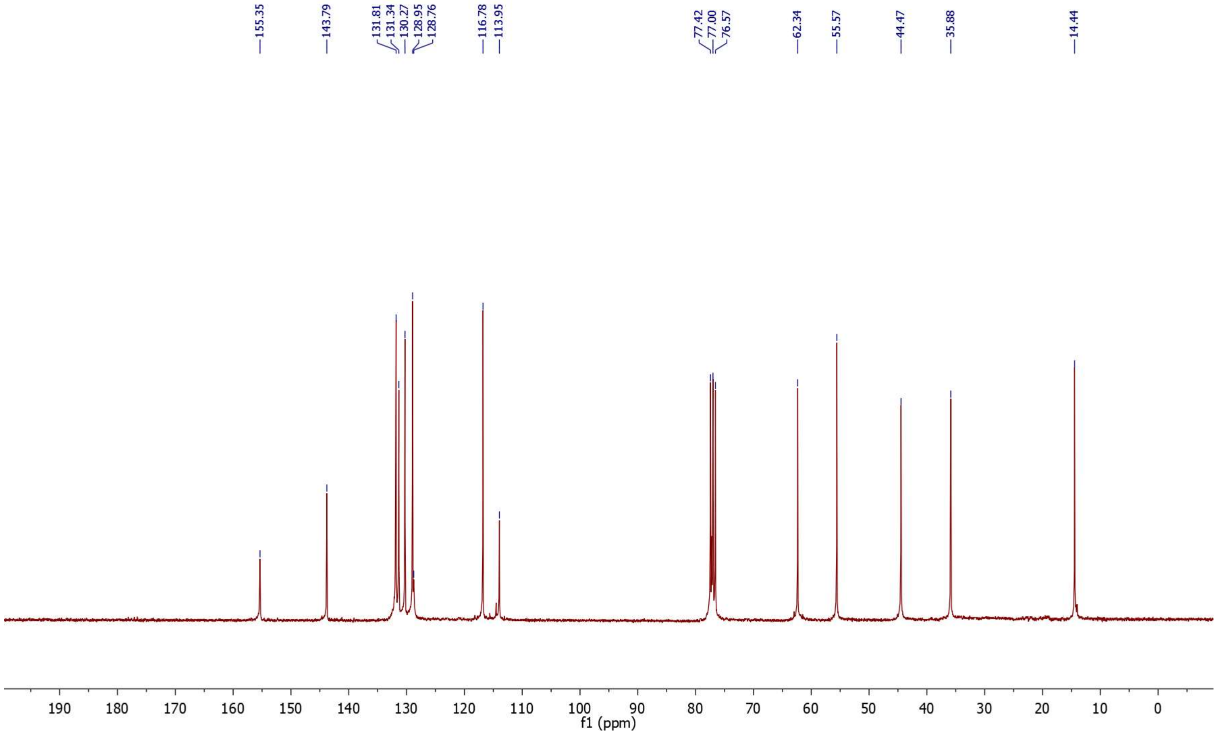

^{1}H NMR (400 MHz, $CDCl_3$) of cinnoline derivative **3c**

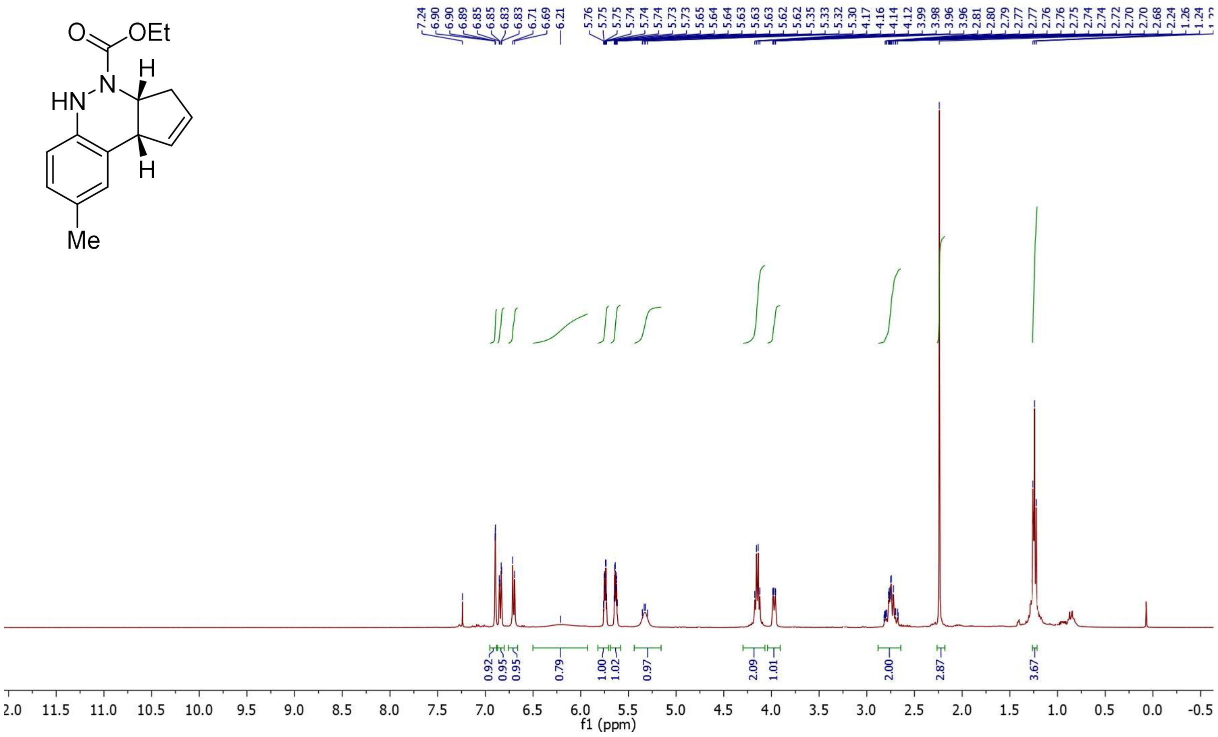

^{13}C {^{1}H} NMR (100 MHz, $CDCl_3$) of cinnoline derivative **3c**

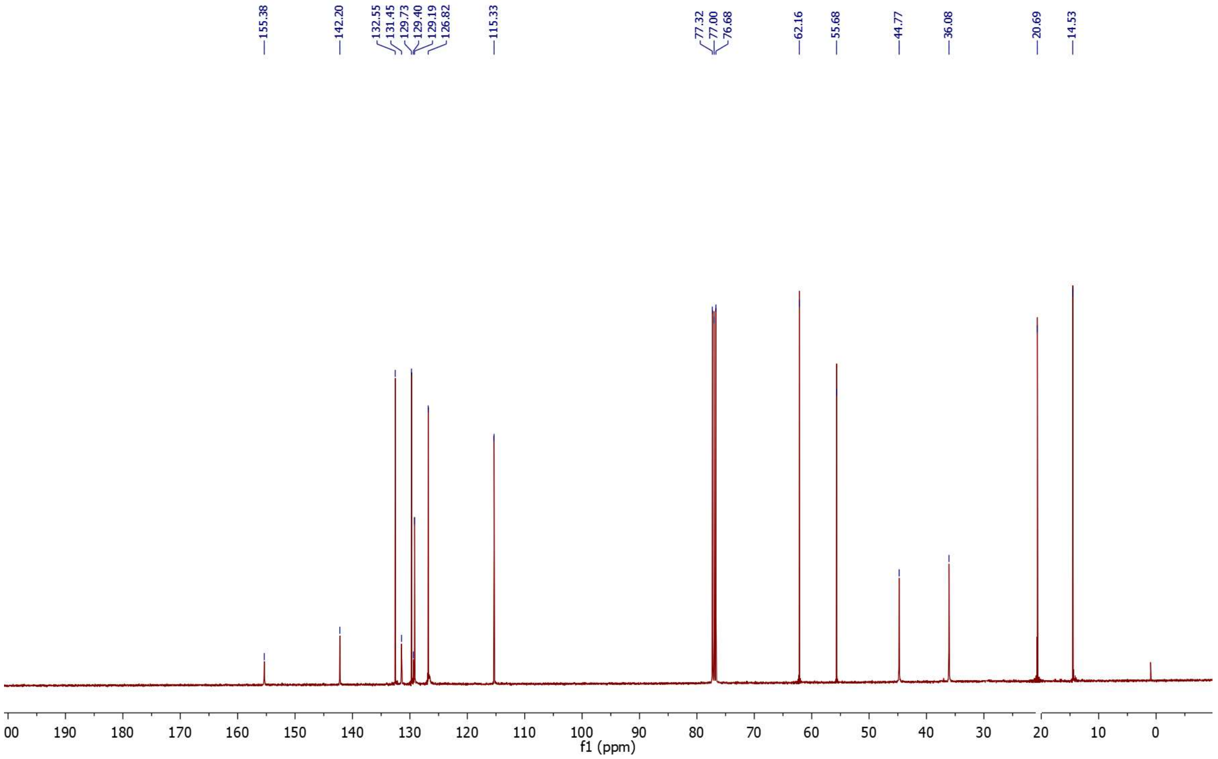

^{1}H NMR (400 MHz, $CDCl_3$) of cinnoline derivative **3d**

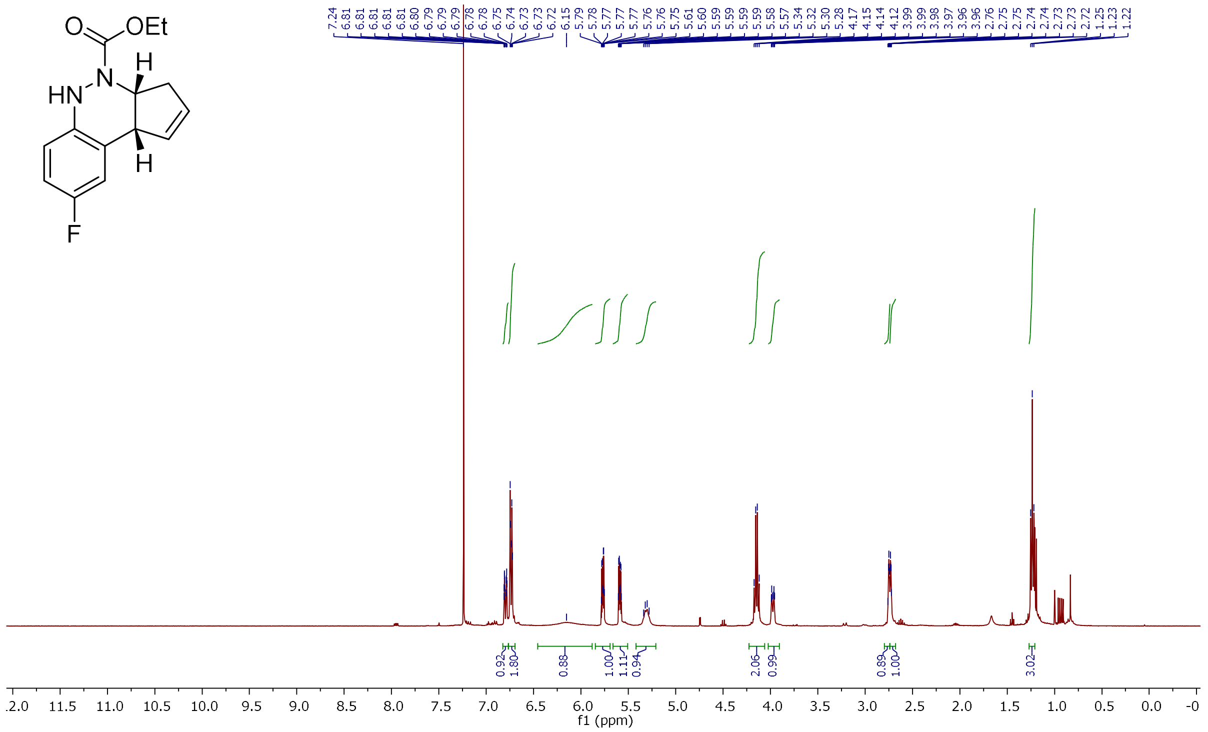

^{13}C {^{1}H} NMR (100 MHz, $CDCl_3$) of cinnoline derivative **3d**

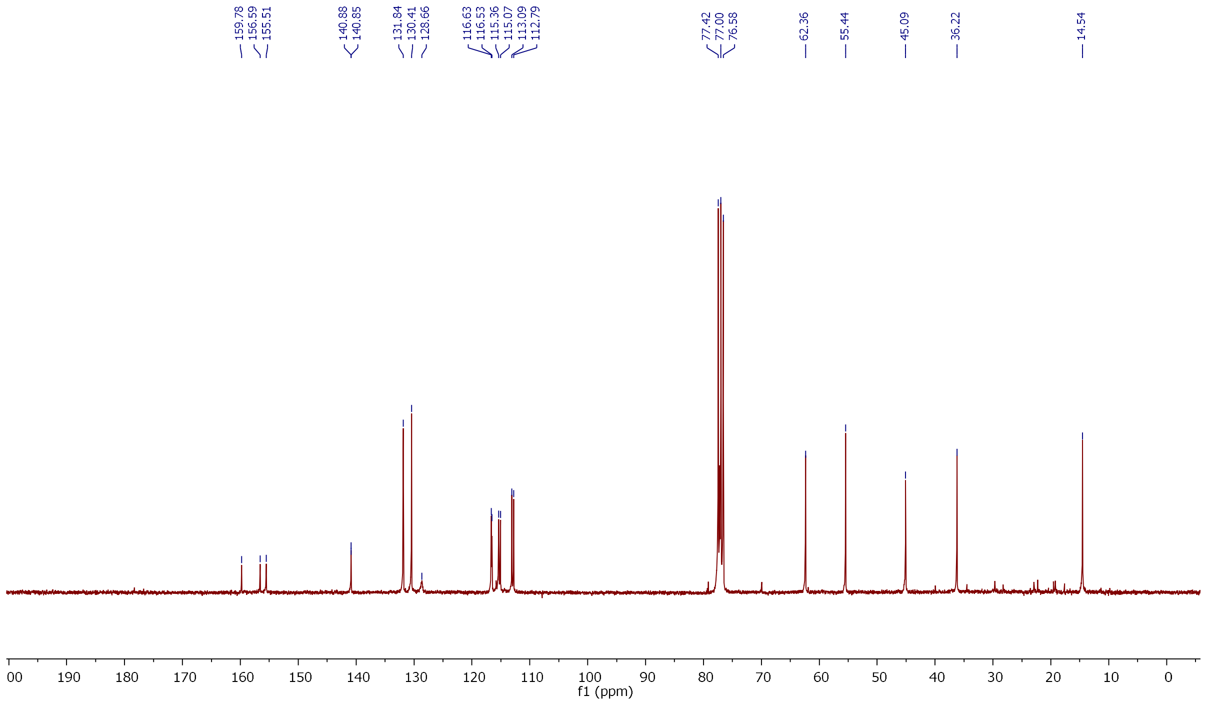

^{19}F (282 MHz, $CDCl_3$) of cinnoline derivative **3d**

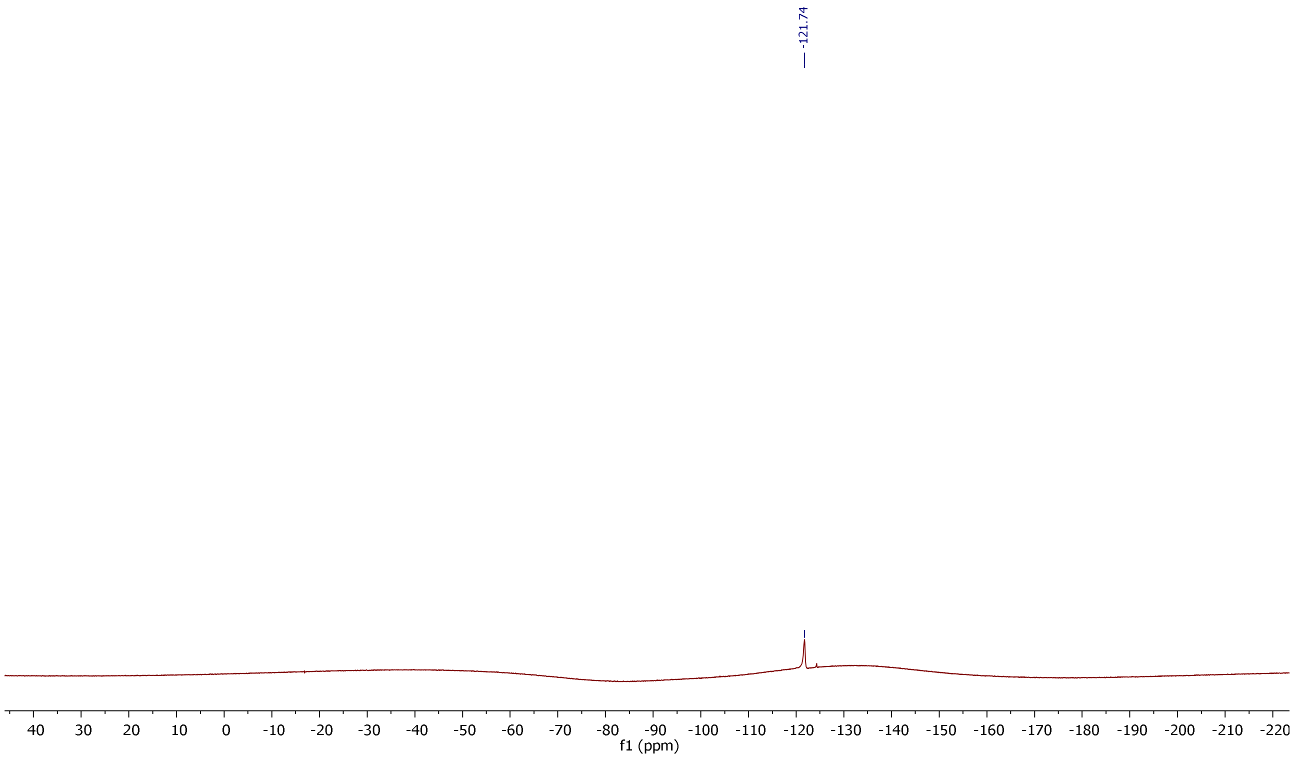

^{1}H NMR (400 MHz, $CDCl_3$) of cinnoline derivative **3e**

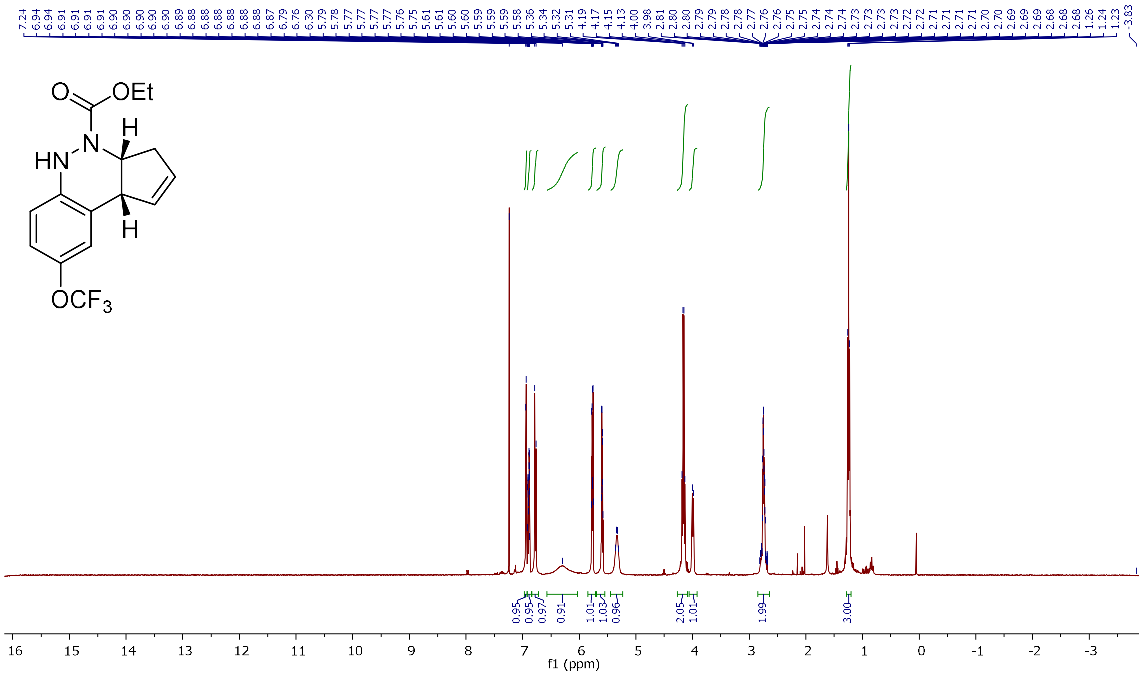

^{13}C {^{1}H} NMR (100 MHz, $CDCl_3$) of cinnoline derivative **3e**

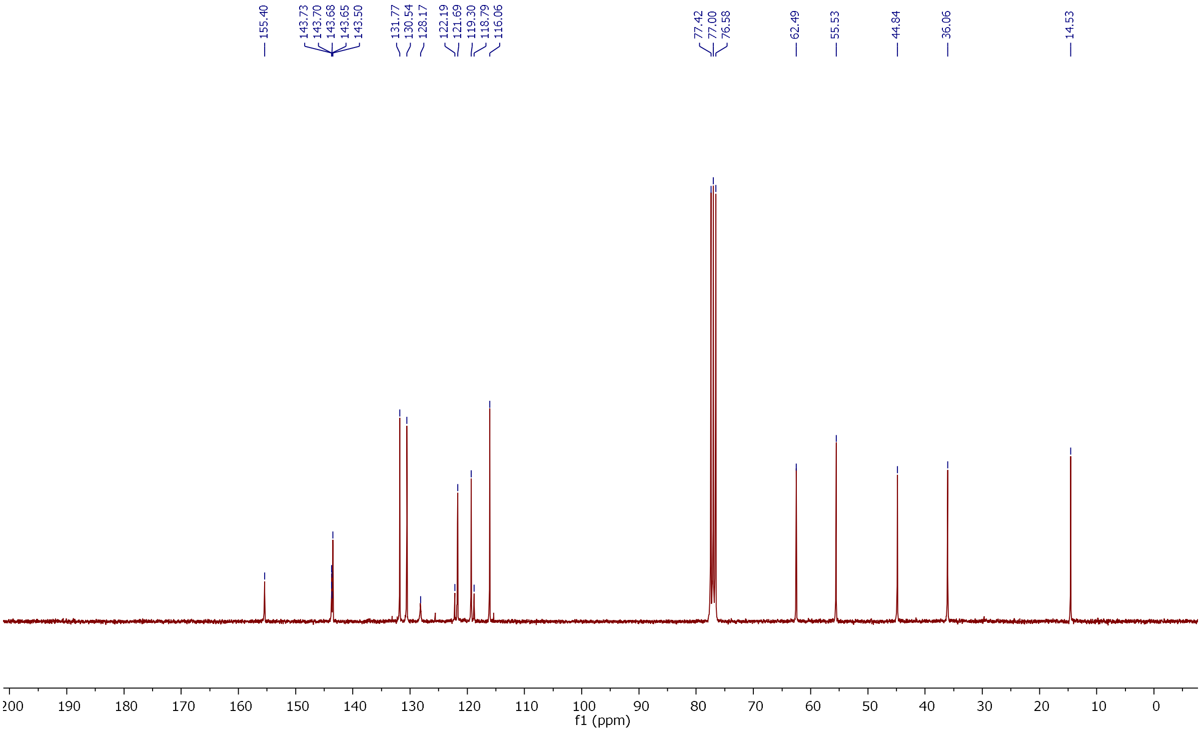

^{19}F (282 MHz, $CDCl_3$) of cinnoline derivative **3d**

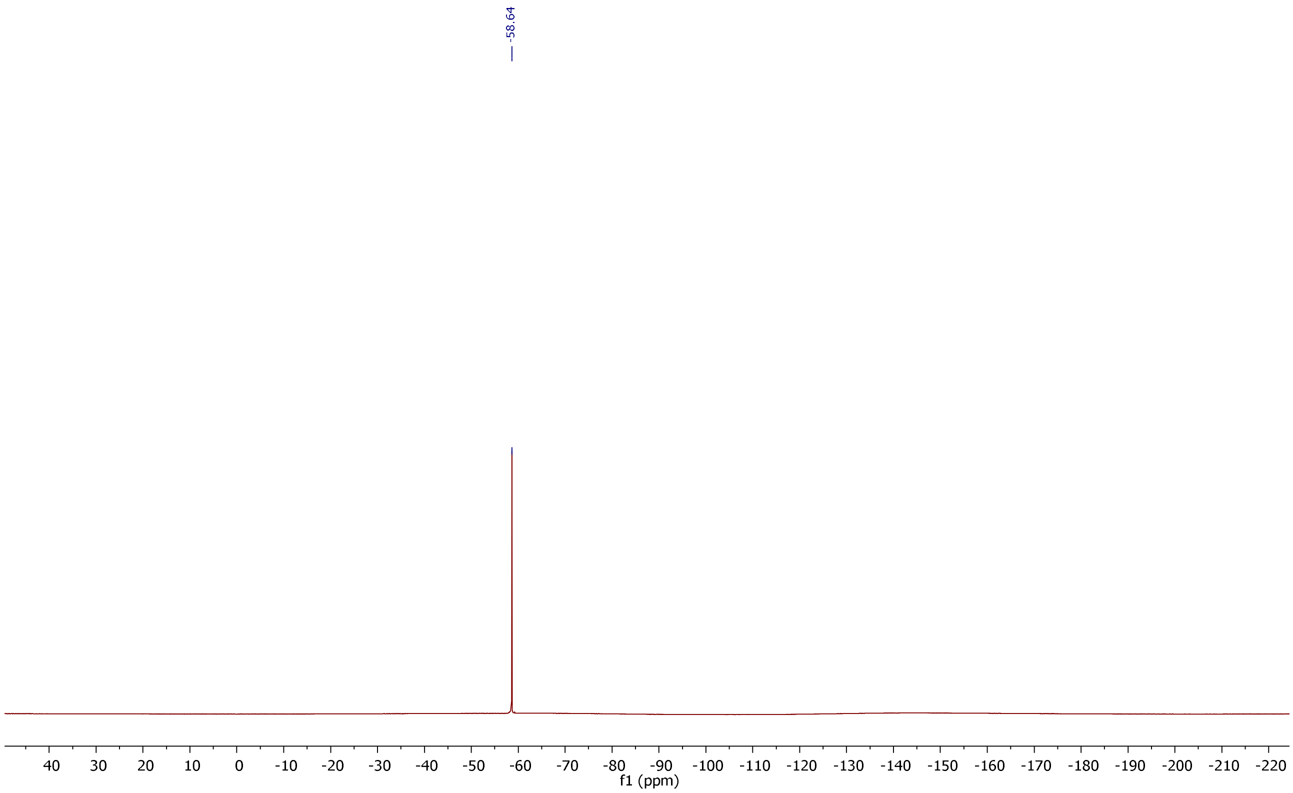

^{1}H NMR (400 MHz, $CDCl_3$) of cinnoline derivative **3f + 3f'**

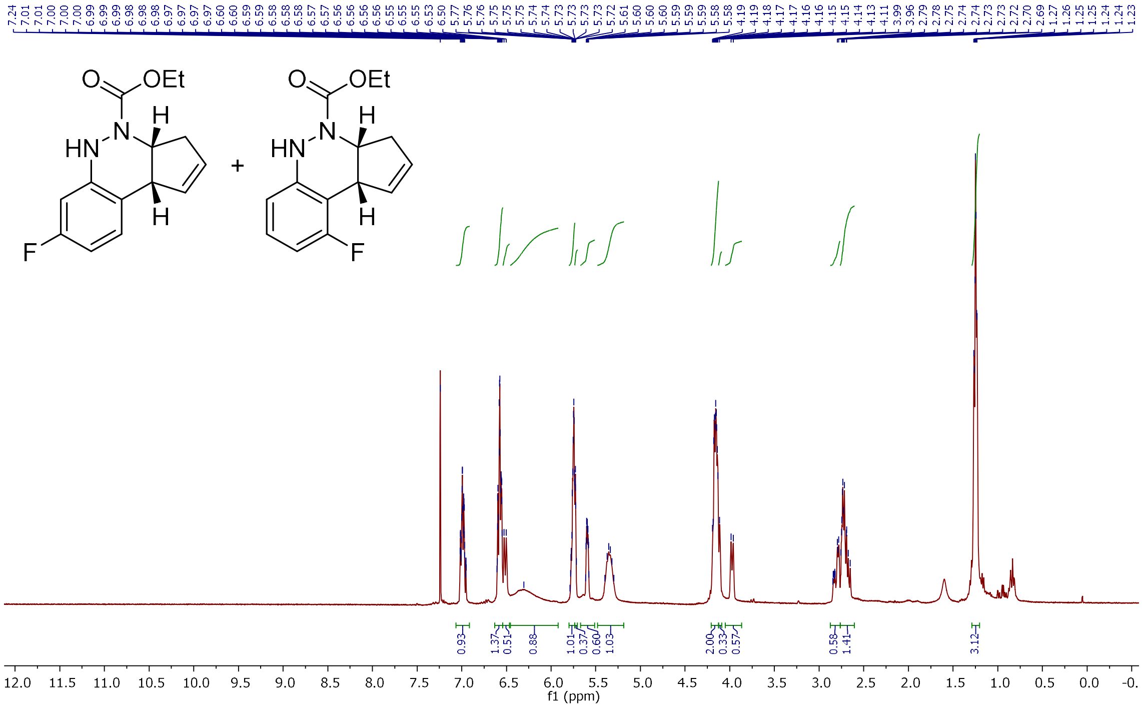

^{13}C {^{1}H} NMR (100 MHz, $CDCl_3$) of cinnoline derivative **3f + 3f'**

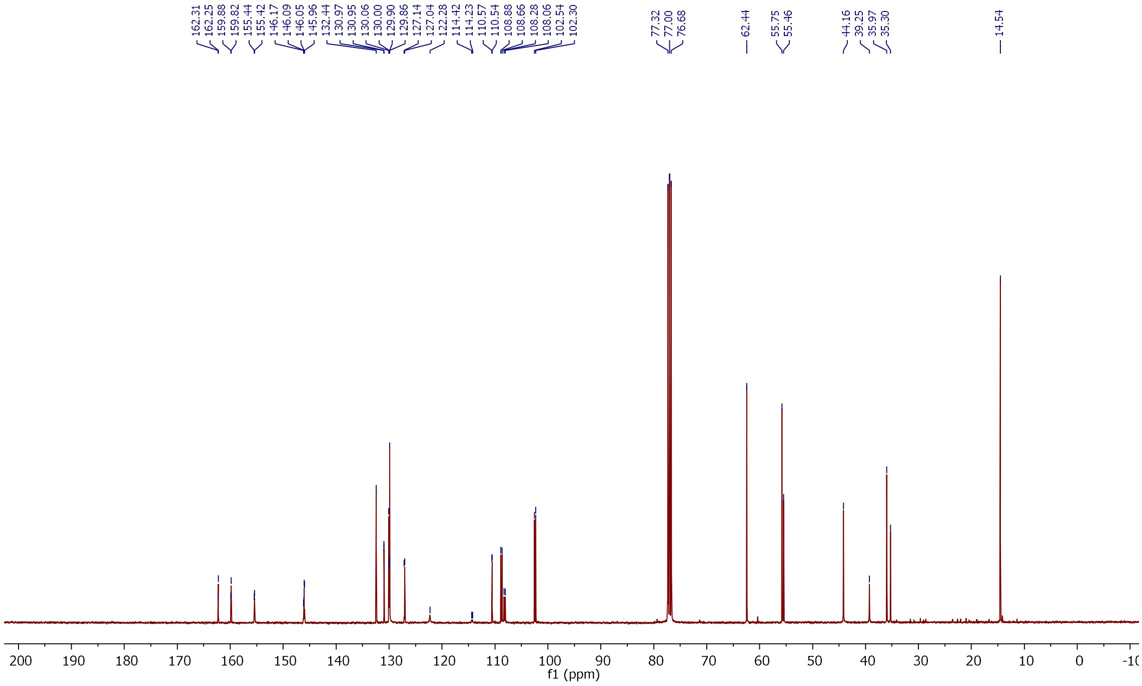

^{19}F (376 MHz, $CDCl_3$) of cinnoline derivative **3f** + **3f’**

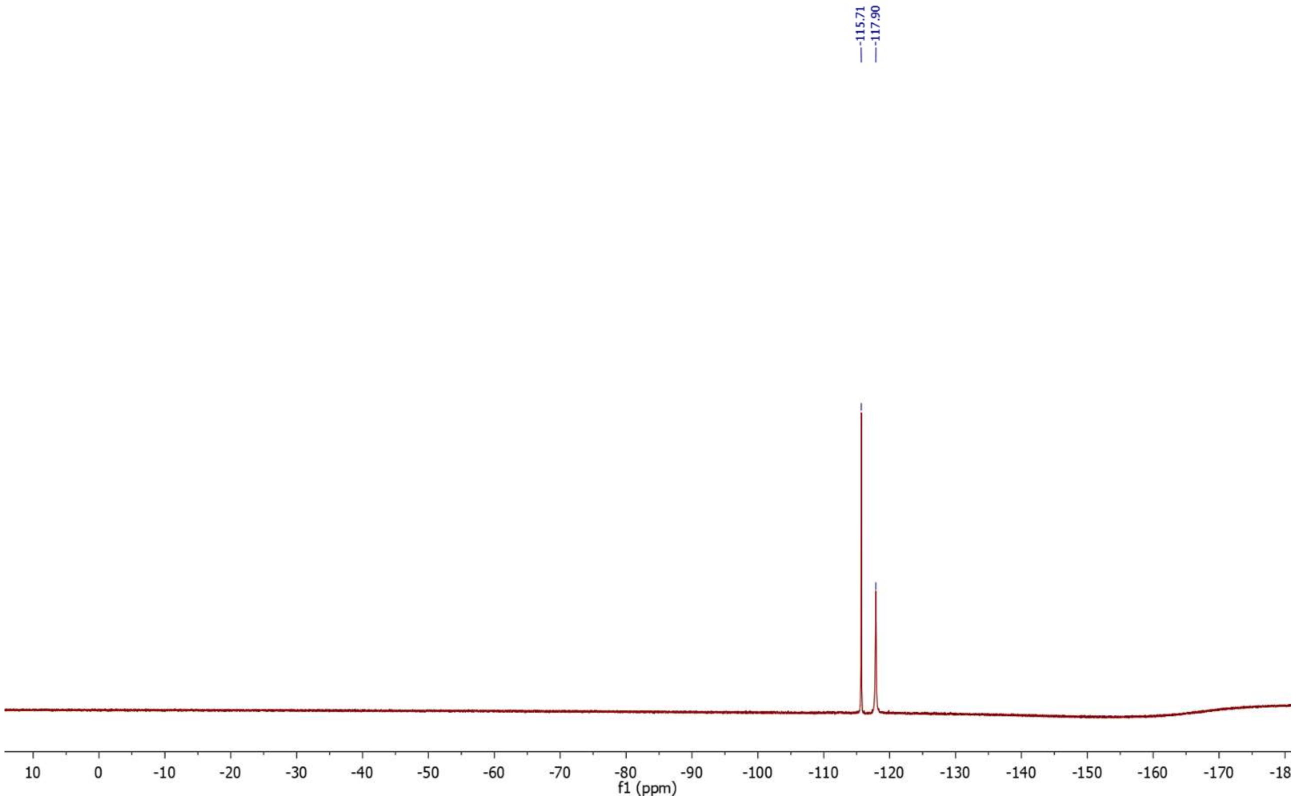

^{1}H NMR (400 MHz, $CDCl_3$) of cinnoline derivative **3g**

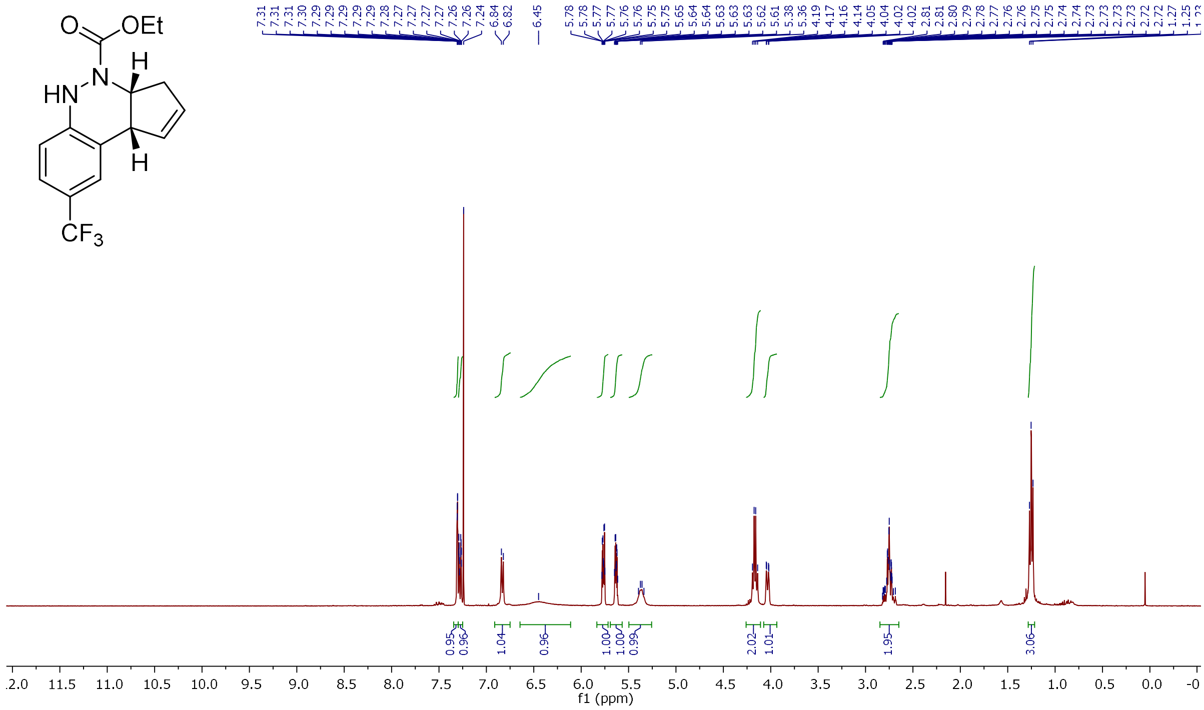

^{13}C {^{1}H} NMR (100 MHz, $CDCl_3$) of cinnoline derivative **3g**

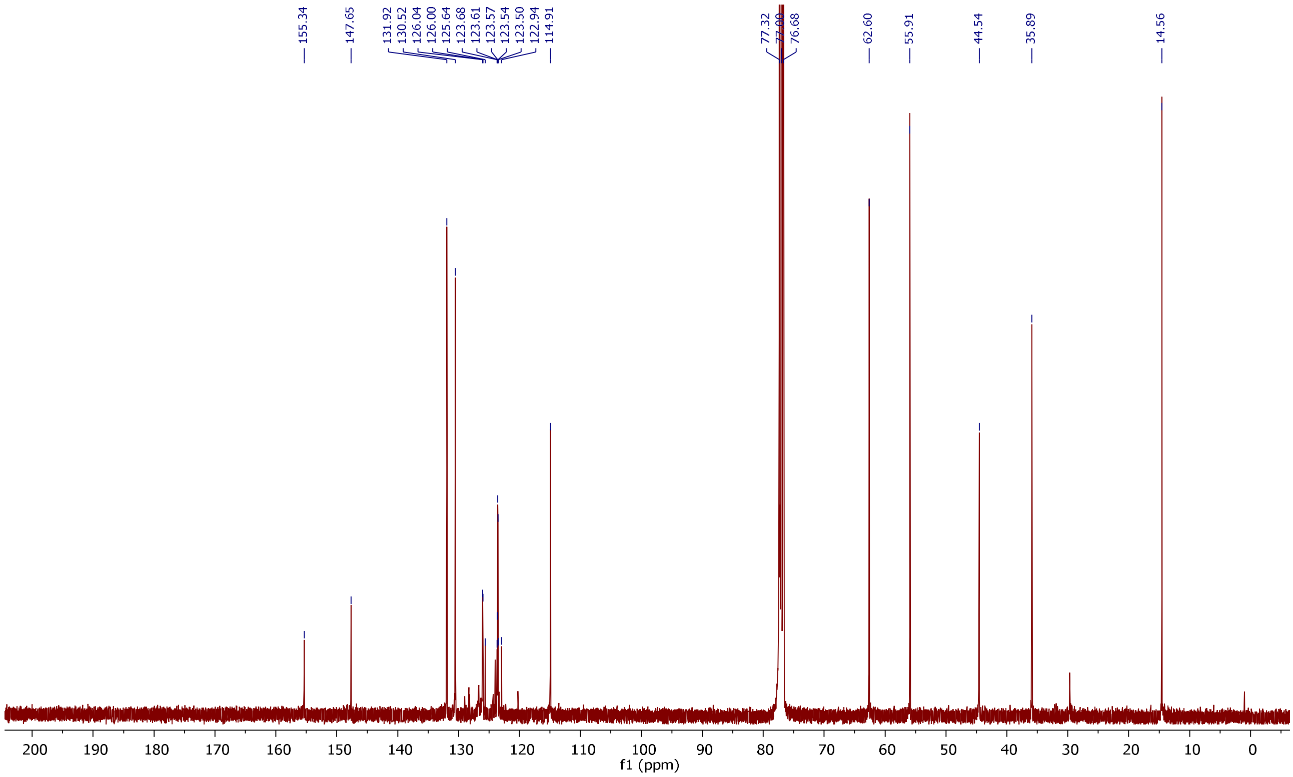

^{19}F (376 MHz, $CDCl_3$) of cinnoline derivative **3g**

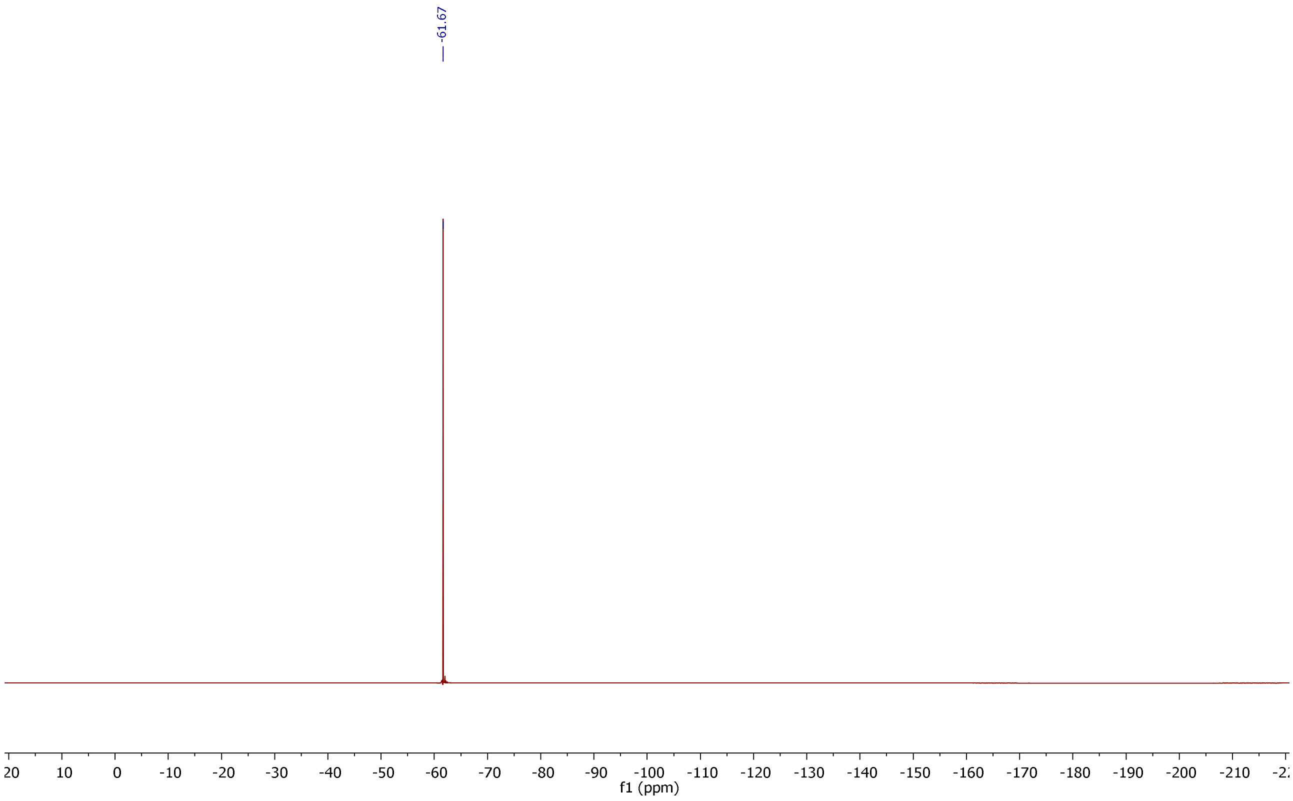

^{1}H NMR (400 MHz, $CDCl_3$) of cinnoline derivative **3h**

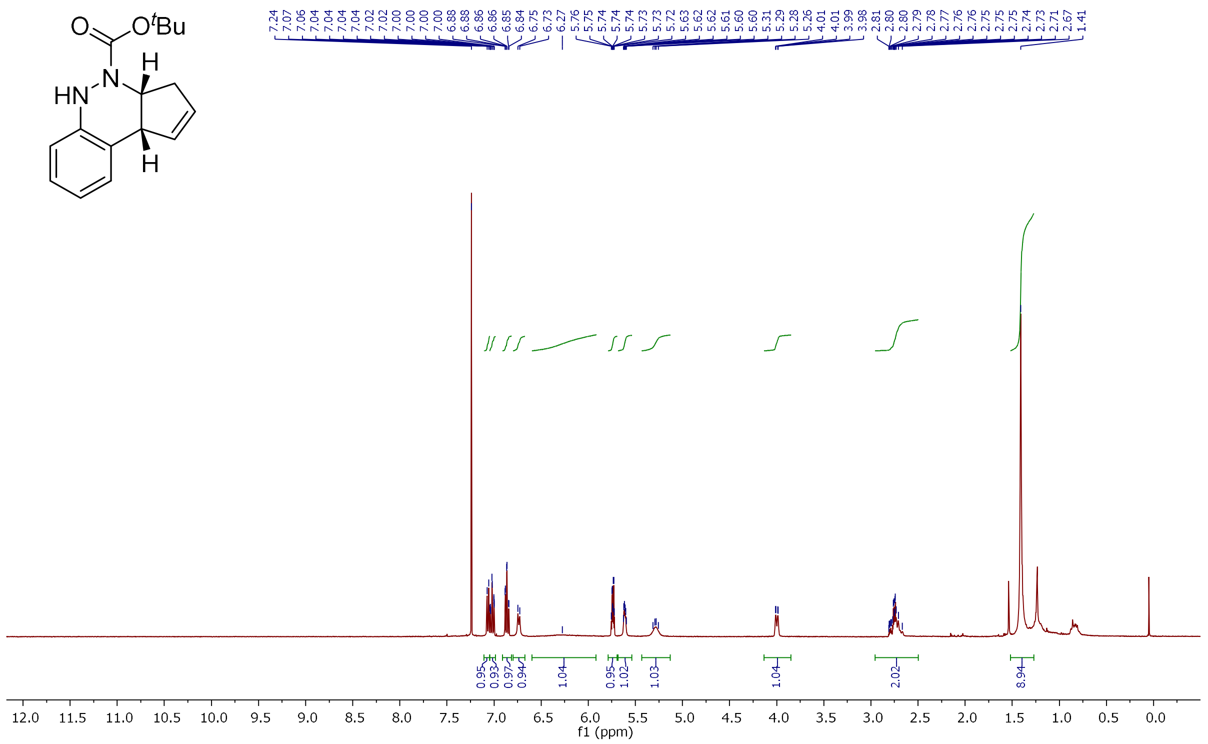

^{13}C {^{1}H} NMR (100 MHz, $CDCl_3$) of cinnoline derivative **3h**

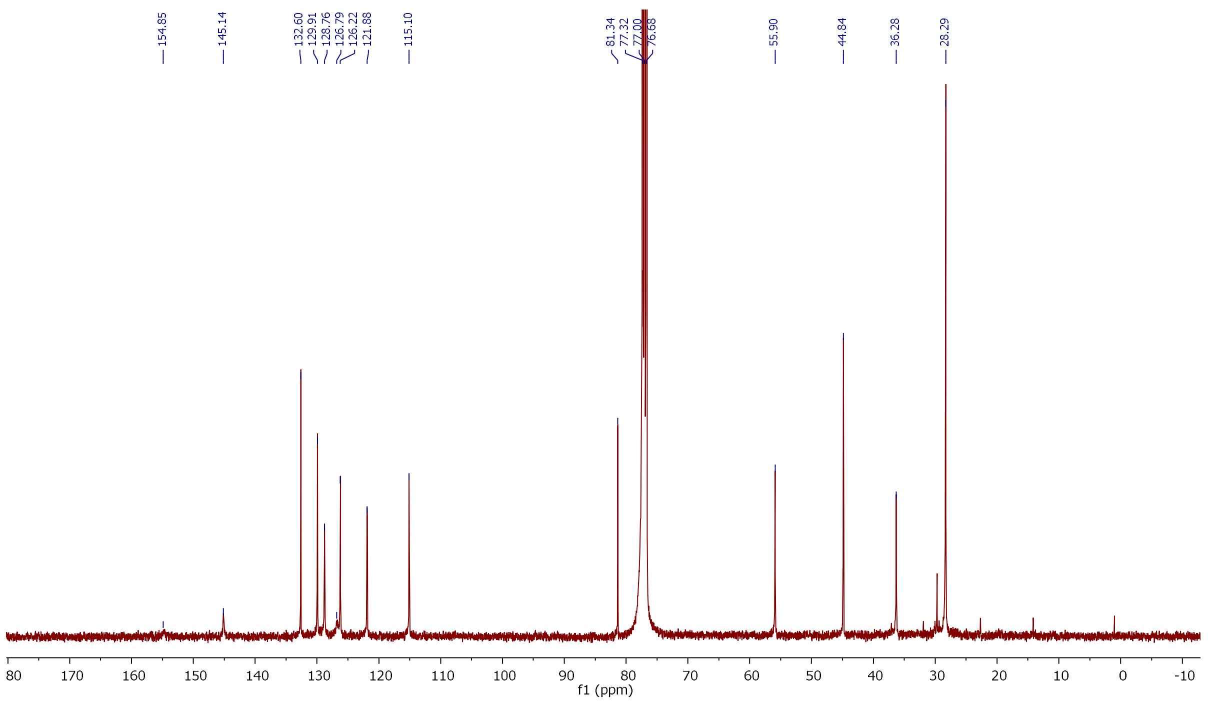

^{1}H NMR (400 MHz, $CDCl_3$) of cinnoline derivative **3i**

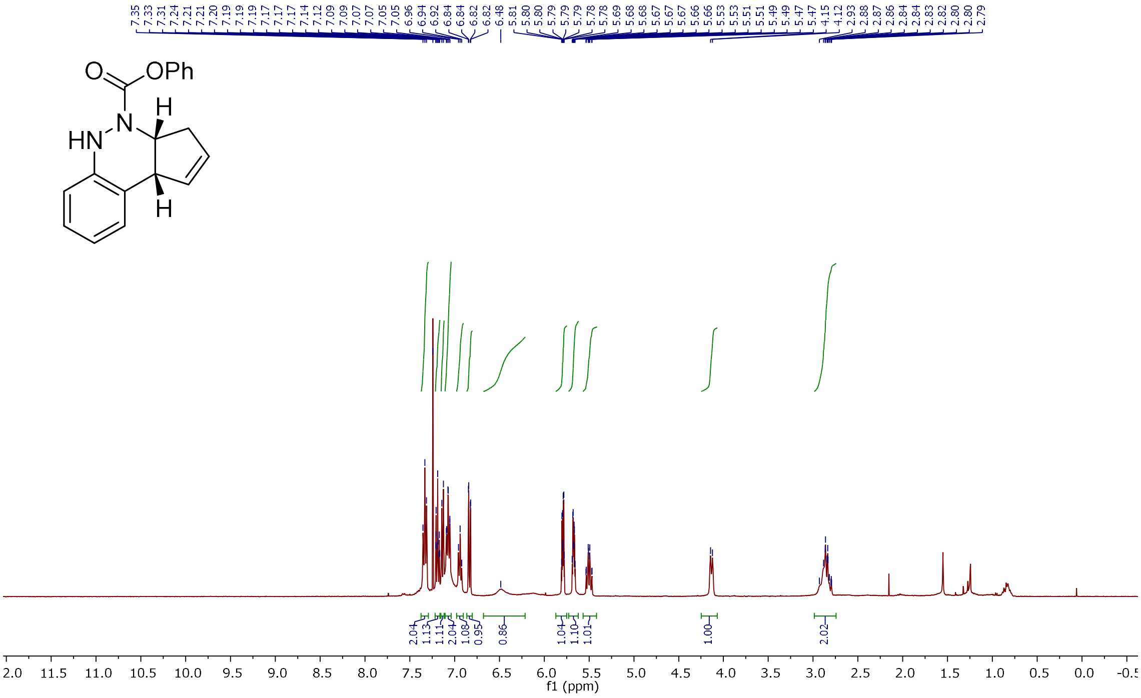

^{13}C {^{1}H} NMR (100 MHz, $CDCl_3$) of cinnoline derivative **3i**

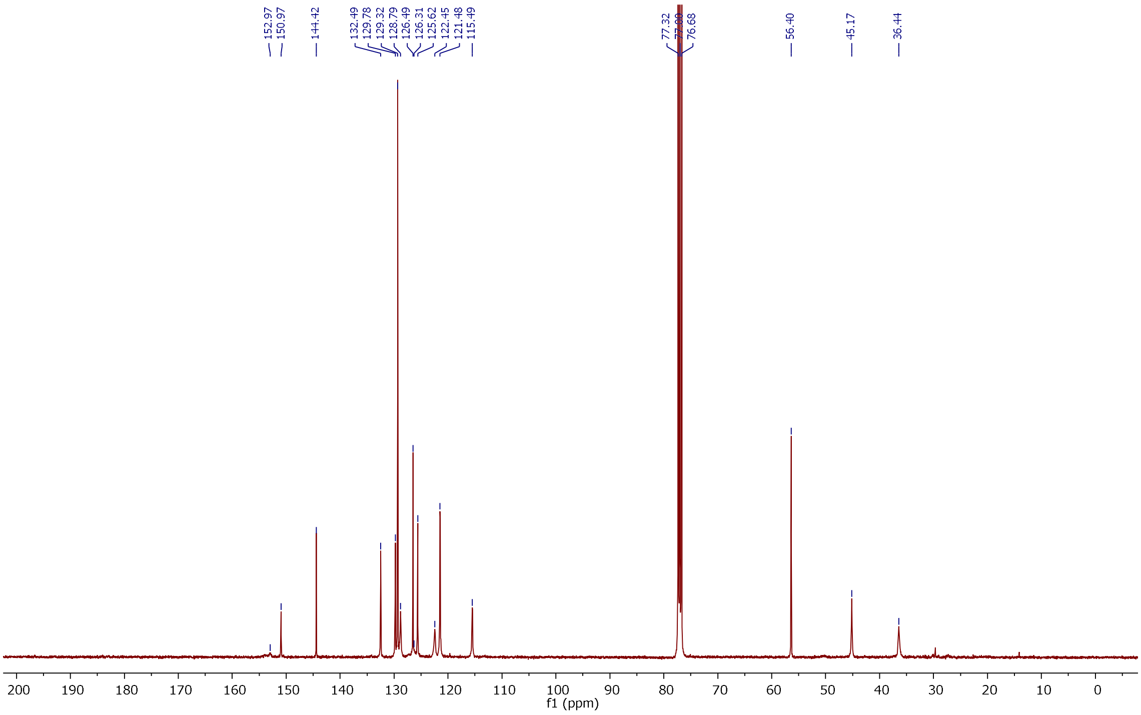

^{1}H NMR (400 MHz, $CDCl_3$) of cinnoline derivative **3j**

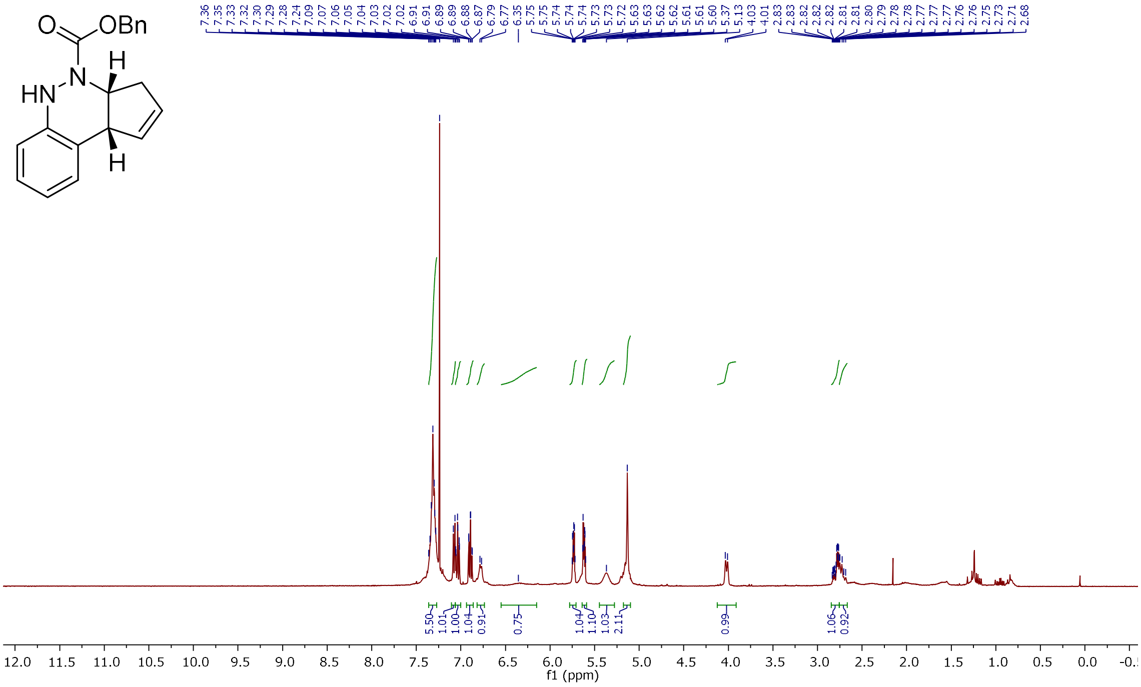

^{13}C {^{1}H} NMR (100 MHz, $CDCl_3$) of cinnoline derivative **3j**

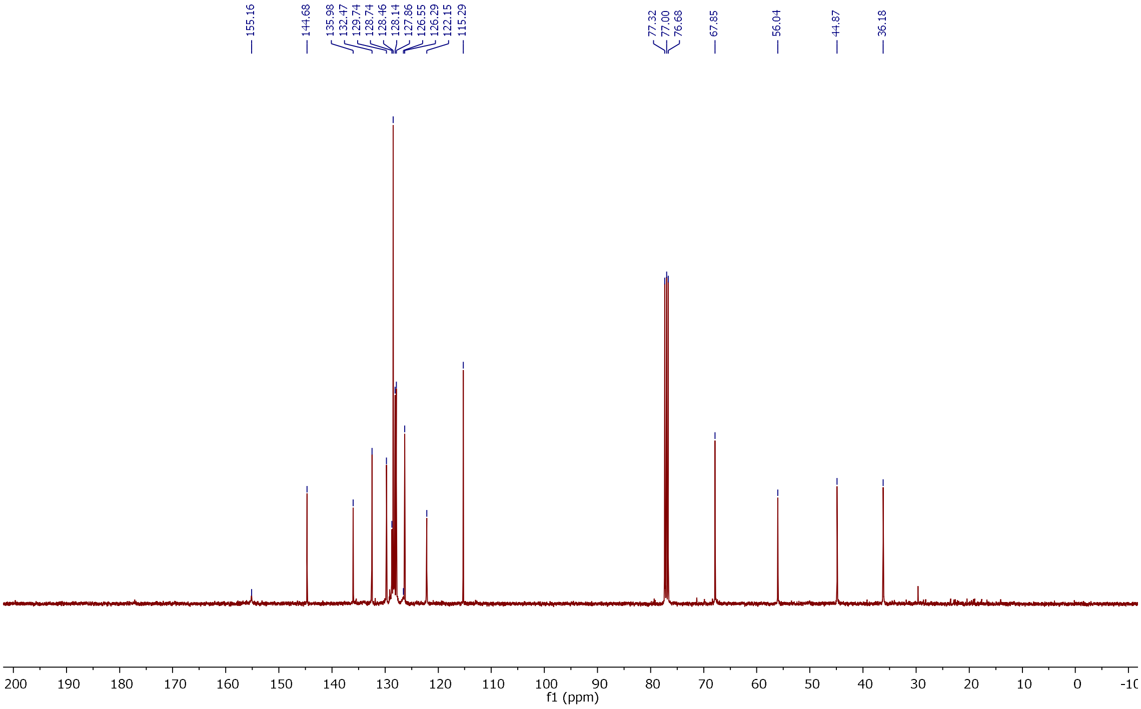

^{1}H NMR (400 MHz, $CDCl_3$) of cinnoline derivative **3k**

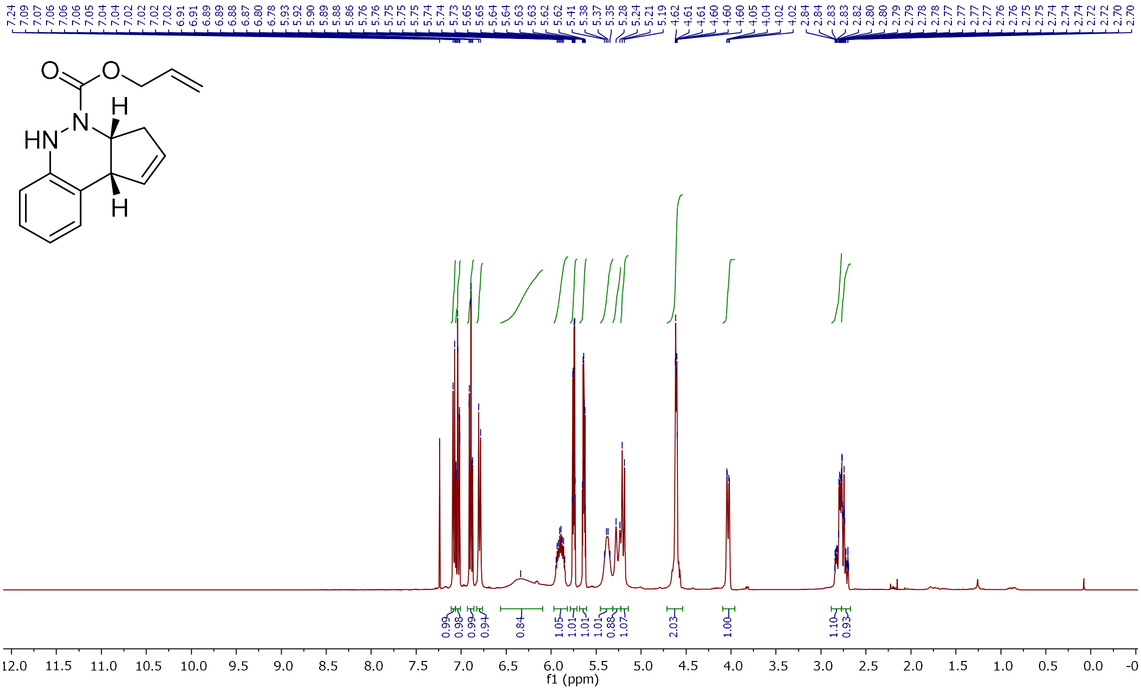

^{13}C {^{1}H} NMR (100 MHz, $CDCl_3$) of cinnoline derivative **3k**

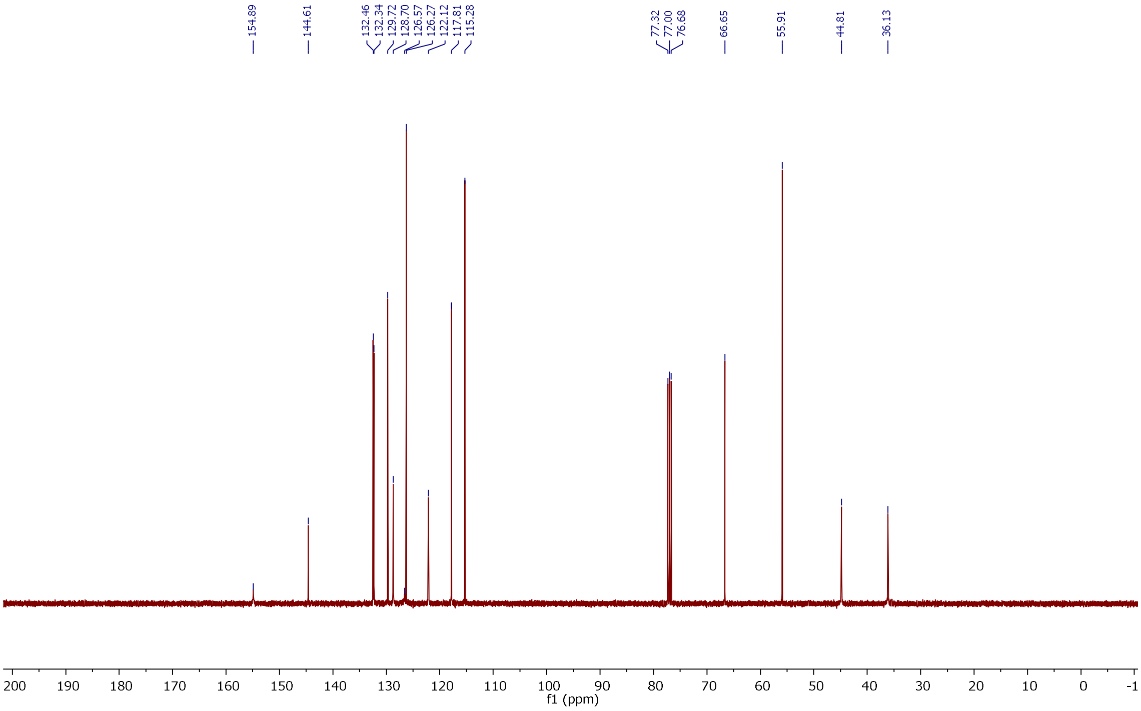

^{1}H NMR (400 MHz, $CDCl_3$) of cinnoline derivative **3l**

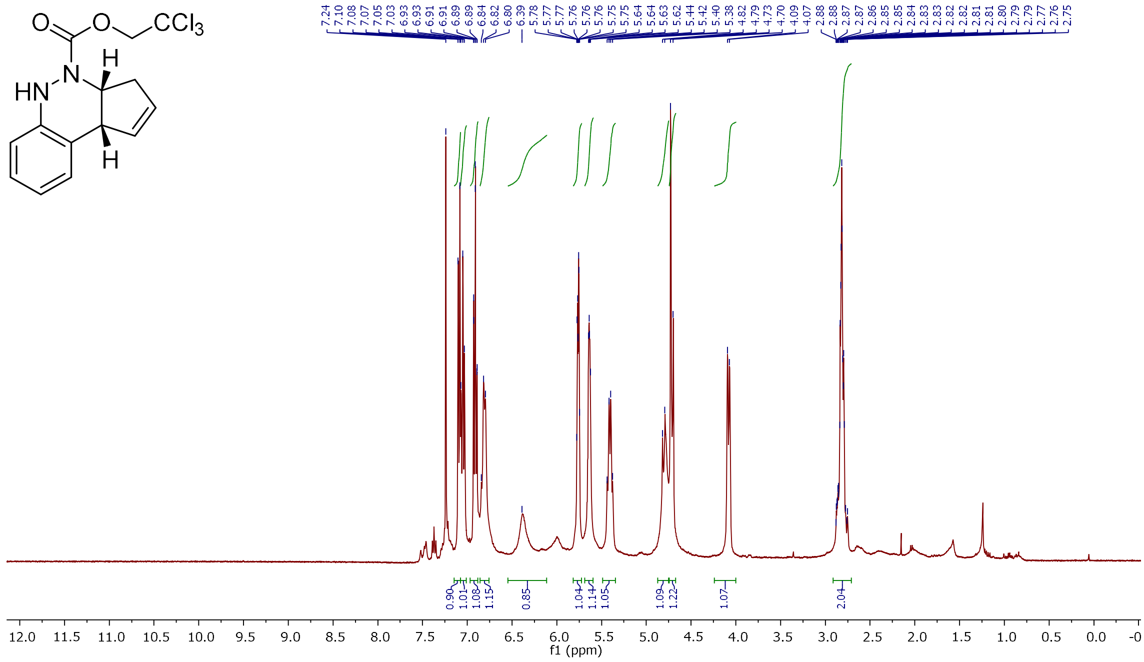

^{13}C {^{1}H} NMR (100 MHz, $CDCl_3$) of cinnoline derivative **3l**

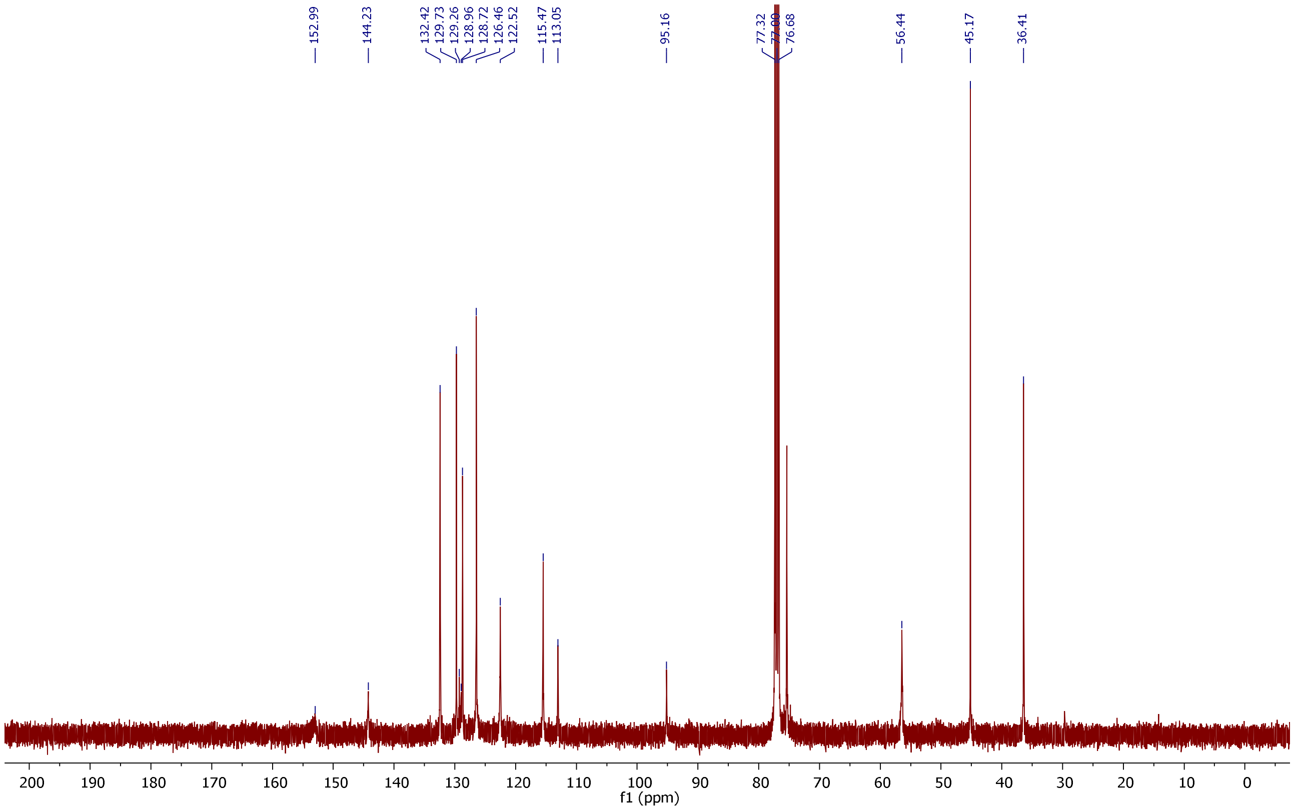

^{1}H NMR (400 MHz, $CDCl_3$) of cinnoline derivatives **3m** + **3m'**

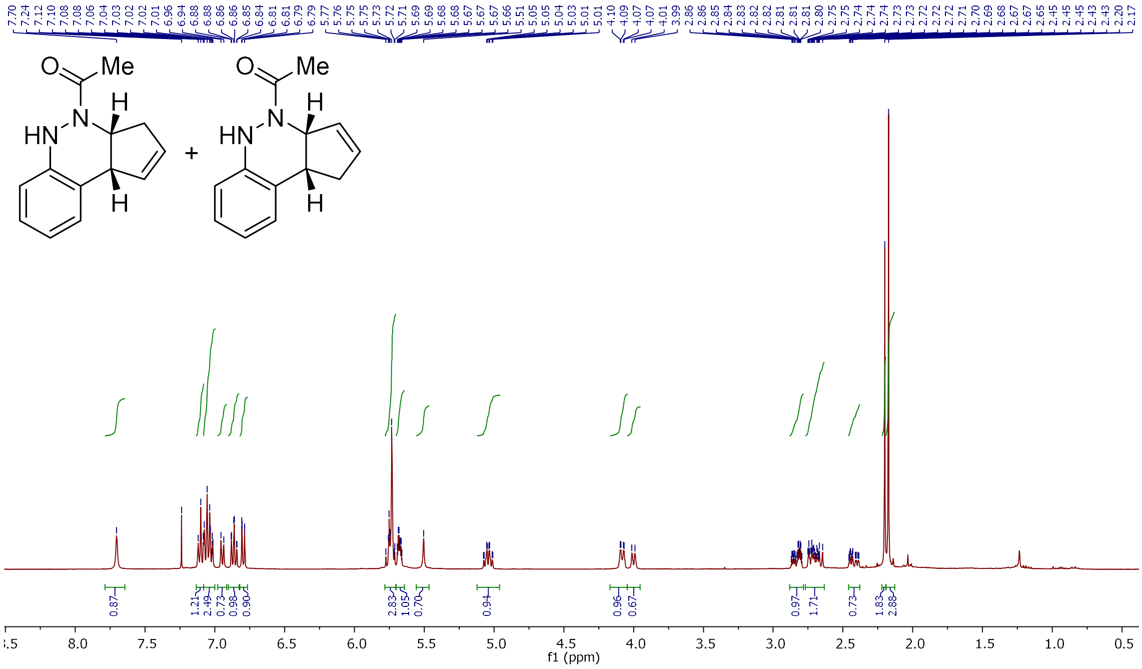

^{13}C {^{1}H} NMR (100 MHz, $CDCl_3$) of cinnoline derivative **3m** + **3m'**

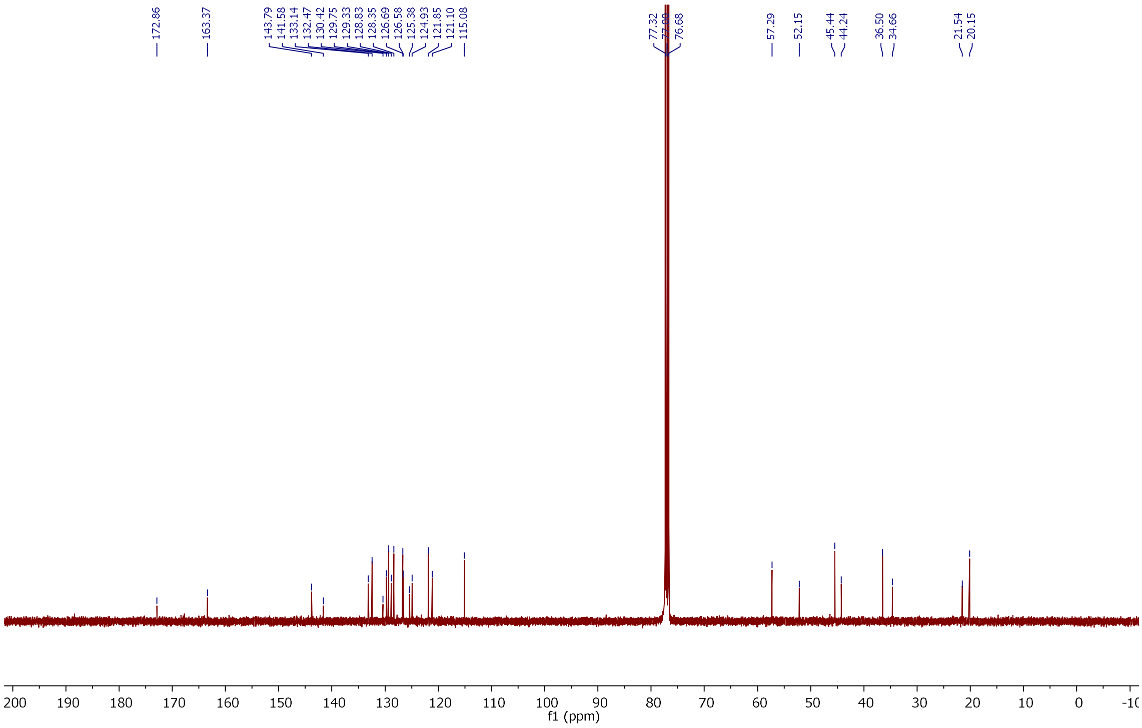

^{1}H NMR (400 MHz, $CDCl_3$) of cinnoline derivatives **3n** + **3n'**

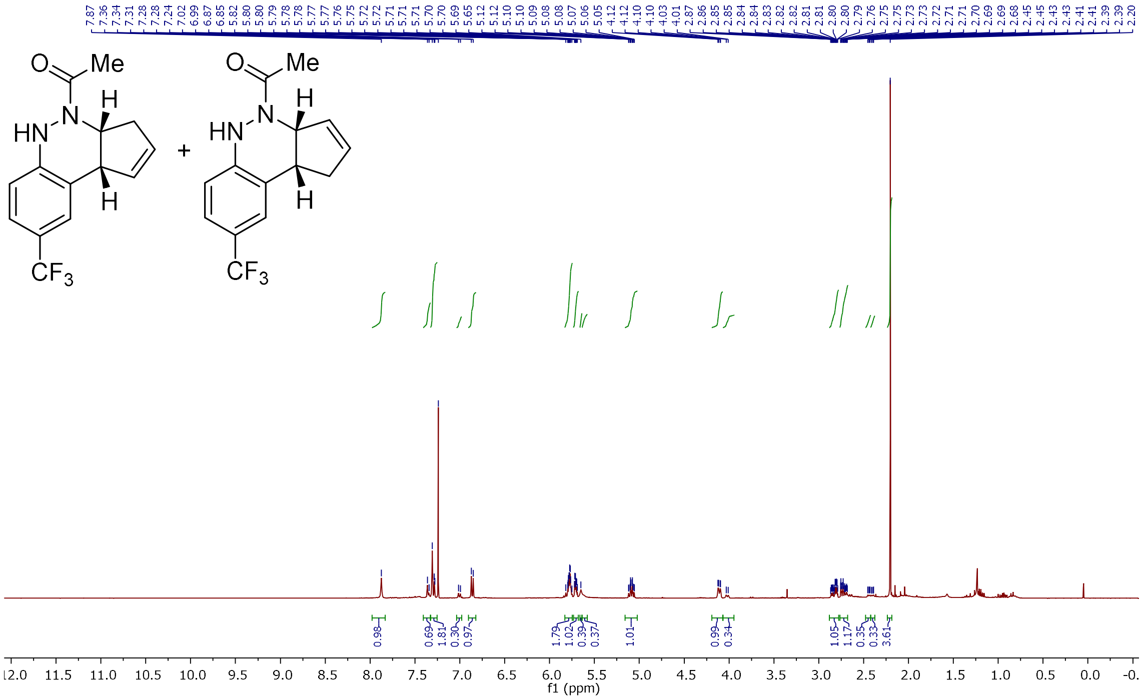

^{13}C {^{1}H} NMR (100 MHz, $CDCl_3$) of cinnoline derivative **3n** + **3n'**

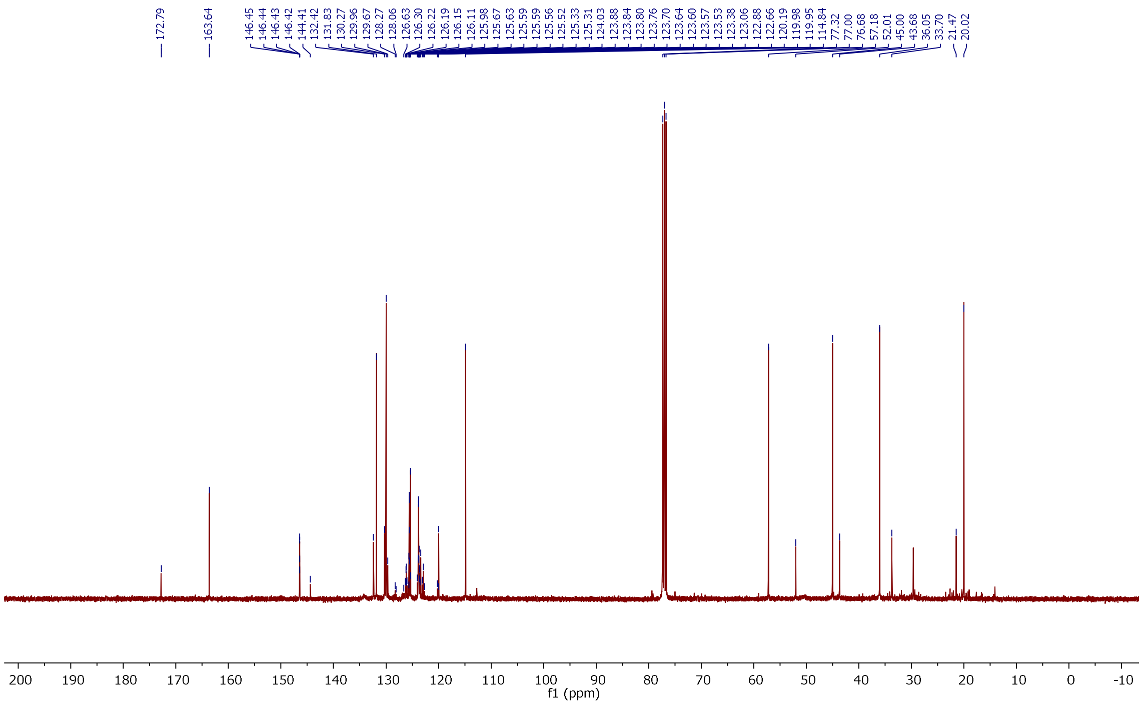

^{19}F (376 MHz, $CDCl_3$) of cinnoline derivative **3n** + **3n'**

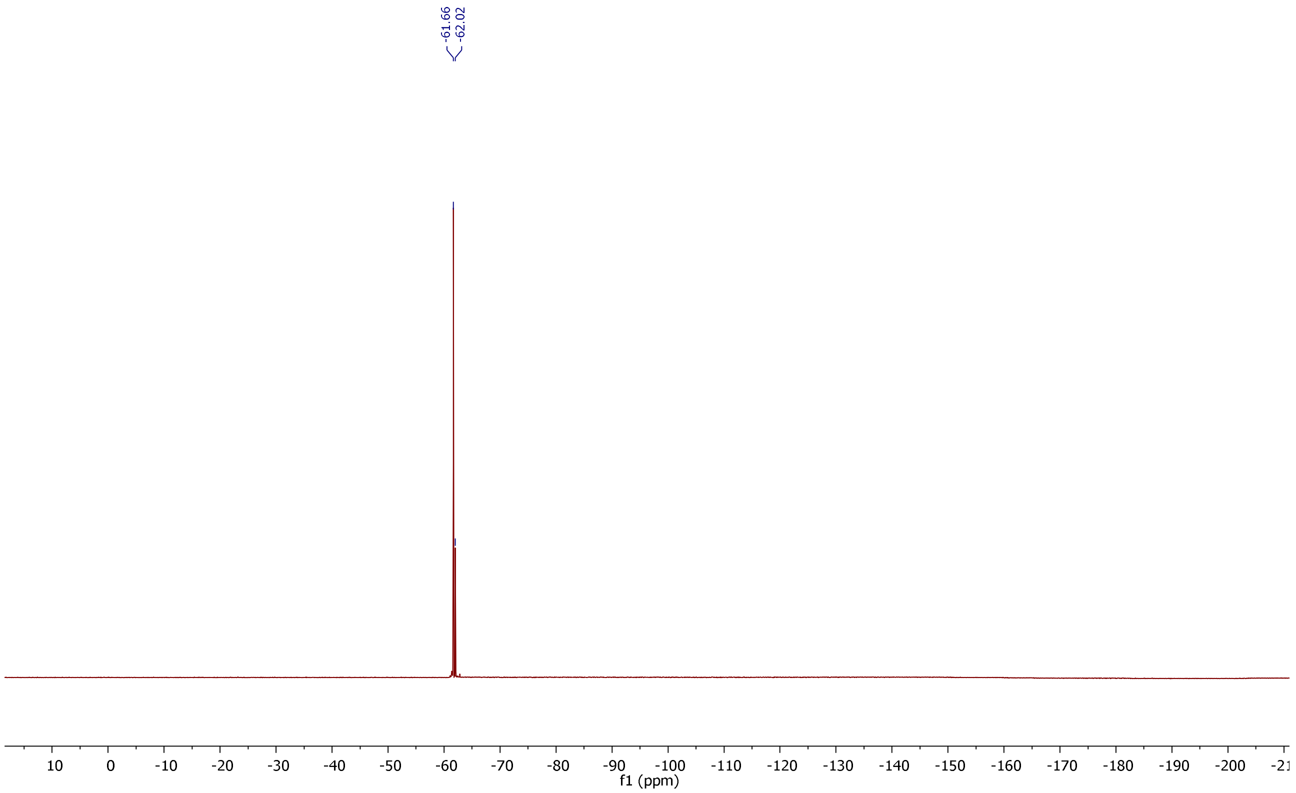

^{1}H NMR (400 MHz, $CDCl_3$) of cinnoline derivative **3o**

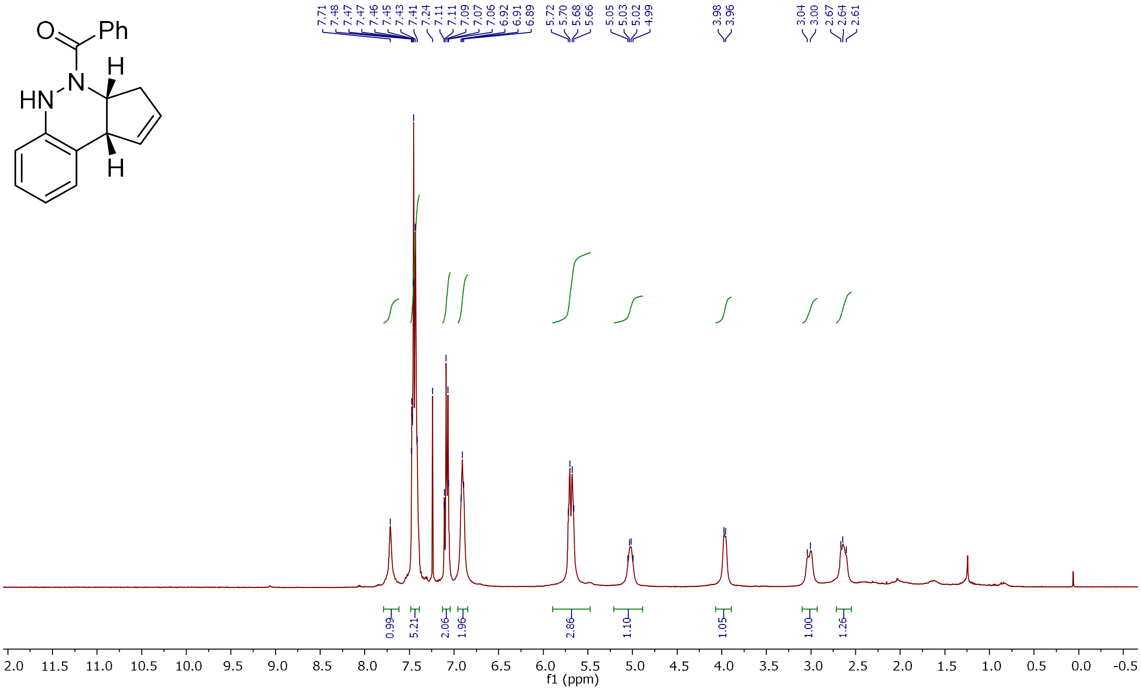

^{13}C {^{1}H} NMR (100 MHz, $CDCl_3$) of cinnoline derivative **3o**

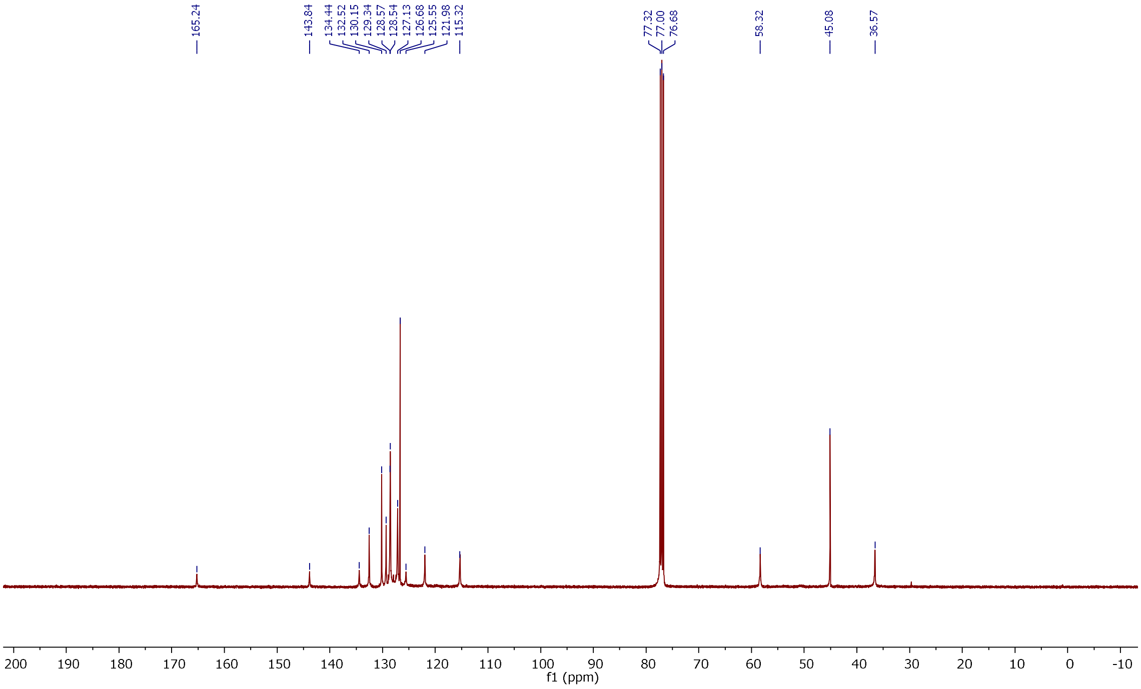

^{1}H NMR (400 MHz, $CDCl_3$) of cinnoline derivative **3p**

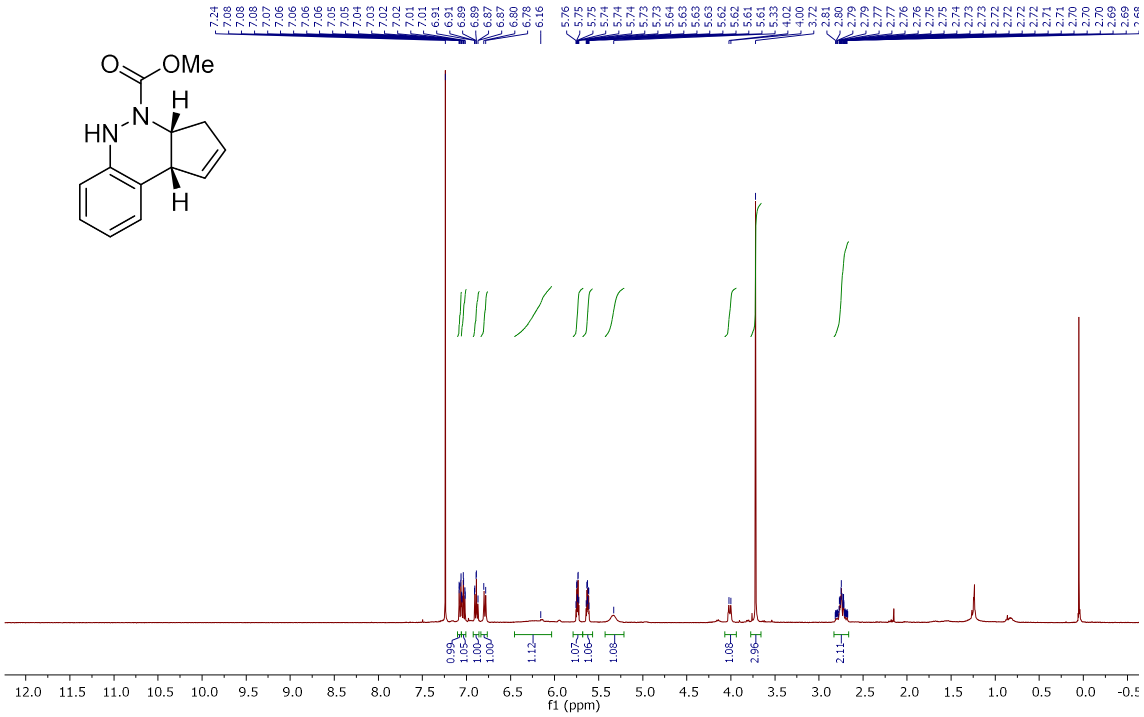

^{13}C {^{1}H} NMR (100 MHz, $CDCl_3$) of cinnoline derivative **3p**

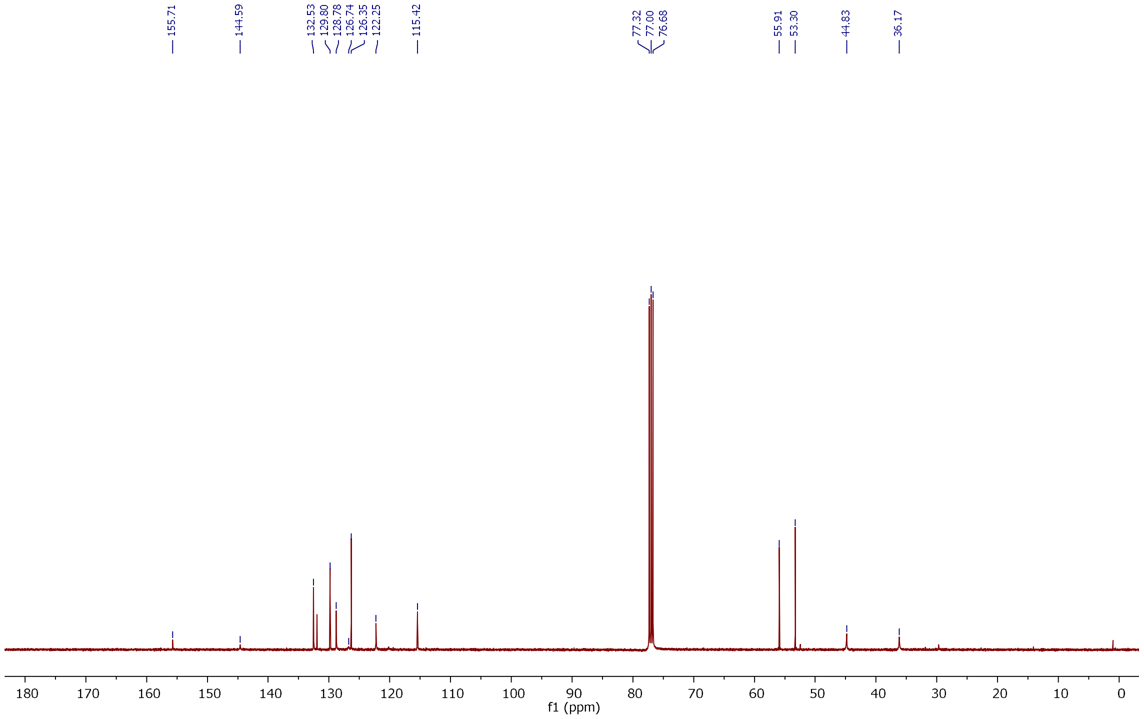

^{1}H NMR (400 MHz, $CDCl_3$) of cinnoline derivative **4**

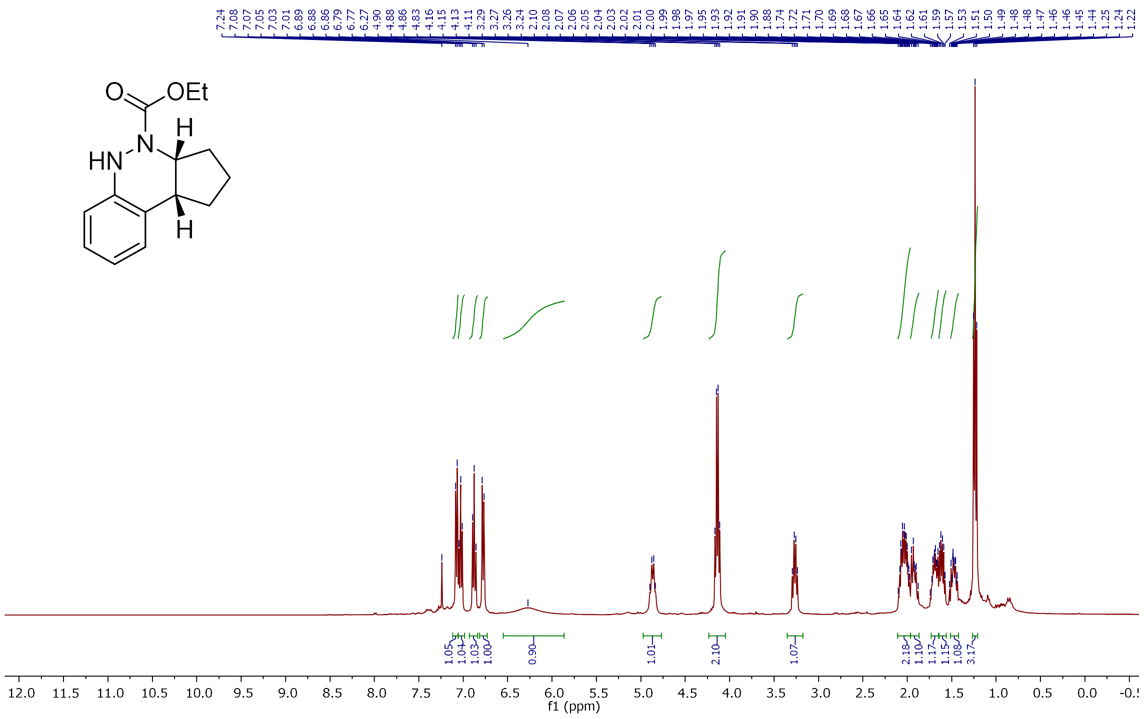

^{13}C {^{1}H} NMR (100 MHz, $CDCl_3$) of cinnoline derivative **4**

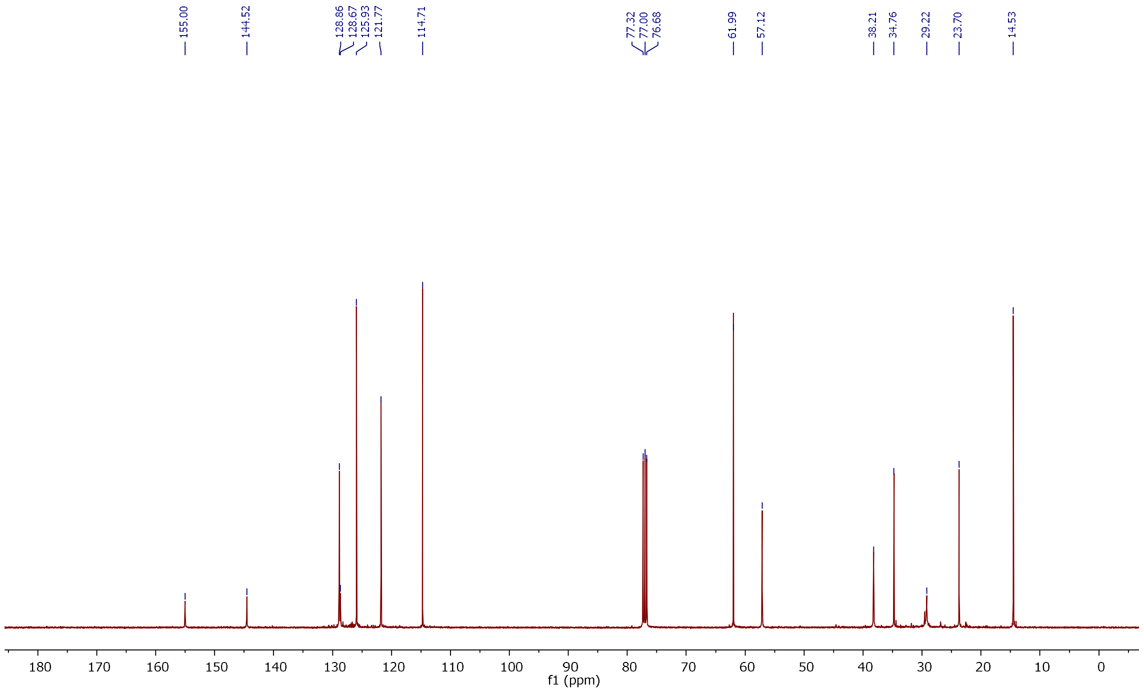

2. ORTEP view and X-ray crystallographic statistics for compounds 3a

(CCDC deposition number 2164540)

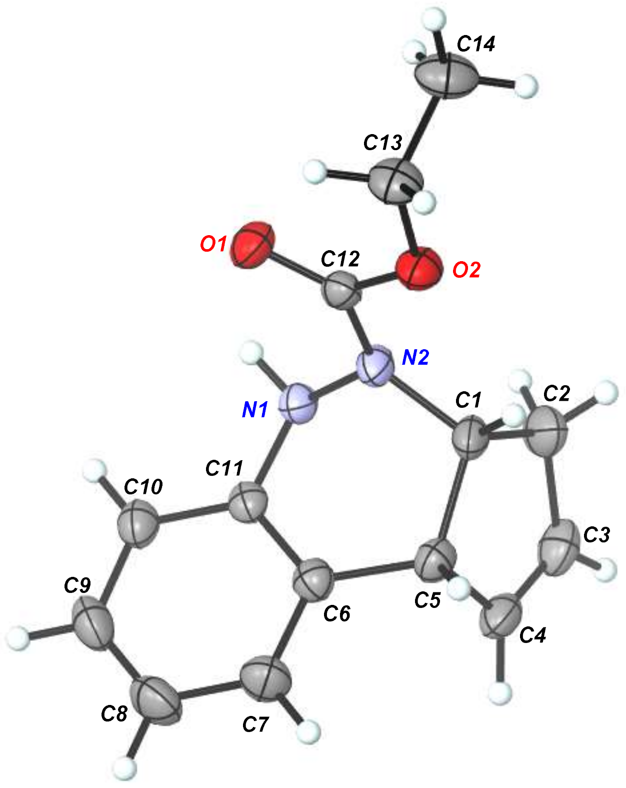

Figure S1. ORTEP diagram of compound **3a** with thermal displacement parameters drawn at 50% probability.

Single crystals of $C_{14}H_{16}N_2O_2$ **[3a]** were obtained. A suitable crystal was selected and mounted on a SuperNova, Dual, Cu at home/near, HyPix diffractometer. The crystal was kept at 170.01(10) K during data collection. Using Olex2,[1] the structure was solved with the ShelXS[2] structure solution program using Intrinsic Phasing and refined with the ShelXL[3] refinement package using Least Squares minimization.

Table S1. Crystal data and structure refinement for **3a**.

Empirical formula	$C_{14}H_{16}N_2O_2$
Formula weight	244.29
Temperature/K	170.01(10)
Crystal system	triclinic
Space group	P–1
a/Å	7.6114(4)
b/Å	8.7423(3)
c/Å	10.4646(5)
α/°	88.424(4)
β/°	79.059(4)
γ/°	66.850(4)
Volume/Å3	627.76(5)
Z	2
ρ_{calc}g/cm^3	1.292
μ/mm^{-1}	0.709
F(000)	260.0
Crystal size/mm^3	0.215 × 0.115 × 0.113
Radiation	CuKα (λ = 1.54184)
2Θ range for data collection/°	8.618 to 137.982
Index ranges	$-9 \leq h \leq 9$, $-10 \leq k \leq 10$, $-12 \leq l \leq 12$
Reflections collected	11214
Independent reflections	2329 [R_{int} = 0.0217, R_{sigma} = 0.0152]
Data/restraints/parameters	2329/3/171
Goodness-of-fit on F^2	1.069
Final R indexes [I>=2σ (I)]	R_1 = 0.0352, wR_2 = 0.0938
Final R indexes [all data]	R_1 = 0.0374, wR_2 = 0.0957
Largest diff. peak/hole / e Å^{-3}	0.19/-0.18

3. References

[1] Dolomanov, O.V.; Bourhis, L.J.; Gildea, R.J.; Howard, J.A.K.; Puschmann, H. *J. Appl. Cryst.* **2009**, *42*, 339–341.

[2] Sheldrick, G. M. *Acta Cryst*. **2008**, *A64*, 112–122.

[3] Sheldrick, G. M. *Acta Cryst*. **2015**, *C71*, 3–8.
